# Digging for the discovery of SARS-CoV-2 nsp12 inhibitors: a pharmacophore-based and molecular dynamics simulation study

**DOI:** 10.2217/fvl-2022-0054

**Published:** 2022-08-08

**Authors:** Fatemeh Sana Askari, Mohsen Ebrahimi, Jabbar Parhiz, Mina Hassanpour, Alireza Mohebbi, Abbas Mirshafiey

**Affiliations:** ^1^Vista Aria Rena Gene Inc., Gorgan, 4918653885, Golestan Province, Iran; ^2^Neonatal & Children's Health Research Center, Golestan University of Medical Sciences, Gorgan, 4918936316, Iran; ^3^Department of Immunology, School of Public Health, Tehran University of Medical Sciences, Tehran, 1417613151, Iran

**Keywords:** molecular docking, molecular dynamics, natural product, pharmacophore-based drug discovery, RNA-dependent RNA polymerase, SARS-CoV-2

## Abstract

**Aim:** COVID-19 is a global health threat. Therapeutics are urgently needed to cure patients severely infected with COVID-19. **Objective:** to investigate potential candidates of nsp12 inhibitors by searching for druggable cavity pockets within the viral protein and drug discovery. **Methods:** A virtual screening of ZINC natural products on SARS-CoV-2 nsp12's druggable cavity was performed. A lead compound with the highest affinity to nsp12 was simulated dynamically for 10 ns. **Results:** ZINC03977803 was nominated as the lead compound. The results showed stable interaction between ZINC03977803 and nsp12 during 10 ns. **Discussion:** ZINC03977803 showed stable interaction with the catalytic subunit of SARS-CoV-2, nsp12. It could inhibit the SARS-CoV-2 life cycle by direct interaction with nsp12 and inhibit RdRp complex formation.

SARS-CoV-2, the etiological agent of COVID-19, is an ongoing pandemic that has led to over 200 million infections and more than 4 million deaths around the world till 15 August 2021 (https://www.worldometers.info/coronavirus/). A single-stranded, positive-sense RNA genome of ∼30 kb codes for 14 open reading frames (ORFs) [1]. At least 26 proteins are responsible for the structural, and non-structural proteins (nsp), and accessory proteins are required for the viral assembly, replication, and polyprotein modifications. Two ORFs, ORF1a and ORF1ab, are the largest transcriptional regions on the SARS-Cov-2 genome that are enclosed at both ends by two untranslated regions (UTR). The whole genome of the virus is comprised of two overlapping polyproteins denoted pp1a and pp1ab. These two polyproteins are post-processed by two virally encoded accessory enzymes, PLpro (nsp3) and Mpro (nsp5), into 16 nsp (nsp1-16). The rest of the genome encodes 4 structural Spike (S), Envelope (E), Matrix (M), and Nucleocapsid (N) proteins, as well as six accessory proteins. All three categories of SARS-CoV-2 proteins have been targeted for antiviral measures. Structural S protein, which is responsible for the virus binding to the host cell receptor ACE2, has been one of the essential viral proteins for therapeutic studies [2–4]. Nsp3 and nsp5 proteases, as two mail accessory proteins, are also targeted for anti-COVID-19 studies [5–7]. Among all non-structural proteins, an evolutionary conserved catalytic subunit of viral RNA-dependent RNA polymerase (RdRp), nsp12, is suitable for designing effective therapeutic candidates [1,8]. RdRp is responsible for replication and transcription of the viral genome and is a very interesting target for *in silico* drug discovery methods. Therefore, targeting the nsp12 active site might suppress the formation of the RdRp complex, incorporate free nucleotides, translocation the residing nucleotides, and suppress the proofreading process.

As a suitable drug discovery target, the catalytic nsp12 subunit of the RdRp complex is comprised of 932 amino acid residues and consists of an N-terminal nidovirus-unique RdRp-associated nucleotidyltransferase (NiRAN) domain (amino acids 60–249) and RdRp domain (366–920). The RdRp domain is contained three fingers, palm, and thumb subunits [9]. The only US FDA-approved therapeutic for COVID-19 is the active remdesivir triphosphate (RTP), which stalls RdRp from the growing RNA. The mechanism by which remdesivir inhibits viral polymerization is induced in both RNA-dependent and -independent mechanisms [10]. Molnupiravir is another nucleoside analogue in the clinical trial phase II and is recognized by cell-free CoV-RdRp-Gluc assay to inhibit the RdRp more efficiently than remdesivir [11]. The nsp12 is prone to high mutations [12], but the emergence of escape mutations at the catalytic nsp12 subunit is rare and very conserved among beta-coronaviruses [13]. Therefore, nucleot(s)ide therapy to inhibit the SARS-CoV-19 RdRp seems reasonable. However, recent findings demonstrated that exoribonuclease proofread activity of nsp14 co-expressed with nsp10 confers high resistance to nucleot(s)ide therapy *in vitro* [11]. To overcome this barrier of using nucleot(s)ide analogues, Wang *et al.* used a complex therapy of hepatitis C virus (HCV) NS5A inhibitors, pibrentasvir or ombitasvir, to inhibit the SARS-CoV-2 exonuclease in combination with sofosbuvir or remdesivir to inhibit nsp12 activity [14]. Furthermore, the binding of the small molecules to the targeted protein is more stable and less affected by nucleotide substitutions. Therefore, further studies on the discovery of potent nsp12 inhibitors are still ongoing. In this regard, Khan *et al.* used a multi-step computational approach to identify direct-acting potential lead-like compounds to inhibit nsp12 [15]. The virtual screening and molecular dynamic simulation (MDS) of identified hits from the Northern South African medicinal compounds database (NANPDB) comprising 6482 natural compounds showed that Diosmetin-7-O-beta-d-apiofuranoside had a higher affinity (-10.4 kcal.mol^-1^) to nsp12 than that of remdesivir [15]. Additionally, Zhao *et al.* performed an *in silico* screening of a small herbal database to find potential direct-acting compounds against different non-structural SARS-CoV-2 targets. Accordingly, Amentoflavone with the highest affinity (-9.7 kcal.mol^-1^)was highlighted as an nsp12 inhibitor [16]. Zhang *et al.* translated the results of computational screening of 1906 approved drugs into *in vitro* assays. Their findings showed Pralatrexate with an EC_50_ value of 8 nM potentially inhibits SARS-CoV-2 replication [17]. Further studies on inhibiting SARS-CoV-2 replication blockers were also reported. In this regard, pyridobenzothiazole derivatives with an EC_50_ range of 0.5 to >50 μM are shown to inhibit SARS-CoV-2 nsp12 [18].

The rise of highly contagious SARS-CoV-2 strains has challenged the vaccination efficiency due to the large gap in vaccination interval in developed countries and developing/under developing countries with higher population density. Accordingly, therapeutic approaches are necessitated to improve the mortality rates of acute SARS-CoV-2 infections. Different strategies are implemented to introduce anti-SARS-CoV-2 therapeutics. These approaches are repurposing US FDA-approved nucleot(s)ide analogues like lamivudine to inhibit SARS-CoV-2 nsp12 [19] or non-viral drugs like clofazimine to inhibit viral shedding [20] and using traditional herbal medicine or medical plant extracts [21–23], and drug discovery of virtually available libraries of small molecules [24,25]. SARS-CoV-2 undergoes high rates of nucleotide mutations, and it may influence the therapeutic potential of nucleot(s)ide analogues.

We recently published an *in silico* work introducing a potential small molecule, Compound 38, interfering with the virus attachment and fusion, and experimental experiments are undergoing on Compound 38 [26]. Due to the probability of the emergence of drug-resistance strains of SARS-CoV-2, targeting more conserved targets of SARS-CoV-2, link nsp12, is more rational. This approach could be implemented in the in-silico process of drug discovery and experimental works. Following the previous study [26], it was aimed to highlight a second potent inhibitor of SARS-CoV-2 to block the nsp12 protein. Targeting nsp12 is much more promising to avoid the emergence of evolved resistance mutants, and it also improves the chance of inhibiting the virus replication by suppressing the viral replication complex. The novelties of the present study are using a very large database of drug-like small molecules, finding druggable cavity pocket(s) from crystallographic nsp12 structure, establishing several pharmacophores in one druggable cavity to increase the chance of drug-discovery, two-step validations at the molecular docking and MDS, and estimation of the effect of known nsp12 variants on the affinity of the lead compound(s). The results highlighted a promising lead compound, ZINC03977803, with stable binding to the nsp12 and less affected by the already known nsp12 mutants.

## Materials & methods

### Preparation of SARS-CoV-2 spike glycoprotein

The crystallographic structure of human SARS-CoV-2 nsp12 was obtained from the Protein Data Bank (PDB) [27]. The PDB was searched for the crystallographic structure of the full-length nsp12 protein. Two structures with PDB IDs 7BTF and 6M71 with resolutions 2.90 Å and 2.95 Å were provided for the study [28]. PDB files of the structure were curated by using UCSF Chimera v1.10.2. Accordingly, only chain(s) containing nsp12 amino acid residues were cleaned from other non-standard or other protein chains. To achieve better resolution, the structures were energy minimized and checked for amino acid atoms using Swiss PDB viewer (SPDBV) v4.1.0, as reported before [29,30].

### Finding druggable cavity pockets of SARS-CoV-2 RdRp

CavityPlus server was used for precise and robust protein cavity detection and functional analysis of the energy minimized SARS-CoV-2 nsp12 3D structure [31]. The server's module Cavity was used to detect potential binding sites on the surface of the nsp12 protein. The binding sites were ranked with ‘Druggability’ and ‘DrugScore’. The strongest cavity with the highest DrugScore was further analyzed by the server's modules ChemPharmer, CorrSite and CovCys.

ChemPharmer was used to extract pharmacophore features within the selected cavity through the receptor-based pharmacophore modeling program, Pocket. Potential allosteric ligand binding sites were identified by the CorrSite module based on motion correlation analysis between allosteric and orthosteric cavities (cut-off Z-score of 0.5). CovCys was used to automatically detect druggable cysteine residues for covalent ligand design, which is especially useful for identifying novel binding sites for covalent allosteric ligand design. The server setting was kept as default, as we have reported before.

### Optimization of pharmacophore features & hit identification

ChemPharmer may result in several pharmacophore features. Features with relatively close coordinates were manually selected to make different pharmacophores covering the surface cavity of the druggable binding site(s). The features were reduced to H-bond acceptor (HBA) centers, H-bond donor (HBD) centers, and hydrophobic centers (HC). The number of each feature was further reduced in each pharmacophore to increase the chance of hit identification. Hit identification was performed by importing the .pdb files containing the pharmacophore features into the ZINCPharmer server [32]. The files were double-checked by the server, indicating pharmacophore feature classes are as same as the .pdb files.

ZINCPharmer contains eight databases of chemical compounds and small molecules, including ZINC Purchasable (206,433,075 conformers of 21,777,093 compounds), ZINK Purchasable Thiols (321,022 conformers of 39,257 compounds), Alex Doemling UDC (946,136 conformers of 95,075 compounds), ZINC Drug Database (20,432 conformers of 2770 compounds), ZINC In Man (77,269 conformers of 10,813 compounds), ZINC Drug Database of Metabolites (32,339 conformers of 5030 compounds), ZINC Natural Derivatives (185,393 conformers of 25,164 compounds) and ZINC Natural Products (1,374,813 conformers of 172,324 compounds). ZINC database used for virtual screening is comprised of drug-like or lead-like commercially available small molecules that are easy to handle for computer-aided drug discovery approaches [33].

Each database was applied to every pharmacophore. For pharmacophores with the least features that might lead to high numbers of hits, the server settings were changed to molecular weight ≤500 da, rotatable bonds ≤10, max root mean square deviation (RMSD) = 500, and max total hit = 500. Open Bable command line was used to check the duplication of identified hits for each pharmacophore [34].

### Protein sequence retrieval & finding amino acid substitutions

Literature was searched for the amino acid substitution at the SARS-CoV-2 nsp12 protein till 20 April 2021. UCSF Chimera v1.10.2 was used for making computationally mutant constructs. Accordingly, the protein was structurally edited using Dunbrack's rotamer [35] to choose the most probable rotamer(s) for the substituted amino acid residue(s). The computationally constructed mutants were checked for clashes with adjustment atom residues. The clashes were curated by energy minimization by Swiss-PBD view v4.1.0 [29].

### Molecular docking & hit optimization

The affinity of identified hits was further investigated by using molecular docking. For this purpose, the open-source tool Autodock Vina [36] was used in the setting of PaDEL-ADV (http://www.yapcwsoft.com/dd/padeladv/), as we reported before [26]. Vina is a fast and accurate tool for ligand-receptor docking, and with PaDEL-ADV, it allows high-throughput screenings of several ligands in one run. Molecular docking was performed on the druggable binding site with the highest DrugScore. In this regard, the viral nsp12 was treated as the receptor in the MGLTools 1.5.6 software (Molecular Graphics Laboratory, The Scripps Research Institute). Accordingly, a grid-box was defined in 3D dimension to encompass the entire cavity.

The hit(s) with the highest affinity to the nsp12 protein were selected for further analysis. Accordingly, the binding site(s) of the chosen hits were highlighted and compared with the viral nsp7 and nsp8 proteins in the crystallographic structure of the viral proteins (PDB ID: 7BTF) by using UCSF Chimera v1.10.2.

### Validation of molecular docking

The molecular docking of the identified lead compound was further validated by generating a set of 50 decoys using the directory of useful decoys, enhanced (DUD-E) [37]. DUD-E is useful to provide a set of property-matched decoys from ZINC based on the SMILES file of the lead compound. Furthermore, five different crystallographic structures of SARS-CoV-2 nsp12 were retrieved from PDB database with following IDs: 6M71 [28], 6YYT [38], 7BTF [28], 7C2K [39] and 7ED5 [40]. The structures were <3Å resolutions and cleaned from non-standard residues and non-nsp12 chains. The same grid box configuration was used for molecular docking with Autodock Vina (as stated above) with no changes in the size or coordination. In this regard, the affinity of the identified lead compound was challenged with fifty different decoys against the same target from different crystallographic structures (except 7BTF, which was used for the prediction of druggable cavities).

### ADMET profiling

The toxicity estimation software tool (TEST) v4.2.1 was used to predict the toxicity of the lead compounds. The highest prediction accuracy and improved applicable domain were obtained by the tool's consensus method. The validation of each predicted toxicity was evaluated using external statistical validation of training- and test sets within the software package. Six different predictive tests were evaluated for each compound, including 96-h fathead minnow LC50, 48-h *Daphnia magna* LC50, 40-h *Tetrahymena pyriformis* IGC50, Oral rat LD50, developmental toxicity, and ames mutagenicity.

### Molecular dynamic simulation

The simulation of nsp12 in the presence and absence of the lead compound was performed using the GROMACS version 2021.2 [41]. The CHARMM36 force field was applied to the nsp12 [42]. The same procedure was applied to the lead compound for the MDS of the complexed docked structure. Accordingly, the lead compound topology was generated by using the CGenFF server [43–46]. The explicit compound coordination was matched in the topology file by sorting the list of bonds using a Perl script provided by the GROMACS team (sort_mol2_bonds.pl). All starting structures were solvated in a simple cubic water box under periodic boundary conditions using a 1.0 nm distance from the protein to the box faces. The system was neutralized by nine Na^+^ ions. In this regard, both systems, nsp12 alone or in complex with the lead compound were neutralized by 9 NA^+^ molecules. Following steepest descent energy minimization, the systems were equilibrated under a constant number of particles, volume, and temperature (NVT) conditions for 60 ps at 300°K, followed by 60 ps under constant particle pressure and temperature (NPT) conditions. Finally, 10 ns MD was produced to analyze the stability of each system. Lenard Jones and Coulomb Potentials (kJ/mol) were estimated to analyze the lead compound interaction with the nsp12. MDS was run on a system utilizing a brand of Intel(R) Core(TM) i3-4160 CPU @ 3.60GHz accompanied by a GPU brand of AMD Radeon RX 5500 XT 8G OC GDDR6. Different Gromacs modules, including hbond, energy, sasa, covar, and anaeig were used for the analysis of MDS data of nsp12 and the lead compound [47,48].

## Results

The crystallographic structure of nsp12 with PDB ID: 6M71 was discarded for the study due to missing amino acid sidechain atoms. These amino acids were including, Phe70, Lys73, Arg74, Glu83, Lys98, Phe101, Phe102, Ile114, Arg365 and Asp824. Therefore, screening for the druggable cavities was proceeded by the other crystallographic structure of SARS-CoV-2 nsp12 structure with PDB ID: 7BTF. Total energy was minimized to -31860.766 kJ.mol^-1^. The energy-minimized nsp12 structure was used for druggable cavity detection.

**Figure 1.**
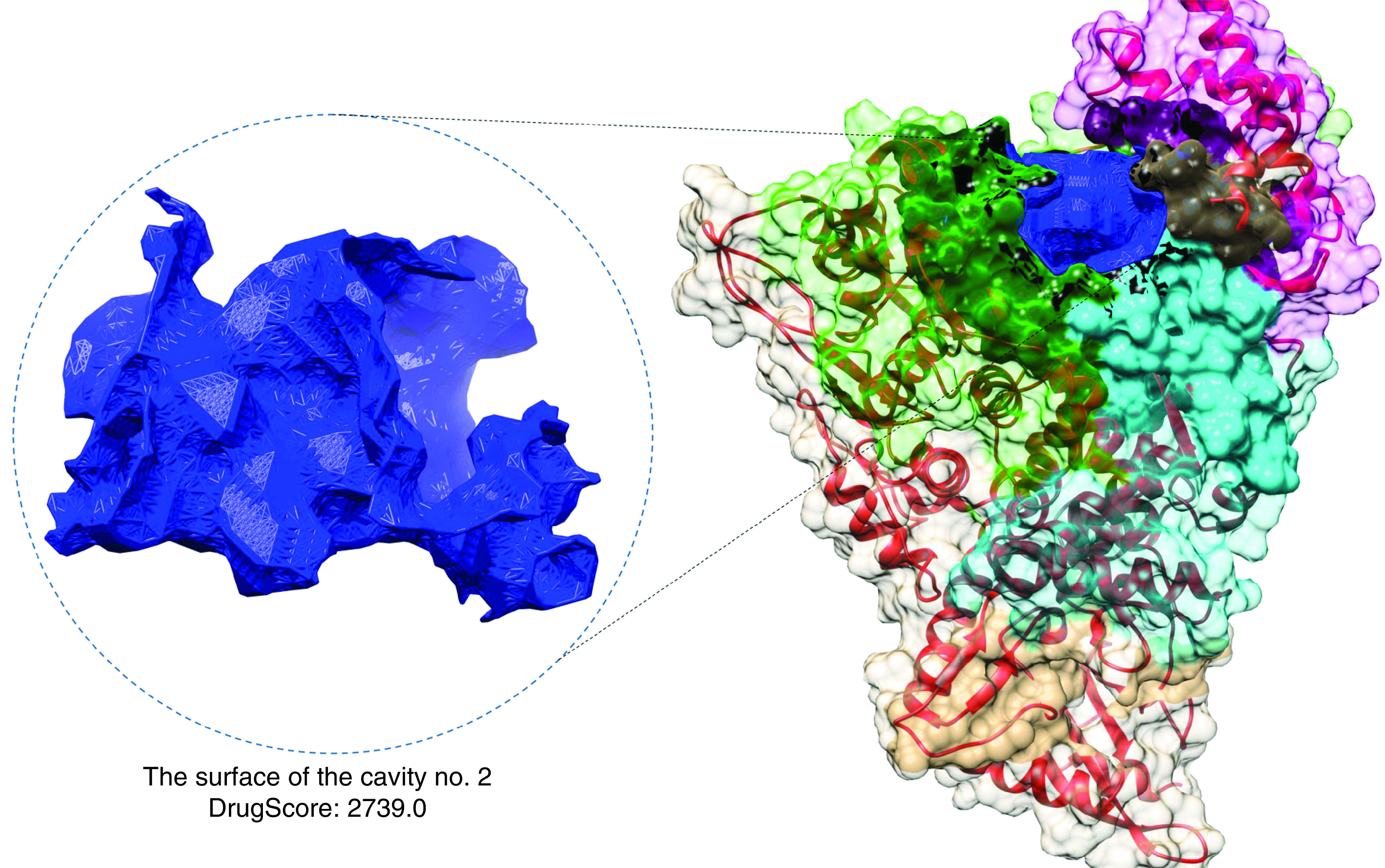
Cavity no. 2 and its surface within the SARS-CoV-2 protein. The nsp12 fingers are presented in Green color. Palm and thumb domains are also illustrated in cyan and magenta colors, respectively. As it is illustrated, cavity no. 2 (colored in black) is located adjustment to the left-handed finger (residues 366–581), palm (residues 582–620 and 680–815) and thumb (residues 816–920) domains.

The detection of potential cavities within the nsp12 protein showed four strong druggable binding sites (Figure 1). Furthermore, six cavities binding sites with minimum possibility of being druggable with minimum and maximum DrugScore 161.0 and 22.0 were highlighted. Twenty-two other binding site cavities had a weak possibility of being druggable. The only cavity no. 2 with the highest DrugScore (23739.0) was selected for pharmacophore modeling. As shown in Figure 1, the druggable cavity pocket resides in the catalytic site of the nsp12 protein, where fingers, palm, and thumb meet each other.

**Figure 2.**
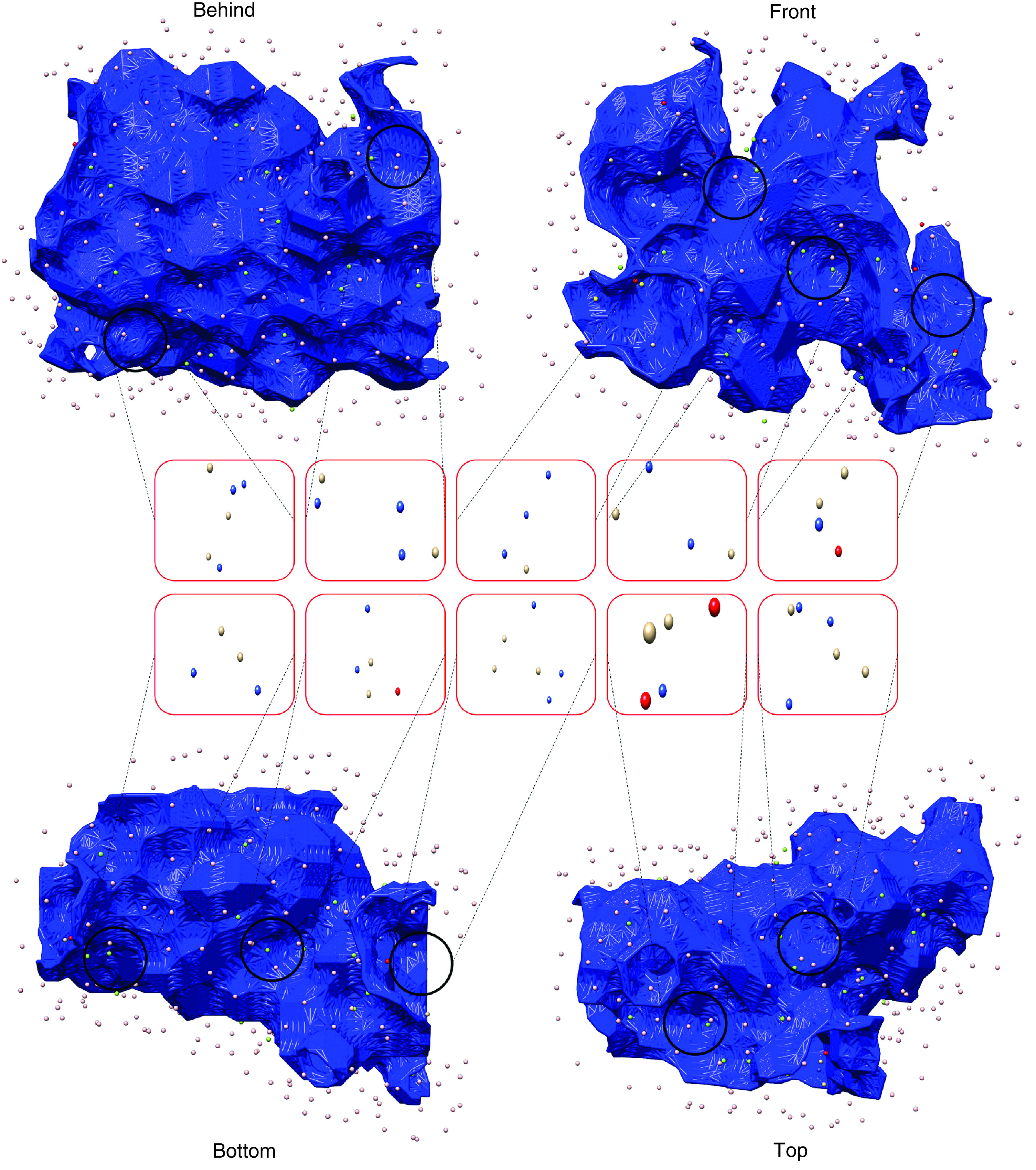
Schematic representation of adjunct pharmacophore features distributed around the cavity surface no.2. The figure demonstrates the distribution of the pharmacophore features around the surface of cavity no. 2. H-bond acceptor (HBA) centers are shown in red, H-bond donor (HBD) centers are in blue, H-bond root is in cyan, and hydrophobic centers (HC) are in green, negative electrostatic center is in yellow, and positive electrostatic center is in gray. Pharmacophore features are shown in spears. The coordinates of HBA centers are overlapping with HBDs; therefore, they are not visible.

Pharmacophore class of cavity no. 2 had several pharmacophore features including, 21 HBA centers, 20 HBD centers, 42 H-bond roots, eighteen HC, 12 negative electrostatic centers (NEC), and five positive electrostatic centers (PEC). As shown in Figure 2, the neighboring pharmacophore features surrounding the cavity no. 2 surface were manually selected. For simplicity of Pharmacophore class of cavity no. 2 had several phaselection of neighboring pharmacophore features, H-bond roots, PEC and NEC were ignored. The pharmacophore features and their coordinates are provided in the (Supplementary Table 1). Ten pharmacophores were adopted based on the neighboring pharmacophore features, each containing 5 to 9 features (Figure 2). The largest pharmacophore was comprised of three HBD centers, three HBA centers, and three hydrophobic centers. CorrSite results demonstrated no allosteric site within cavity no. 2. However, cavities 3, 8, 7, 6, 12 and 9 contained allosteric sites with Z-scores of 2.68, 2.5, 1.6, 1.23, 1.05 and 0.77, respectively. CovCys results also demonstrated that Cys622 within the SARS-CoV-2 nsp12 has a 92% possibility of being a covalent targetable cysteine residue, which had a significant ligandablility potential (pKdAvg = 6.74).

The virtual high-throughput screening (HTS) of the ZINCPharmer databases resulted in 3932 hits from the ten different pharmacophores. Table 1 shows the unique number of identified hits from HTS. After duplication removal, 1,274 unique hits remained to be docked with the SARS-CoV-2 nsp12.

**Table 1.** The results of unique identified hits obtained by HTS of the pharmacophores through ZINCPharmer database.

Pharmacophore	ZINCPharmer database								Duplications^‡^	Unique total
	ZINC purchasable	ZINK purchasable thiol	Alex Doemling UDC	ZINC drug database	ZINC In man	ZINC drug database of metabolite	ZINC natural derivative	ZINC natural product		
Pharma1	1	0	0	0	0	0	0	0	0	1
Pharma2	13	0	0	0	0	0	2	2	4	13
Pharma3	2	0	0	11	0	0	0	0	9	4
Pharma4	34	0	9	1	19	3	19	5	33	57
Pharma5	210	0	0	0	0	0	0	3	130	83
Pharma6^†^	500	51	2	271	500	74	500	500	1,906	492
Pharma7	212	0	0	4	8	1	3	50	146	132
Pharma8	24	0	0	0	0	0	0	13	27	10
Pharma9^†^	500	121	9	6	12	0	43	182	396	477
Pharma10	6	0	0	0	2	0	0	5	8	5
Total	1,502	172	11	286	541	77	567	760	2,659	1,274

† For pharmacophores no. 6 and 9 ZINCPharmer search limits, including molecular weight ≤500 da, rotatable bonds ≤10, max RMSD = 500 and max total hit = 500 were applied.

‡ Duplication was automatically selected by Open Babel command line based on the same strings and conformers.

**Figure 3.**
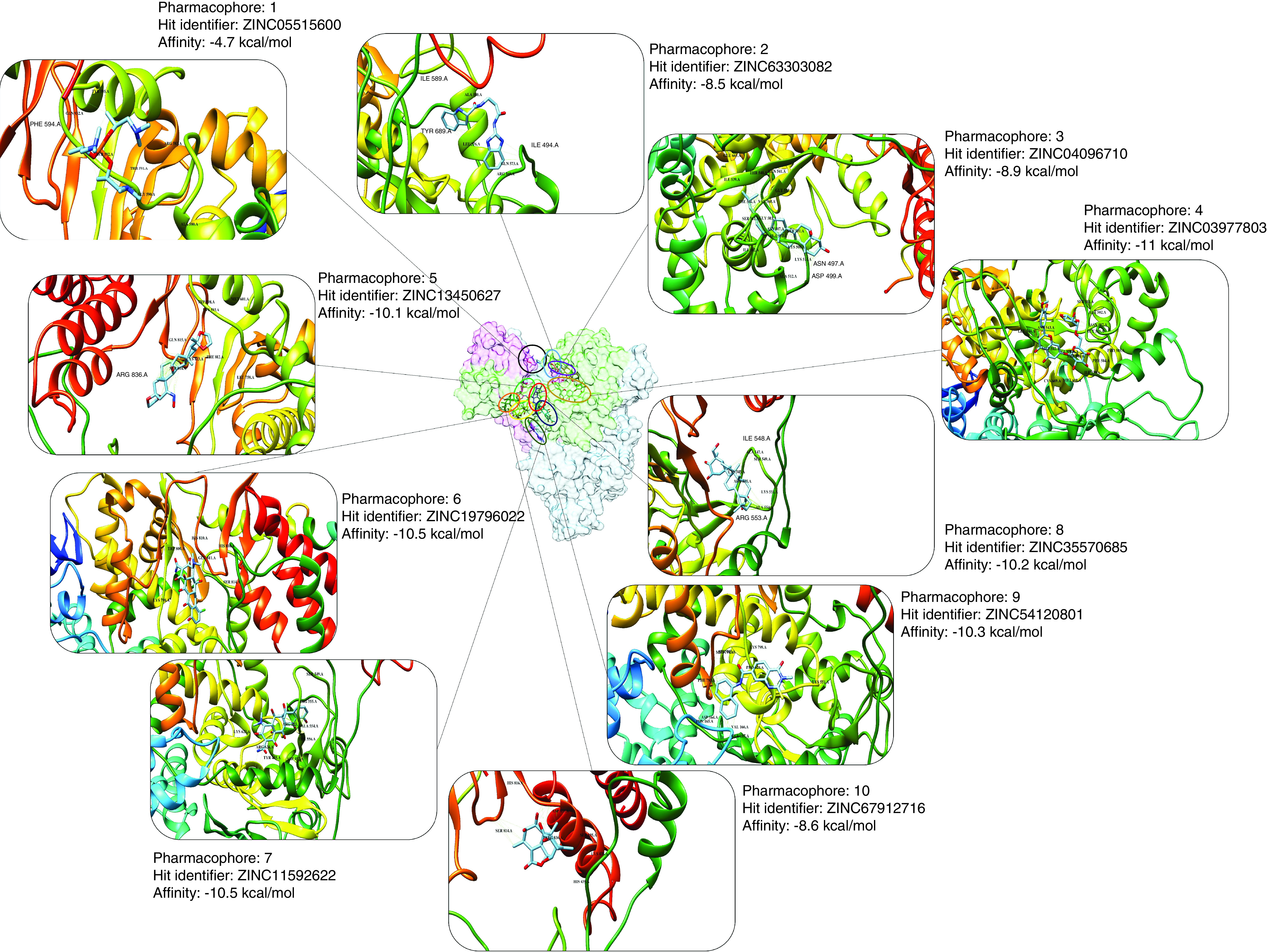
Schematic of highlighted hits from different pharmacophores close or within the finger domain of the nsp12 protein. The exact site of binding of the selected hits is presented with different colors on the surface of the nsp12 at the center of the figure. Further information, including pharmacophore number and their relative hit's affinity, is also presented. In this regard, the hit identified for pharmacophore no. 1 had the lowest affinity to its nsp12, and the hit highlighted for pharmacophore no. 4 had the highest affinity to nsp12 protein.

**Figure 4.**
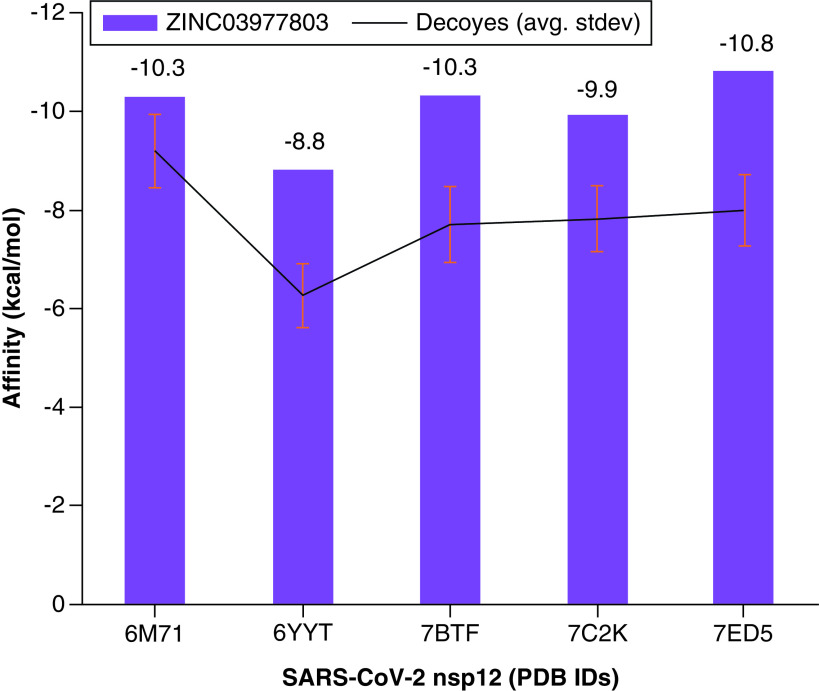
Molecular docking validation. The lead compound showed high affinity to the different crystallographic structures of SARS-CoV-2 nsp12. The lead compound had an affinity of -10.3 kcal.mol^-1^ to 7BTF and it was slightly lower than -11 kcal.mol^-1^ due to flexibility and non-redundancy of autodock Vina. PDB: Protein Data Bank.

For molecular docking of the identified hits, a gridbox was defined by having the center coordinates of 125.14, 126.874 and 147.48 along the x-, y- and z-axis, respectively. The gridpoints' size was 76, 88 and 58 along the x-, y- and z-axis, respectively. The spacing was kept to default values. The docking results of the hits within the predicted druggable cavity pocket were interesting. The hits obtained from virtual screening resulted in a lead (ZINC03977803) from the pharmacophore number four with the affinity of -11 kcal.mol^-1^ (Figure 3). Analyzing the interaction of ZINC03977803 to the nsp12 showed the involvement of amino acid residues proximate to the finger motifs of the viral protein. The contact between amino acid residues of the viral protein with the hits was investigated by VDM overlap ≥0.4 angstroms. The amino acids were including, Ser501, Ala502, Gly503, Phe504, Pro505, Asn507, Ile539, Thr540, Gln541, Met542, Asn543, Leu544, Met668 and Cys669. The results of interactive residues within nsp12 and other lead compounds of other pharmacophores are provided in the (Supplementary Table 2). Additionally, only one compound was found due to screening of the chemical library through the pharmacophore number 1. The compound had been discarded because of the lowest interaction energy (-4.7 kcal.mol^-1^) with the nsp12 (Supplementary Table 3). The predicted toxicity (Table 1) of the lead compounds showed the lowest toxicity of ZINC03977803. Ames mutagenicity was positive for ZINC04096710 and ZINC54120801 (Table 2). The affinity of ZINC04096710 to the SARS-CoV-2 nsp12 was further validated by a set of property-matched decoys. As shown in Figure 4, the affinity of the lead compound was higher than the affinity mean of decoys. Accordingly, the decoys' affinities to nsp12 in 6M71, 6YYT, 7BTF, 7C2K and 7ED5 crystallographic structures were -9.2 ± 0.75 (min: -9.4; max: -6.7), -6.262 ± 0.65 (min: -8.2; max: -4.9), -7.71 ± 0.78 (min: -9.1; max: -5.9), -7.82 ± 0.66 (min: -9.2; max: -6.4) and -7.98 ± 0.72 (min: -10.3; max: -5.9).

**Table 2.** The predicted toxicity of the lead compounds by using TEST.

Lead	Pharmacophore no.^†^	Formula	Fathead minnow LC_50_ (96 h) (mg/l)	Daphnia magna LC_50_ (48 hr) (mg/l)	T. pyriformis IGC_50_ (40 h) (mg/l)	Oral rat LD_50_ (mg/kg)	Developmental toxicity	Ames mutagenicity
ZINC63303082	2	C19H21N5O2	0.14	12.25	N/A	1732.74	0.90	Negative
ZINC04096710	3	C27H48O3	4.31	3.89	4.43	194.50	0.75	Positive
ZINC03977803	4	C28H32O15	1.12E-03	340.43	N/A	2972.04	0.52	Negative
ZINC13450627	5	C25H41NO6	N/A	N/A	N/A	207.69	0.79	Negative
ZINC19796022	6	C22H21N2O8Cl	N/A	26.14	N/A	2402.53	1.00	Negative
ZINC11592622	7	C22H24N2O9	1.12	4.29	N/A	1165.80	0.88	Negative
ZINC35570685	8	C30H48O5	1.06	1.92	N/A	491.96	0.91	Negative
ZINC54120801	9	C20H15N3O2S	0.15	6.17	N/A	2028.77	0.84	Positive
ZINC67912716	10	C19H28O7	1.51	21.36	N/A	155.24	0.71	Negative

† Pharmacophore No.1 was discarded due to its low affinity to the nsp12.

ZINC03977803 was chosen for continuing the study. The literature was searched for reported amino acid substitutions of SARS-CoV-2's nsp12 worldwide. As a result, forty-five nsp12 mutants were created (Table 3). The lead compound had the lowest affinity to mutant N489K (-9.6 kcal.mol^-1^). Additionally, the affinity of the compound to the mutants S6L, T141I, P328S, A526V and T806I was -11.8 kcal.mol^-1^, -11.5 kcal.mol^-1^, -11.3 kcal.mol^-1^, -11.8 kcal.mol^-1^ and -11.5 kcal.mol^-1^.

**Table 3.** The nsp12 amino acid substitutions and their impact on the binding affinity of ZINC03977803 to the mutant nsp12.

Mutant^†^	Position	Affinity (kcal.mol^-1^
A/V	97	-11.0
P/L	323	-10.8
P/L	314	-10.5
S/L	6	-11.8
G/Y	25	-11.0
T/I	26	-10.7
G/V	44	-10.0
Y/C	80	-11.0
T/I	85	-10.6
K/R	91	-10.8
K/R	103	-10.6
M/V	110	-10.4
T/I	141	-11.5
D/G	154	-10.3
D/V	161	-10.8
G/S	179	-11.0
G/C	228	-10.2
S/N	229	-10.8
K/N	263	-10.9
T/M	276	-10.7
P/S	328	-11.3
A/V	382	-10.5
A/S	400	-10.6
V/I	405	-10.6
V/F	405	-10.5
A/V	406	-10.8
A/V	443	-11.0
A/V	449	-10.5
I/V	466	-10.5
N/K	489	-9.6
A/V	526	-11.8
R/H	640	-10.6
A/V	656	-10.7
M/I	668	-10.5
A/S	699	-10.3
N/T	734	-10.6
G/S	774	-10.5
D/Y	804	-10.9
T/I	806	-11.5
K/R	807	-10.7
H/L	810	-10.2
G/S	823	-10.8
H/Y	872	-11.0
D/Y	879	-10.9
W/C	916	-10.7

† The mutation are extracted from literature [42–45].

**Figure 5.**
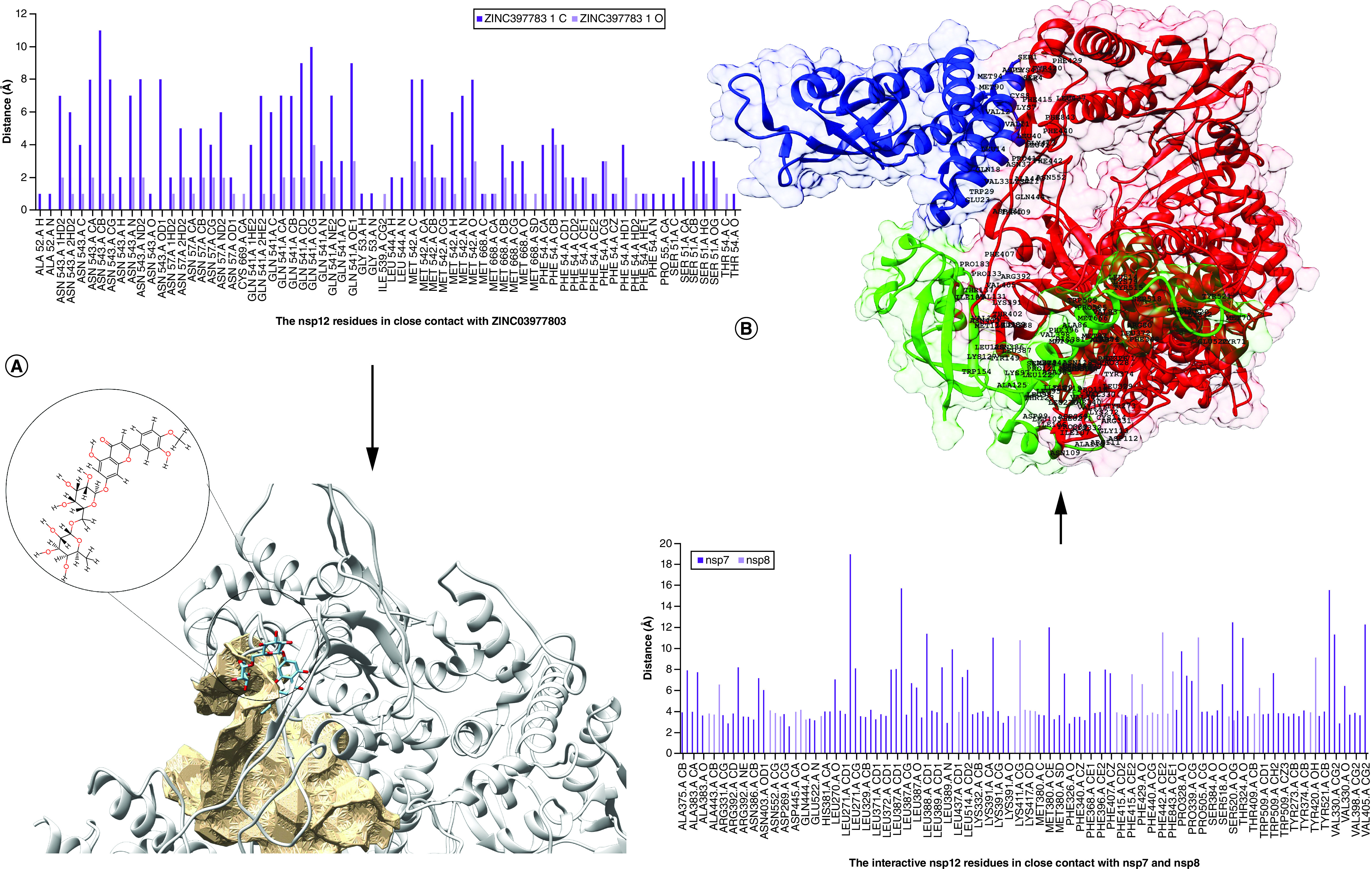
The binding site of ZINC03977803 within the nsp12 cavity pocket and interaction between nsp7/8 with nsp12. **(A)** Shows the contacts of atom residues O and C with hydrogen donor and acceptor sites of the given amino acid residues within nsp12. Further 2D view and rotation of ZINC03977803 around cavity no. 2 are presented on the bottom-left side of the figure. **(B)** Right-top of the figure represents the nsp7 (green) and nsp8 (blue) interactive residues with nsp12 (red) in the crystallographic data of the SARS-CoV-2 proteins (PDB ID: 7BTF). Data showed higher numbers of nsp7 residues in close contact with nsp12. Further information on the interactive nsp12 residues is presented at the bottom-right of the figure.

The residues of nsp12 with close contacts to the hit compound were Ser501, Phe504, Pro505, Asn507, Ile539, Gln541, Met542, Asn543, Leu544, Met668 and Cys669. The mean distance between the O and C atoms of ZINC03977803 in contact with nsp12 residues was 2.46 ± 0.73 Å (Figure 5). As shown in Figure 5B, Pro505 in the nsp12 protein was in close contact with ZINC03977803 and was also resided in the neighboring site of interaction between nsp12 and nsp7/8 proteins.

**Figure 6.**
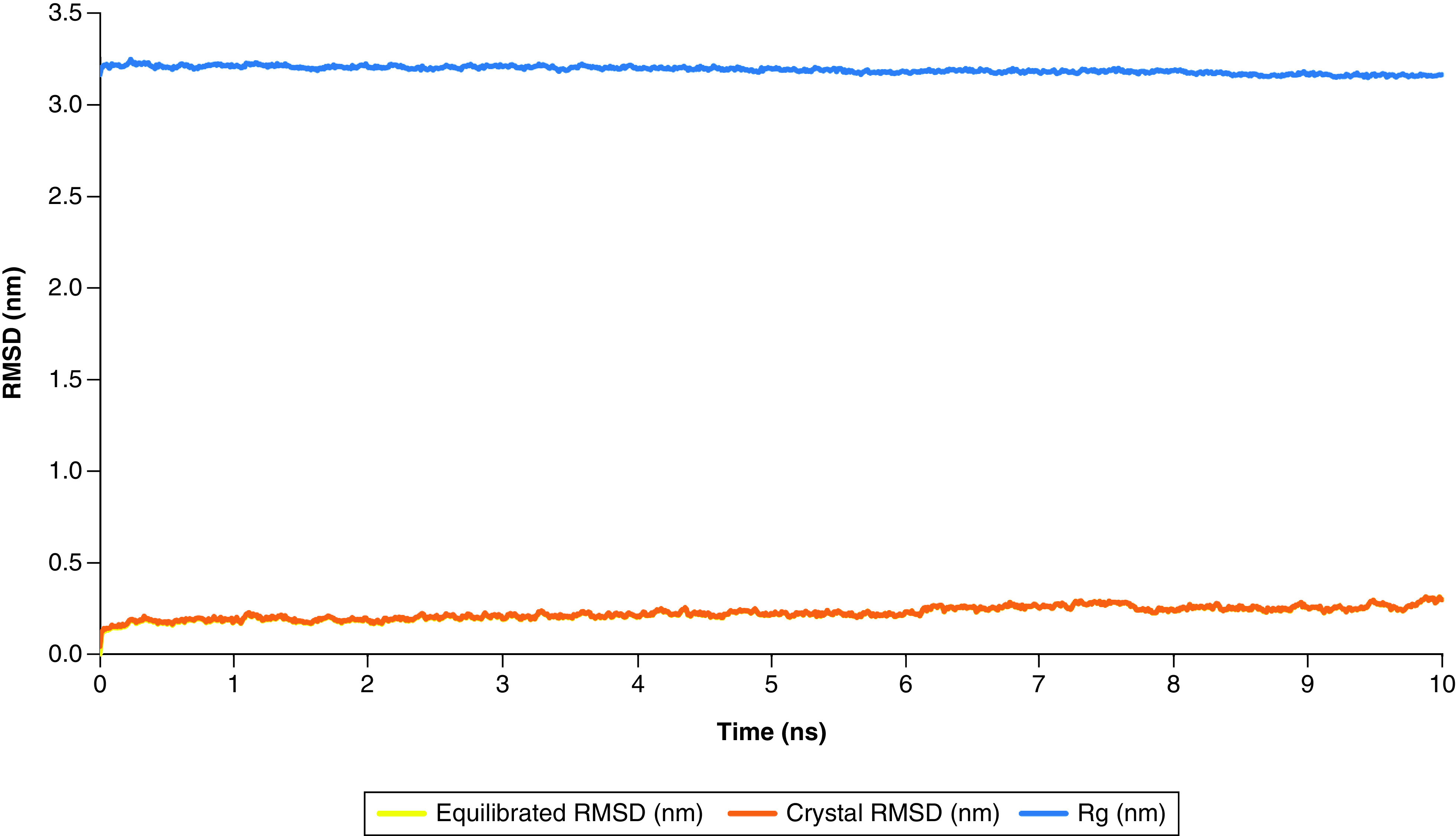
Root mean square of deviation and radius of gyration at nm scale within 10 ns of molecular dynamic production. It has been shown that nsp12 RMSD is relative to the crystal structure. RMSD fluctuation differences between the equilibrated protein and the crystal protein are slight indications that the structure is stable favorably. Rg of nsp12 protein showed slight fluctuation along with simulation time, suggesting proper folding of the protein. Rg: Radius of gyration; RMSD: Root mean square deviation.

MDS was performed in 10 ns to evaluate the conformational changes of nsp12 protein in complex with the lead compound, ZINC03977803. RMSD and radius of gyration (Rg) measures were evaluated for speculating the stability and proper folding of the protein (Figure 6). The only significant fluctuation of RMSD and Rg was observed at the beginning of the simulation due to the system's energy minimization and imperfect position restraints. GROMACS rms, energy, hbond and mindist utility modules were used to evaluate the stability of the lead compound within the cavity, the total interaction energy between the lead compound and nsp12, hydrogen bonding, and distance between groups of the lead and protein backbone during 10 ns of MDS.

**Figure 7.**
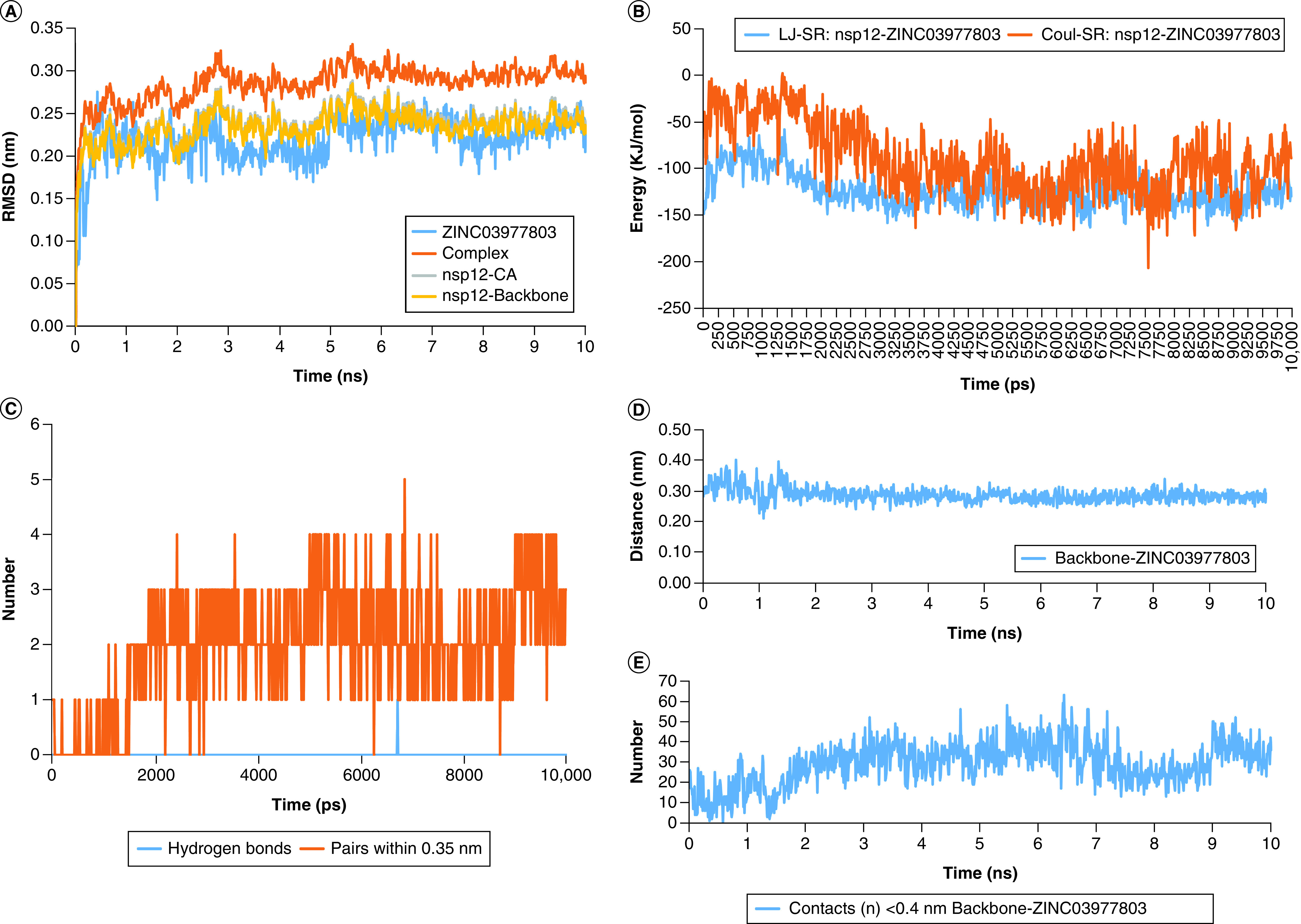
The graphs of RMS, energy, hbond and mindist modules of GROMACS for ZINC03977803 and nsp12 backbone. **(A)** RMSD fluctuation of the lead compound ZINC03977803 and its complex with nsp12 protein. The difference in the complex structure was substantially higher than that observed in the nsp12-CA and nsp12-Backbone. **(B)** Plot of the short-range potentials of LJ and Coul shows lower electrostatic and higher inter intermolecular interactions at the first 2ns of molecular dynamic simulation (MDS), which is converged after 2ns of MDS. **(C)** H-bonding between nsp12 amino acid residues and the lead compounds showed the N-terminal residues, Ala52, was the hydrogen bond donor. **(D)** Demonstrates the distance between nsp12-backbone interactive residues and the lead compound. **(E)** Shows the number of interactive residues of nsp12-backbone in close contact (<0.4 nm) with the lead compound during 10 ns of MDS. Coul-SR: Short-range Coulomb; LJ-SR: Short-range Lennard-Jones; RMSD: Root mean square deviation.

As is shown in Figure 7A, RMSD fluctuation of the lead compound was according to the nsp12's, suggesting stable binding of ZINC03977803 lead compound within the binding cavity pocket of nsp12. The interaction energy of ZINC03977803 to the nsp12 was measured by short-range (SR) Lennard-Jones (LJ-SR) and Coulomb (Coul-SR) potentials. The Sum of LJ-SR and Coul-SR was reported as total interaction energy (Figure 7B). Accordingly, the mean of LJ-SR and Coul-SR were -123.53 ± 17.94 kJ/mol and -90.38 ± 37.03 kJ/mol. Total interaction energy was estimated as -213.92 ± 27.49 kJ/mol.

Hbond showed H19 of ZINC03977803 as the donor site was involved in Ala406N and Gly671N residues on the nsp12. The distance between H19 of the lead compound with amine groups of Ala406 and Gly671 was 3.1625 ± 0.2 Å. The hydrogen bond pairing during 10 ns of MDS is shown in Figure 7C. Distance and number were also measured between the lead compound ZINC03977803 and the nsp12 backbone (Figure 7D & E). The results showed a mean distance of 2.86 ± 0.2 Å and 29.12 ± 10.57 number of contacts <4 Å between ZINC03977803 and the nsp12 backbone. These results suggest stable close contacts between ZINC03977803 and nsp12 during 10 ns of MDS.

**Figure 8.**
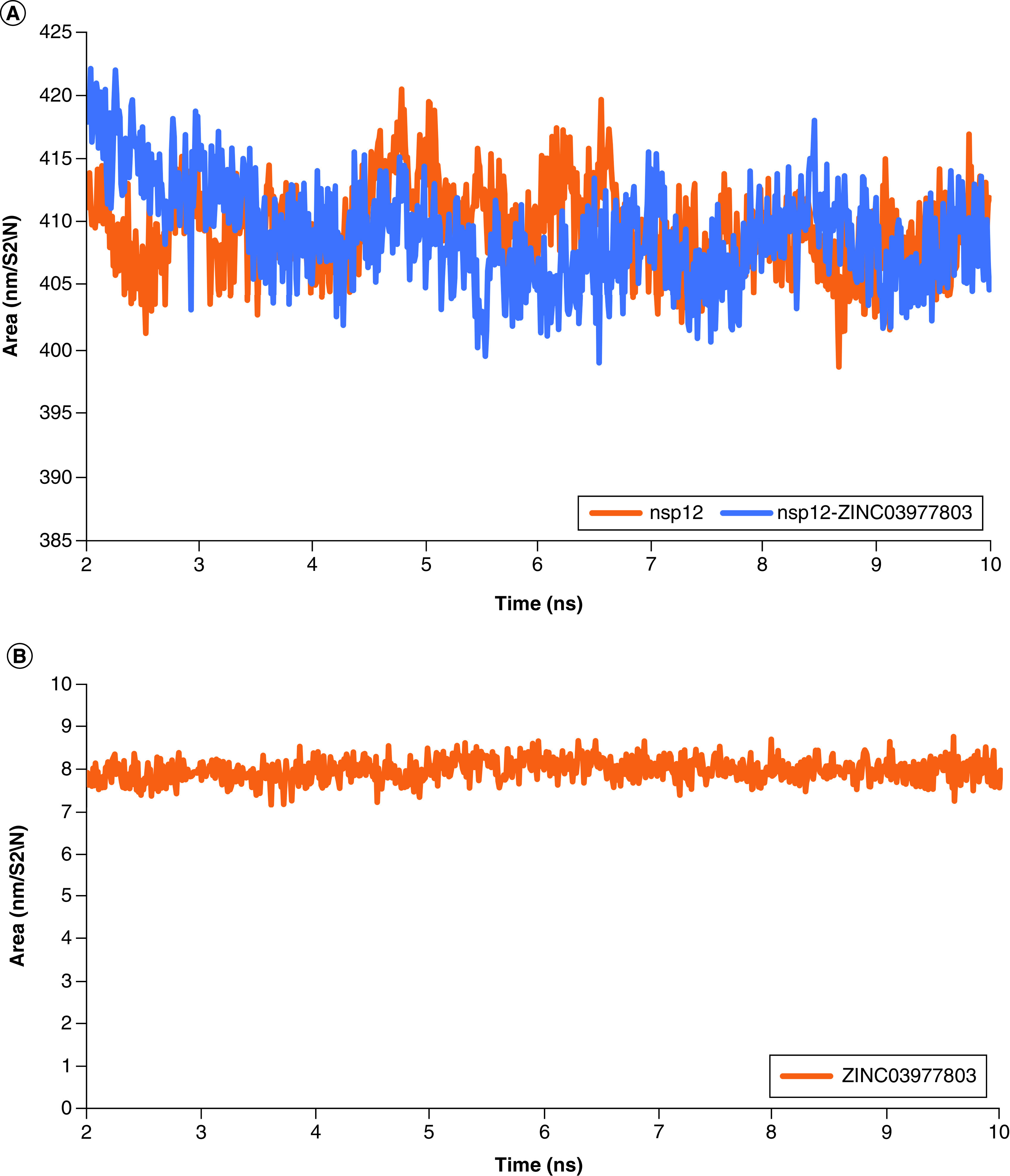
Solvent accessible surface areas analysis of nsp12, nsp12–ligand complex and the lead compound. **(A)** Demonstrates stable solvent-accessible surface area fluctuations during 8ns of the molecular dynamic simulated system. **(B)** Demonstrates stable solvent-accessible surface area of the lead compound during the molecular dynamic simulation. Only a slight reduction of surface area was observed in the complex of nsp12 with the lead compound, suggesting the location of the small molecule inside the cavity pocket.

As shown in Figure 8A, solvent-accessible surface areas (SASA) were evaluated to confirm the stability of the complex after the convergence time (2ns to 10ns). Accordingly, no substantial changes were observed in the nsp12's SASA alone (409.64 ± 3.34 nm/S^2^/N) or its complex with the lead compound (409.12 ± 3.98 nm/S^2^/N). This is an implication of stable binding of the lead compound in its binding site within the nsp12 cavity pocket. As shown in Figure 8B, the SASA of the lead compound was 7.99 ± 0.26, and it was stable during the MDS. This was accompanied by a slight reduction in the SASA of the nsp12–ligand complex (-0.52 ± 0.64 nm/S^2^/N). This reduction in the average area is due to the molecular weight of the lead compound.

**Figure 9.**
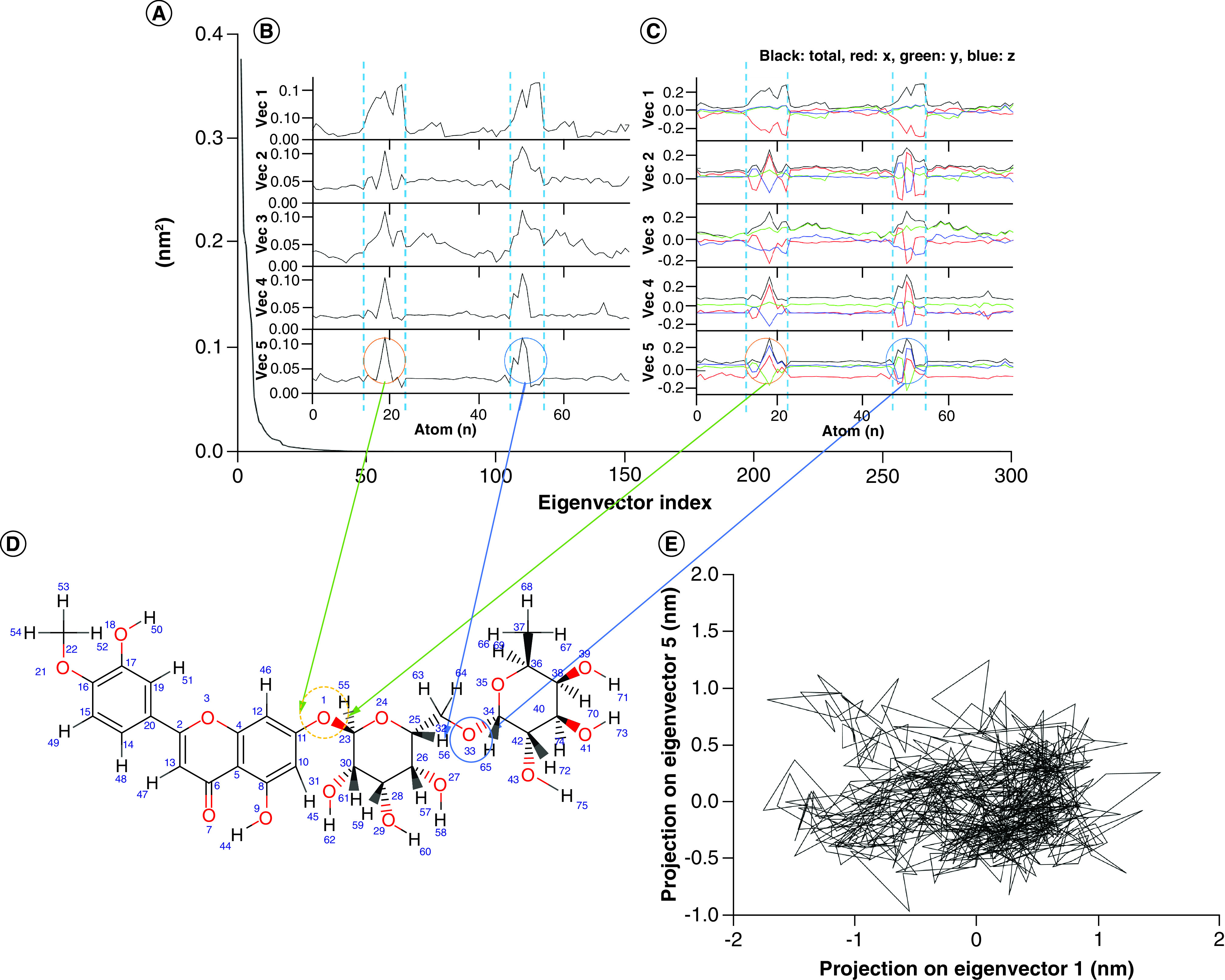
Covariance matrix (215 × 215) and PCA analysis of ZINC03977803 and SARS-CoV-2 nsp12. **(A)** Eigenvector indexes indicate 100% eigenvalues of the covariance matrix in the 1st five eigenvector indexes. **(B & C)** Shows root mean square fluctuation and eigenvector components of the first five vectors. The result indicated atom motions at numbers 12 to 22 and 48 to 55, representing; **(C)** Aromatic ring rotations around the C-O-C bonds within the nsp12 protein. **(D)** Illustrates the 2D projection of nsp12 Cα atoms motion on eigenvectors 1 (-1.7 nm to 1.5 nm) and 5 (-0.9 nm to 1.5 nm).

In addition, covariance matrix and principal component analysis (PCA) of the lead compound in complex with nsp12 was assessed on the Cα, where the system was converged (2 ns to 10 ns). As shown in Figure 9, only the first five eigenvectors containing 100% of data variance were selected for PCA analysis. The RMS fluctuation and eigenvector component (Figure 9B & C) of the lead compound with 75 atoms were stable, and it was consistent with the RMSD (Figure 6). However, fluctuations were observed at two points, indicating rotation of the ligand aromatic rings around two C-O-C bonds. This may also improve the pose of the lead within the nsp12 binding pocket. Accordingly, consistent with the high RMSD changes of nsp12 in complex with ZINC03977803, atom motion of 2D projection was observed in the nsp12 Cα (n = 915) complex with ZINC03977803.

## Discussion

There is an excellent opportunity to use natural products and plant-derived small antiviral molecules to deal with challenging viral diseases [49,50]. In this regard, *in silico* approaches have the advantage of using different virtual chemical libraries for drug discovery against SARS-CoV-2 by using fewer resources [51]. Accordingly, some studies demonstrated the potential of plant-derived natural products in suppressing the SARS-CoV-2 RdRp [52–55]. In the present study, a rational drug discovery process was implemented to find the possible druggable cavities within SARS-CoV-2 nsp12, the catalytic subunit of the RdRp complex. As a result, ten druggable sites were screened to find hits from an extensive chemical library of natural compounds and small molecules in the ZINC database. Only one lead compound, ZINC03977803, was highlighted to stably inhibit the formation of RdRp owing to dramatic conformational changes. The affinity of the lead compound was challenged with a set of property-matched decoy compounds. In addition, the MDS demonstrated stable binding of ZINC03977803 within nsp12 cavity pocked.

The substitutions at the amino acid residues of the nsp12 protein may affect the affinity of drugs or even confer drug resistance. Therefore, the affinity of the lead compound to different mutant constructs of nsp12 was measured. The results demonstrated that the residue N489 is very important in the druggable cavity of nsp12 since its substitution to N489K resulted in decreased affinity of the compound to nsp12. The substitution was only reported in a genome-wide associated study in a sample from Iceland [56]. The rest of the mutants did not substantially affect the binding affinity of the lead compound to the nsp12, suggesting a conserved unique druggable cavity for drug discovery of SARS-CoV-2 nsp12. As a finding, amine groups of Ala406 and Gly671 at the close contact in the binding site of the lead compound were the hydrogen bond donors. The hydrogen bindings were also stable during MDS. This suggests a stable, strong contact between the lead compound and nsp12, which makes it a potent inhibitor of SARS-CoV-2 replication and transcription (-213.92 ± 27.49 kJ/mol).

A further important finding was the presence of the residue Pro505 at the binding site of the ZINC03977803, where the nsp12 make the multimeric RdRp complex with heterodimer nsp7/nsp8 subunits. This would highlight the possible role of the lead compound, ZINC03977803, in suppressing the formation of the SARS-CoV-2 RdRp complex. This is also supported by the stable close RMSD fluctuation of the lead compound alongside the nsp12, significant RMSD changes, and reduced nsp12 SASA while they are in a complex.

The introduced lead compound, ZINC03977803, had no known biological activity in the ChEMBL or ZINC database. In the future, we aim to investigate the anti-SARS-CoV-2 activity of the highlighted lead compound. Recently, we have reported Compound 38 as a possible inhibitor of SARS-CoV-2 attachment and fusion. Due to the high rate of amino acid substitution of SARS-CoV-2, dual therapy by targeting two viral proteins would be promising in the future. Therefore, we highlighted ZINC03977803 as the second potent anti-SARS-CoV-2 compound.

Furthermore, the process implemented for the presented study can be utilized for further drug discovery of other SARS-CoV-2 main exoribonuclease proteins nsp14 and nsp12. This might enhance the efficiency of nucleot(s)ide analogues in the treatment of COVID-19 infection. In the present study, eight potent nsp12 inhibitors were highlighted to bind to the different druggable pharmacophore cavities within the nsp12 protein. The role of each of those compounds could also be investigated in other studies.

## Conclusion

The lead compound ZINC03977803 showed stable interaction with higher potential and hydrogen bonding with the catalytic subunit of SARS-CoV-2, nsp12. The implemented mechanisms by which the lead compound would inhibit nsp12 activity could directly interfere with the interaction of nsp12 with nsp7/nsp8, inhibiting the whole enzyme completion. It may also induce suppression of the proofreading activity of nsp12.

**Summary points**

- SARS-CoV-2 nsp12 is a conserved region and a suitable target for drug discovery.
- The SARS-CoV-2 was searched for druggable cavity pockets.
- The best druggable cavity was chosen for pharmacophore-based drug discovery and virtual screening of chemical libraries.
- ZINC03977803 was highlighted as a potential candidate targeting SARS-CoV-2 nsp12.
- Molecular dynamic simulation demonstrated stable binding of ZINC03977803 and conformational changes in nsp12.
