# Supplementary material for: Digging for the discovery of SARS-CoV-2 nsp12 inhibitors: a pharmacophore-based and molecular dynamics simulation study
Source: Future Virol. 2022 Aug 8:10.2217/fvl-2022-0054. doi: 10.2217/fvl-2022-0054 (PMC9370102; doi:10.2217/fvl-2022-0054)
Supplement: Supplementary file 1 [file supplementary-table-1.pdf]

Table S1. The manually curated features of pharmacophore number 4 extracted from the SARS-CoV-2 nsp12 protein.

```
HEADER    User defined
COMPND    Pharmacophore features
AUTHOR    Generated by Pocket
REMARK    Excluded Volume radius:3.0A
REMARK    1
REMARK    1 Creation time Fri May  7 02:32:32 2021
REMARK    1
REMARK    2 N represent h-bond donor center
REMARK    2 O represent h-bond acceptor center
REMARK    2 C represent hydrophobic center
REMARK    3 F represent root of h-bond root
REMARK    4 H represent positive electrostatic center
REMARK    4 S represent negative electrostatic center
REMARK    5 B represent excluded Volume center
HETATM   10   N  POK     2     129.500 117.000 142.000  0.500
HETATM   20   N  POK     2     127.000 119.500 144.000  0.500
HETATM   30   C  POK     2     132.250 116.440 141.200  1.000
HETATM   40   C  POK     2     125.060 118.250 143.450  1.000
HETATM   50   O  POK     2     129.500 117.000 142.000  0.500
HETATM   60   O  POK     2     127.000 119.500 144.000  0.500
END
```
