# Supplementary material for: Digging for the discovery of SARS-CoV-2 nsp12 inhibitors: a pharmacophore-based and molecular dynamics simulation study
Source: Future Virol. 2022 Aug 8:10.2217/fvl-2022-0054. doi: 10.2217/fvl-2022-0054 (PMC9370102; doi:10.2217/fvl-2022-0054)
Supplement: Supplementary file 2 [file supplementary-table-2.pdf]

Table S2. The interactive overlapping residues of the SARS-CoV-2 nsp12 protein with the lead compound, ZINC03977803.

Pharmacophore 1:

Allowed overlap: -0.4

H-bond overlap reduction: 0

Ignore contacts between atoms separated by 5 bonds or less

Detect intra-residue contacts: True

Detect intra-molecule contacts: True

168 contacts

**atom1**	**atom2**	**overlap**	**distance**
#1 UNL 1 C	#0 TYR 595.A CZ	2.799	0.781
#1 UNL 1 P	#0 SER 592.A OG	2.699	0.901
#1 UNL 1 C	#0 TYR 595.A OH	2.609	0.771
#1 UNL 1 P	#0 SER 592.A CB	2.598	1.382
#1 UNL 1 C	#0 TYR 595.A HH	2.463	0.417
#1 UNL 1 C	#0 TYR 595.A OH	2.362	1.018
#1 UNL 1 C	#0 ARG 583.A NH2	2.167	1.338
#1 UNL 1 C	#0 ARG 583.A 2HH2	1.928	0.952
#1 UNL 1 C	#0 TYR 595.A CE2	1.928	1.652
#1 UNL 1 C	#0 SER 592.A CA	1.811	1.949
#1 UNL 1 C	#0 GLN 932.A NE2	1.777	1.728
#1 UNL 1 C	#0 TYR 595.A OH	1.775	1.605
#1 UNL 1 C	#0 GLY 590.A O	1.646	1.714
#1 UNL 1 C	#0 TYR 595.A CZ	1.637	1.943
#1 UNL 1 C	#0 TYR 595.A CE1	1.603	1.977
#1 UNL 1 P	#0 SER 592.A CA	1.537	2.443

#1 UNL 1 C #0 SER 592.A N 1.509 1.996

#1 UNL 1 C #0 ARG 583.A CZ 1.452 2.128

#1 UNL 1 C #0 GLN 932.A 2HE2 1.434 1.446

#1 UNL 1 C #0 TYR 595.A HH 1.411 1.469

#1 UNL 1 P #0 SER 592.A HG 1.351 1.749

#1 UNL 1 C #0 SER 592.A CB 1.318 2.442

#1 UNL 1 O #0 TYR 595.A HH 1.308 1.152

#1 UNL 1 O #0 SER 592.A OG 1.272 1.688

#1 UNL 1 C #0 GLY 590.A C 1.249 2.331

#1 UNL 1 C #0 GLY 590.A O 1.229 2.131

#1 UNL 1 C #0 GLN 932.A 1HE2 1.214 1.666

#1 UNL 1 O #0 SER 592.A H 1.179 1.281

#1 UNL 1 O #0 SER 592.A CB 1.106 2.234

#1 UNL 1 N #0 GLY 590.A O 1.100 2.020

#1 UNL 1 P #0 SER 592.A N 1.075 2.650

#1 UNL 1 C #0 SER 592.A N 1.073 2.432

#1 UNL 1 C #0 SER 592.A OG 1.071 2.309

#1 UNL 1 C #0 SER 592.A CB 1.019 2.741

#1 UNL 1 N #0 GLN 932.A 2HE2 1.013 1.627

#1 UNL 1 C #0 GLN 932.A 2HE2 0.999 1.881

#1 UNL 1 C #0 TYR 595.A CZ 0.995 2.585

#1 UNL 1 C #0 TYR 595.A HH 0.986 1.894

#1 UNL 1 C #0 ARG 583.A NE 0.979 2.526

#1 UNL 1 O #0 SER 592.A N 0.958 2.127

#1 UNL 1 O #0 SER 592.A CB 0.951 2.389

#1 UNL 1 C #0 SER 592.A CB 0.950 2.810

#1 UNL 1 C #0 THR 591.A C 0.935 2.645

#1 UNL 1 C #0 TYR 595.A CE2 0.931 2.649

#1 UNL 1 C #0 SER 592.A HG 0.927 1.953

#1 UNL 1 P #0 SER 592.A H 0.924 2.176

#1 UNL 1 C #0 SER 592.A CA 0.910 2.850

#1 UNL 1 C #0 SER 592.A HG 0.898 1.982

#1 UNL 1 O #0 TYR 595.A OH 0.887 2.073

#1 UNL 1 C #0 SER 592.A CB 0.882 2.878

#1 UNL 1 P #0 TYR 595.A HH 0.876 2.224

#1 UNL 1 C #0 SER 592.A H 0.870 2.010

#1 UNL 1 C #0 ARG 583.A 1HH2 0.832 2.048

#1 UNL 1 C #0 SER 592.A OG 0.830 2.550

#1 UNL 1 N #0 GLN 932.A NE2 0.769 2.496

#1 UNL 1 C #0 GLY 590.A CA 0.747 3.013

#1 UNL 1 C #0 GLN 932.A NE2 0.735 2.770

#1 UNL 1 C #0 GLN 932.A 1HE2 0.728 2.152

#1 UNL 1 C #0 TYR 595.A CD2 0.728 2.852

#1 UNL 1 C #0 TYR 595.A HE2 0.703 2.177

#1 UNL 1 O #0 SER 592.A OG 0.676 2.284

#1 UNL 1 C #0 GLN 932.A NE2 0.672 2.833

#1 UNL 1 O #0 SER 592.A CA 0.623 2.717

#1 UNL 1 O #0 SER 592.A OG 0.616 2.344

#1 UNL 1 C #0 SER 592.A H 0.612 2.268

#1 UNL 1 C #0 GLN 932.A 2HE2 0.591 2.289

#1 UNL 1 C #0 GLN 932.A CD   0.583 2.997

#1 UNL 1 C #0 GLY 590.A C   0.553 3.027

#1 UNL 1 C #0 TYR 595.A CD1   0.546 3.034

#1 UNL 1 C #0 GLY 590.A CA   0.531 3.229

#1 UNL 1 C #0 SER 592.A OG   0.528 2.852

#1 UNL 1 C #0 ARG 583.A HE   0.522 2.358

#1 UNL 1 C #0 GLN 932.A NE2   0.496 3.009

#1 UNL 1 C #0 GLN 932.A CG   0.479 3.281

#1 UNL 1 C #0 TYR 595.A CE1   0.472 3.108

#1 UNL 1 C #0 PHE 594.A HD2   0.471 2.409

#1 UNL 1 C #0 GLN 932.A 1HE2 0.459 2.421

#1 UNL 1 P #0 TYR 595.A HE2   0.446 2.654

#1 UNL 1 O #0 TYR 595.A CZ   0.439 2.721

#1 UNL 1 O #0 TYR 595.A CE2   0.438 2.722

#1 UNL 1 N #0 ARG 583.A NH2   0.436 2.829

#1 UNL 1 O #0 SER 592.A CB   0.429 2.911

#1 UNL 1 P #0 TYR 595.A OH   0.406 3.194

#1 UNL 1 C #0 GLY 590.A O   0.396 2.964

#1 UNL 1 O #0 SER 592.A HG   0.392 2.068

#1 UNL 1 P #0 SER 592.A C   0.390 3.410

#1 UNL 1 N #0 ARG 583.A 2HH2 0.377 2.263

#1 UNL 1 C #0 THR 591.A N   0.372 3.133

#1 UNL 1 P #0 TYR 595.A CE2   0.358 3.442

#1 UNL 1 O #0 TYR 595.A HE2   0.308 2.152

#1 UNL 1 C #0 TYR 595.A CE1   0.298 3.282

#1	UNL	1	C	#0	THR	591.A	C	0.295	3.285
#1	UNL	1	C	#0	TYR	595.A	HE1	0.291	2.589
#1	UNL	1	C	#0	THR	591.A	O	0.282	3.078
#1	UNL	1	C	#0	SER	592.A	N	0.271	3.234
#1	UNL	1	C	#0	PHE	594.A	CD2	0.268	3.312
#1	UNL	1	N	#0	TYR	595.A	OH	0.258	2.882
#1	UNL	1	N	#0	GLY	590.A	C	0.254	3.086
#1	UNL	1	C	#0	TYR	595.A	HE2	0.243	2.637
#1	UNL	1	C	#0	ARG	583.A	NH1	0.243	3.262
#1	UNL	1	C	#0	THR	591.A	CA	0.207	3.553
#1	UNL	1	C	#0	TYR	595.A	CG	0.206	3.374
#1	UNL	1	C	#0	GLN	932.A	NE2	0.184	3.321
#1	UNL	1	C	#0	ARG	583.A	1HH2	0.166	2.714
#1	UNL	1	C	#0	SER	592.A	C	0.166	3.414
#1	UNL	1	C	#0	GLN	932.A	2HE2	0.123	2.757
#1	UNL	1	C	#0	SER	592.A	H	0.122	2.758
#1	UNL	1	C	#0	GLN	932.A	CB	0.121	3.639
#1	UNL	1	C	#0	SER	592.A	CB	0.120	3.640
#1	UNL	1	C	#0	THR	591.A	CA	0.101	3.659
#1	UNL	1	C	#0	GLY	590.A	C	0.086	3.494
#1	UNL	1	C	#0	ALA	580.A	O	0.073	3.287
#1	UNL	1	C	#0	SER	592.A	OG	0.056	3.324
#1	UNL	1	C	#0	GLN	932.A	CD	0.046	3.534
#1	UNL	1	C	#0	SER	592.A	OG	0.045	3.335
#1	UNL	1	P	#0	TYR	595.A	CZ	0.043	3.757

#1 UNL 1 C #0 TYR 595.A CE2 0.034 3.546

#1 UNL 1 C #0 SER 592.A O 0.028 3.332

#1 UNL 1 N #0 ARG 583.A 1HH2 0.018 2.622

#1 UNL 1 C #0 TYR 595.A HE2 0.010 2.870

#1 UNL 1 C #0 TYR 595.A OH -0.007 3.387

#1 UNL 1 C #0 THR 591.A CA -0.034 3.794

#1 UNL 1 C #0 THR 591.A C -0.036 3.616

#1 UNL 1 N #0 ARG 583.A NH2 -0.036 3.301

#1 UNL 1 C #0 ARG 583.A 2HH2 -0.052 2.932

#1 UNL 1 C #0 GLN 932.A CG -0.074 3.834

#1 UNL 1 O #0 THR 591.A C -0.084 3.244

#1 UNL 1 C #0 SER 592.A OG -0.096 3.476

#1 UNL 1 C #0 SER 592.A CB -0.112 3.872

#1 UNL 1 C #0 ARG 583.A 2HH2 -0.118 2.998

#1 UNL 1 C #0 ARG 583.A NH2 -0.131 3.636

#1 UNL 1 N #0 TYR 595.A HH -0.132 2.772

#1 UNL 1 P #0 SER 592.A O -0.138 3.718

#1 UNL 1 C #0 PHE 594.A O -0.140 3.500

#1 UNL 1 C #0 ARG 583.A NH2 -0.142 3.647

#1 UNL 1 O #0 ARG 583.A 2HH2 -0.145 2.605

#1 UNL 1 C #0 GLY 590.A O -0.149 3.509

#1 UNL 1 C #0 ARG 583.A NH2 -0.153 3.658

#1 UNL 1 N #0 GLY 590.A CA -0.169 3.689

#1 UNL 1 P #0 THR 591.A C -0.171 3.971

#1 UNL 1 C #0 ARG 583.A CD -0.173 3.933

#1 UNL 1 N #0 GLN 932.A 1HE2 -0.184 2.824

#1 UNL 1 C #0 SER 592.A N -0.194 3.699

#1 UNL 1 N #0 ARG 583.A CZ -0.200 3.540

#1 UNL 1 C #0 SER 592.A H -0.228 3.108

#1 UNL 1 C #0 THR 591.A N -0.244 3.749

#1 UNL 1 C #0 ARG 583.A NH2 -0.253 3.758

#1 UNL 1 C #0 GLY 590.A C -0.257 3.837

#1 UNL 1 O #0 ARG 583.A NH2 -0.259 3.344

#1 UNL 1 O #0 SER 592.A CA -0.268 3.608

#1 UNL 1 C #0 ARG 583.A HE -0.279 3.159

#1 UNL 1 O #0 SER 592.A HG -0.286 2.746

#1 UNL 1 C #0 TYR 595.A HH -0.287 3.167

#1 UNL 1 C #0 TYR 595.A HE1 -0.290 3.170

#1 UNL 1 N #0 GLN 932.A CD -0.297 3.637

#1 UNL 1 O #0 GLY 590.A O -0.303 3.243

#1 UNL 1 O #0 THR 591.A CA -0.313 3.653

#1 UNL 1 C #0 SER 592.A CA -0.318 4.078

#1 UNL 1 C #0 SER 592.A CA -0.321 4.081

#1 UNL 1 C #0 ARG 583.A HE -0.339 3.219

#1 UNL 1 C #0 THR 591.A N -0.340 3.845

#1 UNL 1 C #0 TYR 595.A CE2 -0.357 3.937

#1 UNL 1 C #0 ARG 583.A 1HH2 -0.367 3.247

#1 UNL 1 C #0 GLN 932.A 1HE2 -0.368 3.248

#1 UNL 1 C #0 SER 592.A OG -0.371 3.751

#1 UNL 1 C #0 ARG 583.A NH2 -0.375 3.880

#1 UNL 1 N #0 ARG 583.A NE   -0.380 3.645

#1 UNL 1 C #0 ARG 583.A NE   -0.388 3.893

Pharmacophore 2:

160 contacts

atom1 atom2 overlap distance

#1 UNL 1 C #0 ARG 569.A NH1   2.770 0.615

#1 UNL 1 C #0 ARG 569.A CZ   2.656 0.804

#1 UNL 1 C #0 ARG 569.A NH2   2.636 0.749

#1 UNL 1 C #0 TYR 689.A CE1   2.542 0.918

#1 UNL 1 C #0 ARG 569.A 1HH2 2.465 0.295

#1 UNL 1 C #0 TYR 689.A OH   2.292 0.818

#1 UNL 1 C #0 ARG 569.A NH2   2.203 1.182

#1 UNL 1 C #0 TYR 689.A CZ   2.162 1.298

#1 UNL 1 C #0 ARG 569.A NH1   2.138 1.247

#1 UNL 1 C #0 TYR 689.A OH   2.105 1.155

#1 UNL 1 C #0 TYR 689.A CZ   2.045 1.415

#1 UNL 1 O #0 ALA 580.A CB   2.018 1.282

#1 UNL 1 C #0 ILE 589.A CG2   1.980 1.660

#1 UNL 1 C #0 ARG 569.A 1HH1 1.944 0.816

#1 UNL 1 C #0 TYR 689.A CE1   1.928 1.532

#1 UNL 1 C #0 ARG 569.A 1HH1 1.870 0.890

#1 UNL 1 C #0 TYR 689.A HE1   1.778 0.982

#1 UNL 1 C #0 ARG 569.A CZ   1.692 1.768

#1 UNL 1 C #0 ARG 569.A NH1 1.679 1.706

#1 UNL 1 C #0 ARG 569.A CZ 1.679 1.781

#1 UNL 1 C #0 TYR 689.A HE1 1.657 1.103

#1 UNL 1 C #0 TYR 689.A HH 1.621 0.989

#1 UNL 1 C #0 ARG 569.A 2HH1 1.606 1.154

#1 UNL 1 C #0 TYR 689.A OH 1.577 1.533

#1 UNL 1 C #0 TYR 689.A OH 1.468 1.792

#1 UNL 1 C #0 ILE 589.A CB 1.466 2.174

#1 UNL 1 C #0 TYR 689.A CZ 1.437 2.023

#1 UNL 1 C #0 ARG 569.A 1HH2 1.373 1.237

#1 UNL 1 C #0 ARG 569.A NE 1.360 2.025

#1 UNL 1 C #0 TYR 689.A HH 1.359 1.521

#1 UNL 1 C #0 TYR 689.A CE1 1.355 2.105

#1 UNL 1 C #0 TYR 689.A OH 1.311 2.069

#1 UNL 1 C #0 ALA 580.A CB 1.280 2.210

#1 UNL 1 C #0 TYR 689.A CD1 1.214 2.246

#1 UNL 1 C #0 ARG 569.A 2HH2 1.185 1.575

#1 UNL 1 C #0 TYR 689.A CZ 1.181 2.129

#1 UNL 1 C #0 ARG 569.A 1HH2 1.148 1.612

#1 UNL 1 C #0 ALA 580.A CB 1.147 2.613

#1 UNL 1 C #0 ARG 569.A 1HH1 1.139 1.621

#1 UNL 1 C #0 TYR 689.A OH 1.105 2.155

#1 UNL 1 C #0 ARG 569.A NH1 1.082 2.303

#1 UNL 1 C #0 ALA 580.A CB 1.070 2.690

#1 UNL 1 C #0 TYR 689.A OH 1.067 2.193

#1 UNL 1 C #0 ARG 569.A NH2 1.066 2.169

#1 UNL 1 C #0 ILE 589.A CG2 1.047 2.593

#1 UNL 1 C #0 ILE 589.A CB 1.021 2.619

#1 UNL 1 C #0 ARG 569.A 1HH1 1.016 1.594

#1 UNL 1 C #0 TYR 689.A CE2 0.984 2.476

#1 UNL 1 C #0 TYR 689.A HH 0.968 1.792

#1 UNL 1 C #0 TYR 689.A CE2 0.862 2.598

#1 UNL 1 C #0 ILE 589.A CG2 0.857 2.633

#1 UNL 1 C #0 TYR 689.A CZ 0.837 2.623

#1 UNL 1 C #0 ARG 569.A NH2 0.825 2.560

#1 UNL 1 C #0 TYR 689.A CD1 0.748 2.712

#1 UNL 1 O #0 ALA 580.A CA 0.747 2.553

#1 UNL 1 C #0 TYR 689.A HH 0.743 1.867

#1 UNL 1 C #0 TYR 689.A CE1 0.729 2.731

#1 UNL 1 C #0 ALA 580.A CB 0.710 2.780

#1 UNL 1 C #0 GLN 573.A CG 0.706 2.934

#1 UNL 1 C #0 ARG 569.A NH1 0.680 2.555

#1 UNL 1 C #0 ARG 569.A 2HH2 0.677 2.083

#1 UNL 1 N #0 ALA 580.A CB 0.657 2.863

#1 UNL 1 C #0 ARG 569.A 2HH1 0.653 2.107

#1 UNL 1 C #0 ARG 569.A 2HH1 0.652 2.108

#1 UNL 1 C #0 TYR 689.A CZ 0.649 2.661

#1 UNL 1 C #0 ARG 569.A NE 0.627 2.758

#1 UNL 1 C #0 ALA 580.A CB 0.595 2.895

#1 UNL 1 C #0 LEU 576.A O 0.586 2.654

#1 UNL 1 C #0 ARG 569.A CZ 0.586 2.874

#1 UNL 1 C #0 ARG 569.A 1HH1 0.574 2.186

#1 UNL 1 C #0 ILE 589.A CD 0.561 3.079

#1 UNL 1 C #0 ARG 569.A CD 0.556 3.084

#1 UNL 1 N #0 TYR 689.A OH 0.527 2.613

#1 UNL 1 C #0 ILE 579.A CG2 0.508 3.132

#1 UNL 1 C #0 ARG 569.A 1HH1 0.480 2.130

#1 UNL 1 C #0 TYR 689.A HE1 0.466 2.294

#1 UNL 1 C #0 ARG 569.A NH1 0.456 2.779

#1 UNL 1 C #0 ARG 569.A CZ 0.456 2.854

#1 UNL 1 N #0 ILE 589.A CG2 0.440 3.080

#1 UNL 1 N #0 ARG 569.A 1HH2 0.439 2.201

#1 UNL 1 C #0 ARG 569.A CD 0.432 3.208

#1 UNL 1 C #0 ARG 569.A NE 0.425 2.960

#1 UNL 1 C #0 TYR 689.A OH 0.424 2.956

#1 UNL 1 C #0 TYR 689.A HH 0.422 2.458

#1 UNL 1 C #0 LYS 577.A CE 0.420 3.220

#1 UNL 1 C #0 ILE 579.A CB 0.408 3.232

#1 UNL 1 C #0 ILE 589.A CA 0.400 3.240

#1 UNL 1 C #0 TYR 689.A HE1 0.363 2.397

#1 UNL 1 C #0 ILE 579.A CG2 0.356 3.284

#1 UNL 1 C #0 TYR 689.A CG 0.290 3.170

#1 UNL 1 C #0 TYR 689.A CZ 0.283 3.297

#1 UNL 1 C #0 ILE 589.A CG1 0.277 3.363

#1 UNL 1 C #0 TYR 689.A CE2 0.271 3.039

#1 UNL 1 C #0 ILE 589.A CG1 0.265 3.375

#1 UNL 1 C #0 ILE 494.A O 0.264 2.976

#1 UNL 1 C #0 ILE 589.A C 0.215 3.245

#1 UNL 1 C #0 TYR 689.A CE1 0.213 3.097

#1 UNL 1 C #0 TYR 689.A CE2 0.185 3.275

#1 UNL 1 C #0 TYR 689.A CD1 0.157 3.303

#1 UNL 1 C #0 TYR 689.A CD2 0.156 3.304

#1 UNL 1 C #0 ARG 569.A 1HH2 0.153 2.457

#1 UNL 1 O #0 ALA 580.A C 0.135 2.985

#1 UNL 1 C #0 ARG 569.A HE 0.131 2.629

#1 UNL 1 C #0 GLN 573.A CG 0.130 3.510

#1 UNL 1 N #0 TYR 689.A HH 0.120 2.520

#1 UNL 1 C #0 ARG 569.A 1HH2 0.112 2.648

#1 UNL 1 C #0 GLN 573.A CD 0.099 3.361

#1 UNL 1 N #0 ARG 569.A NH2 0.096 3.169

#1 UNL 1 C #0 ILE 589.A CD 0.088 3.552

#1 UNL 1 C #0 LEU 576.A CA 0.088 3.552

#1 UNL 1 C #0 ARG 569.A NH2 0.077 3.158

#1 UNL 1 C #0 ARG 569.A NH2 0.062 3.323

#1 UNL 1 C #0 TYR 689.A HH 0.052 2.708

#1 UNL 1 C #0 LEU 576.A C 0.043 3.417

#1 UNL 1 C #0 ARG 569.A CZ 0.034 3.276

#1 UNL 1 C #0 LEU 576.A O 0.032 3.328

#1 UNL 1 N #0 ALA 580.A CB 0.013 3.507

#1 UNL 1 N #0 GLY 590.A CA -0.009 3.529

#1 UNL 1 C #0 ALA 580.A CB -0.020 3.660

#1 UNL 1 C #0 LEU 576.A O -0.027 3.117

#1 UNL 1 C #0 ILE 589.A CB -0.031 3.521

#1 UNL 1 C #0 TYR 689.A CE1 -0.049 3.359

#1 UNL 1 C #0 ALA 580.A CA -0.064 3.554

#1 UNL 1 C #0 TYR 689.A CD2 -0.094 3.554

#1 UNL 1 C #0 LEU 576.A CB -0.102 3.742

#1 UNL 1 C #0 TYR 689.A HH -0.114 2.874

#1 UNL 1 C #0 ILE 579.A CB -0.119 3.759

#1 UNL 1 C #0 ALA 580.A CA -0.135 3.625

#1 UNL 1 C #0 ALA 580.A CA -0.137 3.627

#1 UNL 1 C #0 ALA 580.A CA -0.143 3.903

#1 UNL 1 C #0 GLN 573.A CG -0.159 3.799

#1 UNL 1 C #0 TYR 689.A HE2 -0.181 2.941

#1 UNL 1 N #0 ARG 569.A 1HH1 -0.197 2.837

#1 UNL 1 C #0 TYR 689.A HD1 -0.205 2.965

#1 UNL 1 C #0 GLN 573.A CB -0.207 3.847

#1 UNL 1 C #0 ALA 580.A CB -0.222 3.862

#1 UNL 1 C #0 GLN 573.A CD -0.232 3.692

#1 UNL 1 C #0 ILE 589.A O -0.240 3.480

#1 UNL 1 C #0 ARG 569.A 1HH2 -0.250 3.010

#1 UNL 1 C #0 ILE 589.A CG2 -0.255 3.895

#1 UNL 1 O #0 ALA 580.A O -0.263 3.163

#1 UNL 1 C #0 LYS 577.A CE -0.265 3.905

#1 UNL 1 N #0 GLY 590.A N -0.274 3.539

#1 UNL 1 C #0 TYR 689.A CG   -0.276 3.736

#1 UNL 1 C #0 GLN 573.A OE1   -0.282 3.522

#1 UNL 1 C #0 ILE 589.A CA   -0.287 3.927

#1 UNL 1 N #0 ALA 580.A CA   -0.290 3.810

#1 UNL 1 C #0 TYR 689.A CE2   -0.293 3.873

#1 UNL 1 C #0 ILE 579.A CB   -0.293 3.933

#1 UNL 1 C #0 TYR 689.A CE2   -0.300 3.760

#1 UNL 1 C #0 ALA 580.A CA   -0.302 4.062

#1 UNL 1 C #0 ILE 494.A O   -0.307 3.547

#1 UNL 1 C #0 ARG 569.A 2HH2 -0.316 2.926

#1 UNL 1 C #0 ALA 580.A N   -0.319 3.704

#1 UNL 1 C #0 GLY 590.A N   -0.329 3.714

#1 UNL 1 C #0 TYR 689.A CE2   -0.346 3.656

#1 UNL 1 C #0 ALA 580.A CA   -0.349 3.989

#1 UNL 1 C #0 LEU 576.A O   -0.362 3.602

#1 UNL 1 C #0 LEU 576.A CD2   -0.363 4.003

#1 UNL 1 C #0 TYR 689.A HH   -0.369 3.129

Pharmacophore 3:

469 contacts

atom1 atom2 overlap distance

#1 UNL 1 C #0 SER 501.A OG   3.206 0.174

#1 UNL 1 C #0 SER 501.A CA   2.973 0.787

#1 UNL 1 C #0 ALA 502.A C   2.947 0.633

#1 UNL 1 C #0 LYS 511.A CE   2.892 0.868

#1 UNL 1 C #0 SER 501.A CB 2.861 0.899

#1 UNL 1 C #0 SER 501.A C 2.829 0.751

#1 UNL 1 C #0 SER 501.A N 2.819 0.686

#1 UNL 1 C #0 LYS 500.A C 2.679 0.901

#1 UNL 1 C #0 LYS 500.A CA 2.676 1.084

#1 UNL 1 C #0 SER 501.A CA 2.554 1.206

#1 UNL 1 C #0 LYS 511.A NZ 2.536 0.969

#1 UNL 1 C #0 ALA 502.A N 2.530 0.975

#1 UNL 1 C #0 SER 501.A C 2.525 1.055

#1 UNL 1 C #0 GLY 503.A N 2.520 0.985

#1 UNL 1 C #0 GLY 503.A CA 2.518 1.242

#1 UNL 1 C #0 LYS 500.A C 2.473 1.107

#1 UNL 1 C #0 ASP 499.A O 2.449 0.911

#1 UNL 1 C #0 ALA 502.A CA 2.442 1.318

#1 UNL 1 C #0 LYS 500.A O 2.438 0.922

#1 UNL 1 C #0 ALA 502.A CA 2.394 1.366

#1 UNL 1 C #0 ALA 502.A H 2.390 0.490

#1 UNL 1 C #0 SER 501.A CB 2.379 1.381

#1 UNL 1 C #0 GLY 503.A N 2.357 1.148

#1 UNL 1 C #0 SER 501.A CA 2.297 1.463

#1 UNL 1 C #0 LYS 500.A N 2.284 1.221

#1 UNL 1 C #0 ALA 502.A C 2.283 1.297

#1 UNL 1 C #0 GLY 503.A CA 2.272 1.488

#1 UNL 1 C #0 GLY 559.A O 2.237 1.123

#1 UNL 1 C #0 ASP 499.A C 2.158 1.422

#1 UNL 1 C #0 ILE 539.A O 2.146 1.214

#1 UNL 1 C #0 ALA 502.A N 2.126 1.379

#1 UNL 1 C #0 ASP 499.A C 2.024 1.556

#1 UNL 1 C #0 SER 501.A CB 2.018 1.742

#1 UNL 1 C #0 ALA 502.A C 2.013 1.567

#1 UNL 1 C #0 SER 501.A N 1.995 1.510

#1 UNL 1 C #0 SER 501.A CA 1.940 1.820

#1 UNL 1 C #0 LYS 511.A HZ1 1.937 0.943

#1 UNL 1 C #0 LYS 500.A C 1.924 1.656

#1 UNL 1 C #0 SER 501.A C 1.919 1.661

#1 UNL 1 C #0 LYS 500.A CA 1.909 1.851

#1 UNL 1 C #0 SER 501.A O 1.905 1.455

#1 UNL 1 C #0 ALA 502.A N 1.904 1.601

#1 UNL 1 C #0 GLY 503.A H 1.880 1.000

#1 UNL 1 C #0 SER 501.A OG 1.870 1.510

#1 UNL 1 C #0 GLY 503.A N 1.859 1.646

#1 UNL 1 C #0 SER 501.A C 1.857 1.723

#1 UNL 1 C #0 GLY 503.A H 1.843 1.037

#1 UNL 1 C #0 LYS 511.A NZ 1.823 1.682

#1 UNL 1 C #0 ALA 502.A N 1.794 1.711

#1 UNL 1 C #0 GLY 503.A CA 1.789 1.971

#1 UNL 1 C #0 SER 501.A N 1.783 1.722

#1 UNL 1 C #0 SER 501.A CA 1.778 1.982

#1 UNL 1 C #0 SER 501.A OG 1.749 1.631

#1 UNL 1 C #0 ALA 502.A O 1.729 1.631

#1 UNL 1 C #0 SER 501.A HG 1.729 1.151

#1 UNL 1 C #0 GLY 559.A C 1.693 1.887

#1 UNL 1 C #0 ALA 502.A CA 1.683 2.077

#1 UNL 1 C #0 ASP 499.A O 1.679 1.681

#1 UNL 1 C #0 LYS 500.A CA 1.654 2.106

#1 UNL 1 C #0 SER 501.A CB 1.640 2.120

#1 UNL 1 C #0 ILE 539.A C 1.626 1.954

#1 UNL 1 C #0 GLY 503.A N 1.622 1.883

#1 UNL 1 C #0 SER 501.A CA 1.607 2.153

#1 UNL 1 C #0 LYS 511.A HZ1 1.604 1.276

#1 UNL 1 C #0 ASN 507.A 1HD2 1.604 1.276

#1 UNL 1 C #0 LYS 500.A O 1.569 1.791

#1 UNL 1 C #0 VAL 560.A CA 1.554 2.206

#1 UNL 1 C #0 GLY 503.A N 1.551 1.954

#1 UNL 1 C #0 LYS 511.A HZ1 1.539 1.341

#1 UNL 1 C #0 GLY 503.A C 1.502 2.078

#1 UNL 1 C #0 ALA 502.A O 1.495 1.865

#1 UNL 1 C #0 SER 501.A CA 1.488 2.272

#1 UNL 1 C #0 ALA 502.A N 1.482 2.023

#1 UNL 1 C #0 LYS 511.A CD 1.476 2.284

#1 UNL 1 C #0 ASN 507.A OD1 1.459 1.901

#1 UNL 1 C #0 LYS 500.A C 1.457 2.123

#1 UNL 1 C #0 ALA 502.A H 1.449 1.431

#1 UNL 1 C #0 ASP 499.A O 1.442 1.918

#1 UNL 1 C #0 SER 501.A H 1.442 1.438

#1 UNL 1 C #0 ALA 502.A C 1.436 2.144

#1 UNL 1 C #0 THR 540.A CA 1.419 2.341

#1 UNL 1 C #0 SER 501.A N 1.417 2.088

#1 UNL 1 C #0 LYS 500.A N 1.412 2.093

#1 UNL 1 C #0 SER 501.A O 1.409 1.951

#1 UNL 1 C #0 ALA 502.A CA 1.403 2.357

#1 UNL 1 C #0 SER 501.A C 1.363 2.217

#1 UNL 1 C #0 SER 501.A CB 1.352 2.408

#1 UNL 1 C #0 LYS 511.A CE 1.342 2.418

#1 UNL 1 C #0 SER 501.A N 1.321 2.184

#1 UNL 1 C #0 LYS 511.A HZ2 1.313 1.567

#1 UNL 1 C #0 ASN 507.A ND2 1.309 2.196

#1 UNL 1 C #0 SER 501.A CA 1.305 2.455

#1 UNL 1 C #0 LYS 511.A NZ 1.299 2.206

#1 UNL 1 C #0 LYS 500.A CA 1.288 2.472

#1 UNL 1 C #0 LYS 500.A CB 1.283 2.477

#1 UNL 1 C #0 SER 501.A C 1.278 2.302

#1 UNL 1 C #0 GLY 503.A H 1.272 1.608

#1 UNL 1 C #0 SER 501.A O 1.251 2.109

#1 UNL 1 C #0 SER 561.A N 1.250 2.255

#1 UNL 1 C #0 ALA 502.A CA 1.247 2.513

#1 UNL 1 C #0 ALA 502.A CB 1.244 2.516

#1 UNL 1 C #0 ALA 502.A CB 1.241 2.519

#1 UNL 1 C #0 GLY 503.A CA 1.233 2.527

#1 UNL 1 C #0 ALA 502.A O 1.232 2.128

#1 UNL 1 C #0 LYS 500.A CA 1.211 2.549

#1 UNL 1 C #0 SER 501.A CA 1.210 2.550

#1 UNL 1 C #0 VAL 560.A N 1.200 2.305

#1 UNL 1 C #0 SER 501.A CB 1.186 2.574

#1 UNL 1 C #0 LYS 500.A C 1.171 2.409

#1 UNL 1 C #0 SER 501.A N 1.165 2.340

#1 UNL 1 C #0 ALA 502.A CB 1.142 2.618

#1 UNL 1 C #0 LYS 511.A NZ 1.134 2.371

#1 UNL 1 C #0 ALA 502.A CA 1.112 2.648

#1 UNL 1 C #0 LYS 500.A C 1.108 2.472

#1 UNL 1 C #0 ASP 499.A CA 1.104 2.656

#1 UNL 1 C #0 VAL 560.A CA 1.101 2.659

#1 UNL 1 O #0 LYS 511.A HZ3 1.094 1.366

#1 UNL 1 C #0 THR 540.A N 1.089 2.416

#1 UNL 1 C #0 ASP 499.A O 1.084 2.276

#1 UNL 1 C #0 SER 501.A CA 1.076 2.684

#1 UNL 1 C #0 LYS 511.A HZ3 1.075 1.805

#1 UNL 1 C #0 GLY 503.A CA 1.073 2.687

#1 UNL 1 C #0 LYS 500.A O 1.064 2.296

#1 UNL 1 O #0 LYS 511.A NZ 1.063 2.022

#1 UNL 1 C #0 LYS 511.A HZ2 1.054 1.826

#1 UNL 1 C #0 VAL 560.A C 1.054 2.526

#1 UNL 1 C #0 ALA 502.A C 1.051 2.529

#1 UNL 1 C #0 ASP 499.A O 1.029 2.331

#1 UNL 1 C #0 LYS 500.A C 1.026 2.554

#1 UNL 1 C #0 GLY 503.A N 1.020 2.485

#1 UNL 1 C #0 SER 501.A N 1.019 2.486

#1 UNL 1 C #0 VAL 560.A C 1.013 2.567

#1 UNL 1 C #0 ALA 502.A N 0.996 2.509

#1 UNL 1 C #0 LYS 500.A O 0.994 2.366

#1 UNL 1 C #0 ASN 507.A 1HD2 0.980 1.900

#1 UNL 1 C #0 LYS 500.A N 0.980 2.525

#1 UNL 1 C #0 ALA 502.A CA 0.974 2.786

#1 UNL 1 C #0 LYS 511.A NZ 0.970 2.535

#1 UNL 1 C #0 ASP 499.A C 0.964 2.616

#1 UNL 1 C #0 LYS 500.A O 0.964 2.396

#1 UNL 1 C #0 ILE 562.A CG1 0.957 2.803

#1 UNL 1 C #0 SER 501.A H 0.950 1.930

#1 UNL 1 C #0 LYS 511.A HZ2 0.948 1.932

#1 UNL 1 C #0 ALA 502.A N 0.941 2.564

#1 UNL 1 C #0 VAL 560.A O 0.937 2.423

#1 UNL 1 C #0 ASN 507.A ND2 0.923 2.582

#1 UNL 1 C #0 GLY 559.A O 0.917 2.443

#1 UNL 1 C #0 ASP 499.A C 0.915 2.665

#1 UNL 1 C #0 ASP 499.A O 0.915 2.445

#1 UNL 1 C #0 GLY 503.A H 0.895 1.985

#1 UNL 1 C #0 ILE 539.A O 0.886 2.474

#1 UNL 1 C #0 SER 561.A H 0.882 1.998

#1 UNL 1 C #0 SER 501.A HG 0.880 2.000

#1 UNL 1 C #0 LYS 500.A CA 0.876 2.884

#1 UNL 1 C #0 SER 501.A CB 0.874 2.886

#1 UNL 1 C #0 SER 501.A OG 0.870 2.510

#1 UNL 1 C #0 SER 501.A CB 0.858 2.902

#1 UNL 1 C #0 SER 501.A C 0.852 2.728

#1 UNL 1 C #0 ASN 507.A CG 0.846 2.734

#1 UNL 1 C #0 SER 501.A OG 0.844 2.536

#1 UNL 1 C #0 LYS 511.A NZ 0.840 2.665

#1 UNL 1 C #0 SER 561.A N 0.834 2.671

#1 UNL 1 C #0 SER 561.A CA 0.833 2.927

#1 UNL 1 C #0 SER 501.A C 0.827 2.753

#1 UNL 1 C #0 LYS 511.A HZ2 0.822 2.058

#1 UNL 1 C #0 SER 501.A N 0.807 2.698

#1 UNL 1 C #0 SER 561.A CA 0.801 2.959

#1 UNL 1 C #0 GLY 503.A O 0.800 2.560

#1 UNL 1 C #0 SER 501.A C 0.799 2.781

#1 UNL 1 C #0 SER 501.A O 0.791 2.569

#1 UNL 1 C #0 LYS 511.A HZ1 0.778 2.102

#1 UNL 1 C #0 GLY 503.A CA 0.777 2.983

#1 UNL 1 C #0 LYS 511.A HZ1 0.776 2.104

#1 UNL 1 C #0 SER 501.A C 0.766 2.814

#1 UNL 1 C #0 THR 540.A CA 0.764 2.996

#1 UNL 1 C #0 ASN 507.A OD1 0.755 2.605

#1 UNL 1 C #0 GLY 503.A C 0.749 2.831

#1 UNL 1 C #0 ALA 502.A N 0.734 2.771

#1 UNL 1 C #0 SER 501.A C 0.733 2.847

#1 UNL 1 C #0 VAL 560.A C 0.729 2.851

#1 UNL 1 C #0 LYS 500.A CB 0.729 3.031

#1 UNL 1 C #0 PHE 504.A N 0.722 2.783

#1 UNL 1 C #0 SER 561.A N 0.717 2.788

#1 UNL 1 C #0 GLY 503.A C 0.716 2.864

#1 UNL 1 C #0 LYS 500.A CA 0.707 3.053

#1 UNL 1 C #0 ASP 499.A CA 0.701 3.059

#1 UNL 1 C #0 LYS 500.A H 0.691 2.189

#1 UNL 1 C #0 SER 501.A CA 0.688 3.072

#1 UNL 1 C #0 ASP 499.A CB 0.682 3.078

#1 UNL 1 C #0 ASP 499.A C 0.681 2.899

#1 UNL 1 C #0 ALA 502.A H 0.673 2.207

#1 UNL 1 C #0 LYS 500.A CG 0.661 3.099

#1 UNL 1 C #0 PHE 504.A H 0.660 2.220

#1 UNL 1 C #0 VAL 560.A C 0.646 2.934

#1 UNL 1 C #0 LYS 511.A HZ1 0.643 2.237

#1 UNL 1 C #0 SER 501.A H 0.639 2.241

#1 UNL 1 C #0 ASN 507.A CG 0.638 2.942

#1 UNL 1 C #0 LYS 511.A NZ 0.638 2.867

#1 UNL 1 C #0 LYS 511.A HZ3 0.638 2.242

#1 UNL 1 C #0 LYS 511.A HZ1 0.630 2.250

#1 UNL 1 C #0 LYS 500.A CB 0.625 3.135

#1 UNL 1 C #0 SER 501.A OG 0.623 2.757

#1 UNL 1 C #0 LYS 500.A O 0.616 2.744

#1 UNL 1 C #0 GLU 665.A OE2 0.609 2.751

#1	UNL 1 C	#0	LYS	500.A	C	0.602	2.978
#1	UNL 1 C	#0	SER	501.A	N	0.598	2.907
#1	UNL 1 C	#0	ALA	502.A	N	0.596	2.909
#1	UNL 1 C	#0	SER	501.A	O	0.594	2.766
#1	UNL 1 C	#0	ALA	502.A	CB	0.587	3.173
#1	UNL 1 C	#0	ASN	507.A	OD1	0.580	2.780
#1	UNL 1 C	#0	ASN	507.A	OD1	0.576	2.784
#1	UNL 1 C	#0	SER	561.A	CA	0.572	3.188
#1	UNL 1 C	#0	ASN	497.A	OD1	0.557	2.803
#1	UNL 1 C	#0	LYS	511.A	CE	0.556	3.204
#1	UNL 1 C	#0	ALA	502.A	C	0.552	3.028
#1	UNL 1 C	#0	THR	540.A	CA	0.550	3.210
#1	UNL 1 C	#0	ASN	507.A	OD1	0.540	2.820
#1	UNL 1 C	#0	ASN	507.A	CG	0.530	3.050
#1	UNL 1 C	#0	ALA	502.A	O	0.528	2.832
#1	UNL 1 C	#0	ASP	499.A	O	0.518	2.842
#1	UNL 1 C	#0	LYS	500.A	O	0.510	2.850
#1	UNL 1 C	#0	LYS	511.A	NZ	0.510	2.995
#1	UNL 1 C	#0	SER	501.A	C	0.491	3.089
#1	UNL 1 C	#0	THR	540.A	CB	0.490	3.270
#1	UNL 1 C	#0	GLY	503.A	H	0.487	2.393
#1	UNL 1 C	#0	LYS	500.A	CB	0.485	3.275
#1	UNL 1 C	#0	LYS	511.A	NZ	0.475	3.030
#1	UNL 1 C	#0	LYS	511.A	CG	0.475	3.285
#1	UNL 1 C	#0	SER	501.A	O	0.474	2.886

#1 UNL 1 C #0 SER 501.A CA 0.470 3.290

#1 UNL 1 C #0 SER 501.A CB 0.466 3.294

#1 UNL 1 C #0 GLY 559.A O 0.458 2.902

#1 UNL 1 C #0 ALA 502.A H 0.452 2.428

#1 UNL 1 O #0 LYS 500.A CA 0.450 2.890

#1 UNL 1 C #0 LYS 511.A HZ2 0.445 2.435

#1 UNL 1 C #0 GLY 503.A H 0.442 2.438

#1 UNL 1 C #0 ILE 539.A O 0.437 2.923

#1 UNL 1 C #0 LYS 511.A CE 0.437 3.323

#1 UNL 1 C #0 SER 501.A N 0.437 3.068

#1 UNL 1 C #0 ALA 502.A CB 0.435 3.325

#1 UNL 1 C #0 GLY 503.A N 0.433 3.072

#1 UNL 1 C #0 SER 501.A H 0.430 2.450

#1 UNL 1 C #0 GLY 559.A CA 0.429 3.331

#1 UNL 1 C #0 ILE 539.A CA 0.428 3.332

#1 UNL 1 C #0 LYS 500.A N 0.425 3.080

#1 UNL 1 C #0 THR 540.A HG1 0.419 2.461

#1 UNL 1 C #0 SER 501.A H 0.413 2.467

#1 UNL 1 C #0 LYS 511.A HZ3 0.402 2.478

#1 UNL 1 C #0 LYS 500.A C 0.401 3.179

#1 UNL 1 C #0 GLY 559.A C 0.400 3.180

#1 UNL 1 C #0 PHE 504.A N 0.399 3.106

#1 UNL 1 C #0 LYS 500.A N 0.395 3.110

#1 UNL 1 C #0 GLY 503.A N 0.389 3.116

#1 UNL 1 C #0 GLY 503.A CA 0.384 3.376

#1	UNL	1	C	#0	ALA 502.A	C	0.380	3.200
#1	UNL	1	C	#0	LYS 511.A	HZ2	0.378	2.502
#1	UNL	1	C	#0	LYS 511.A	CE	0.371	3.389
#1	UNL	1	C	#0	LYS 511.A	HZ3	0.370	2.510
#1	UNL	1	C	#0	SER 501.A	HG	0.366	2.514
#1	UNL	1	C	#0	THR 540.A	CB	0.361	3.399
#1	UNL	1	C	#0	ALA 502.A	N	0.360	3.145
#1	UNL	1	C	#0	ALA 502.A	CA	0.358	3.402
#1	UNL	1	C	#0	ALA 502.A	H	0.358	2.522
#1	UNL	1	C	#0	SER 501.A	CB	0.348	3.412
#1	UNL	1	C	#0	LYS 500.A	CA	0.344	3.416
#1	UNL	1	C	#0	SER 501.A	O	0.334	3.026
#1	UNL	1	C	#0	ALA 502.A	CB	0.329	3.431
#1	UNL	1	C	#0	ASN 507.A	OD1	0.328	3.032
#1	UNL	1	C	#0	GLY 503.A	H	0.316	2.564
#1	UNL	1	C	#0	SER 561.A	N	0.314	3.191
#1	UNL	1	C	#0	LYS 500.A	N	0.314	3.191
#1	UNL	1	C	#0	THR 540.A	OG1	0.309	3.071
#1	UNL	1	C	#0	VAL 560.A	CA	0.306	3.454
#1	UNL	1	C	#0	SER 561.A	OG	0.298	3.082
#1	UNL	1	C	#0	SER 501.A	N	0.294	3.211
#1	UNL	1	C	#0	LYS 511.A	HZ2	0.288	2.592
#1	UNL	1	C	#0	ILE 562.A	N	0.281	3.224
#1	UNL	1	C	#0	VAL 560.A	O	0.281	3.079
#1	UNL	1	O	#0	ASP 499.A	O	0.277	2.663

#1 UNL 1 C #0 LYS 500.A C 0.274 3.306

#1 UNL 1 C #0 SER 561.A OG 0.264 3.116

#1 UNL 1 C #0 GLY 559.A C 0.264 3.316

#1 UNL 1 C #0 SER 501.A N 0.264 3.241

#1 UNL 1 C #0 GLY 503.A C 0.257 3.323

#1 UNL 1 C #0 LYS 500.A CG 0.256 3.504

#1 UNL 1 C #0 LYS 500.A CG 0.255 3.505

#1 UNL 1 C #0 GLN 541.A H 0.246 2.634

#1 UNL 1 C #0 SER 501.A H 0.241 2.639

#1 UNL 1 O #0 LYS 511.A HZ2 0.236 2.224

#1 UNL 1 C #0 SER 501.A H 0.221 2.659

#1 UNL 1 C #0 SER 501.A OG 0.211 3.169

#1 UNL 1 C #0 SER 561.A H 0.206 2.674

#1 UNL 1 C #0 ALA 502.A H 0.200 2.680

#1 UNL 1 C #0 GLY 559.A O 0.199 3.161

#1 UNL 1 C #0 SER 561.A OG 0.197 3.183

#1 UNL 1 C #0 LYS 511.A CE 0.196 3.564

#1 UNL 1 C #0 VAL 560.A CA 0.190 3.570

#1 UNL 1 C #0 VAL 560.A N 0.186 3.319

#1 UNL 1 C #0 SER 501.A OG 0.184 3.196

#1 UNL 1 C #0 ASN 507.A 1HD2 0.179 2.701

#1 UNL 1 C #0 SER 561.A N 0.177 3.328

#1 UNL 1 C #0 VAL 560.A CB 0.177 3.583

#1 UNL 1 C #0 ALA 502.A C 0.176 3.404

#1 UNL 1 C #0 LYS 500.A O 0.162 3.198

#1 UNL 1 C #0 ILE 539.A C 0.147 3.433

#1 UNL 1 C #0 ASP 499.A N 0.145 3.360

#1 UNL 1 O #0 LYS 511.A HZ1 0.143 2.317

#1 UNL 1 C #0 SER 501.A CB 0.139 3.621

#1 UNL 1 C #0 SER 501.A CB 0.133 3.627

#1 UNL 1 C #0 GLN 541.A N 0.131 3.374

#1 UNL 1 C #0 LYS 511.A CE 0.131 3.629

#1 UNL 1 C #0 LYS 511.A HZ1 0.124 2.756

#1 UNL 1 C #0 LYS 500.A C 0.124 3.456

#1 UNL 1 C #0 GLY 503.A CA 0.122 3.638

#1 UNL 1 C #0 LYS 500.A CA 0.121 3.639

#1 UNL 1 C #0 SER 561.A CB 0.119 3.641

#1 UNL 1 C #0 ASP 499.A CA 0.118 3.642

#1 UNL 1 O #0 SER 501.A H 0.097 2.363

#1 UNL 1 C #0 ASN 507.A CG 0.096 3.484

#1 UNL 1 C #0 SER 561.A CA 0.083 3.677

#1 UNL 1 C #0 LYS 500.A CB 0.081 3.679

#1 UNL 1 C #0 LYS 511.A NZ 0.080 3.425

#1 UNL 1 C #0 LYS 511.A HZ1 0.080 2.800

#1 UNL 1 C #0 LYS 511.A CE 0.077 3.683

#1 UNL 1 C #0 LYS 500.A CG 0.075 3.685

#1 UNL 1 C #0 GLY 503.A N 0.071 3.434

#1 UNL 1 C #0 ASN 507.A ND2 0.065 3.440

#1 UNL 1 C #0 ILE 562.A H 0.064 2.816

#1 UNL 1 C #0 LYS 511.A HZ3 0.061 2.819

#1	UNL	1	C	#0	LYS	511.A	CB	0.057	3.703
#1	UNL	1	C	#0	ALA	502.A	N	0.053	3.452
#1	UNL	1	C	#0	LYS	500.A	CA	0.052	3.708
#1	UNL	1	C	#0	PHE	504.A	N	0.046	3.459
#1	UNL	1	C	#0	THR	540.A	C	0.042	3.538
#1	UNL	1	O	#0	SER	501.A	N	0.042	3.043
#1	UNL	1	C	#0	ILE	562.A	CD	0.041	3.719
#1	UNL	1	C	#0	SER	501.A	CA	0.039	3.721
#1	UNL	1	C	#0	LYS	500.A	N	0.035	3.470
#1	UNL	1	C	#0	SER	561.A	CB	0.035	3.725
#1	UNL	1	O	#0	LYS	511.A	CE	0.031	3.309
#1	UNL	1	C	#0	GLY	503.A	C	0.025	3.555
#1	UNL	1	C	#0	LYS	500.A	CA	0.024	3.736
#1	UNL	1	C	#0	VAL	560.A	N	0.023	3.482
#1	UNL	1	C	#0	ALA	502.A	CB	0.022	3.738
#1	UNL	1	C	#0	SER	561.A	H	0.015	2.865
#1	UNL	1	C	#0	GLY	503.A	CA	0.013	3.747
#1	UNL	1	C	#0	GLY	559.A	C	0.010	3.570
#1	UNL	1	C	#0	GLY	503.A	O	0.009	3.351
#1	UNL	1	C	#0	SER	501.A	O	0.009	3.351
#1	UNL	1	C	#0	LYS	511.A	CE	0.005	3.755
#1	UNL	1	C	#0	ASP	499.A	C	0.002	3.578
#1	UNL	1	C	#0	ALA	512.A	H	0.001	2.879
#1	UNL	1	C	#0	ALA	502.A	N	0.001	3.504
#1	UNL	1	C	#0	LYS	500.A	O	-0.000	3.360

#1 UNL 1 C #0 GLY 559.A CA -0.000 3.760

#1 UNL 1 C #0 LYS 511.A HZ2 -0.005 2.885

#1 UNL 1 C #0 ILE 562.A CA -0.007 3.767

#1 UNL 1 C #0 ALA 502.A CA -0.009 3.769

#1 UNL 1 C #0 ALA 502.A O -0.012 3.372

#1 UNL 1 C #0 ALA 502.A H -0.015 2.895

#1 UNL 1 C #0 LYS 511.A HZ1 -0.017 2.897

#1 UNL 1 C #0 SER 501.A O -0.017 3.377

#1 UNL 1 C #0 SER 501.A O -0.022 3.382

#1 UNL 1 C #0 LYS 500.A C -0.030 3.610

#1 UNL 1 C #0 LYS 511.A CD -0.033 3.793

#1 UNL 1 C #0 GLY 559.A O -0.033 3.393

#1 UNL 1 C #0 GLY 503.A H -0.038 2.918

#1 UNL 1 C #0 GLY 503.A N -0.047 3.552

#1 UNL 1 C #0 LYS 511.A HZ1 -0.053 2.933

#1 UNL 1 C #0 VAL 560.A O -0.056 3.416

#1 UNL 1 C #0 SER 501.A N -0.059 3.564

#1 UNL 1 C #0 SER 501.A H -0.060 2.940

#1 UNL 1 C #0 LYS 511.A CE -0.063 3.823

#1 UNL 1 C #0 ASN 497.A OD1 -0.064 3.424

#1 UNL 1 C #0 ASN 507.A CG -0.064 3.644

#1 UNL 1 C #0 ASP 499.A C -0.065 3.645

#1 UNL 1 C #0 LYS 511.A NZ -0.071 3.576

#1 UNL 1 C #0 ASN 507.A CG -0.073 3.653

#1 UNL 1 C #0 GLY 503.A H -0.078 2.958

#1 UNL 1 C #0 ALA 502.A CA -0.081 3.841

#1 UNL 1 C #0 LYS 500.A H -0.094 2.974

#1 UNL 1 C #0 VAL 560.A O -0.115 3.475

#1 UNL 1 C #0 VAL 560.A CA -0.116 3.876

#1 UNL 1 C #0 SER 501.A CA -0.119 3.879

#1 UNL 1 C #0 ILE 539.A N -0.124 3.629

#1 UNL 1 C #0 SER 561.A C -0.124 3.704

#1 UNL 1 C #0 THR 540.A C -0.132 3.712

#1 UNL 1 C #0 ASN 507.A 2HD2 -0.134 3.014

#1 UNL 1 C #0 SER 501.A N -0.137 3.642

#1 UNL 1 C #0 ASN 497.A OD1 -0.139 3.499

#1 UNL 1 C #0 ASN 507.A OD1 -0.144 3.504

#1 UNL 1 C #0 LYS 500.A C -0.145 3.725

#1 UNL 1 C #0 ASN 507.A OD1 -0.145 3.505

#1 UNL 1 C #0 LYS 500.A CB -0.149 3.909

#1 UNL 1 C #0 ILE 562.A CB -0.151 3.911

#1 UNL 1 O #0 LYS 500.A CB -0.153 3.493

#1 UNL 1 C #0 VAL 560.A O -0.156 3.516

#1 UNL 1 C #0 GLY 503.A H -0.162 3.042

#1 UNL 1 C #0 LYS 500.A O -0.163 3.523

#1 UNL 1 C #0 SER 561.A H -0.177 3.057

#1 UNL 1 O #0 LYS 500.A C -0.181 3.341

#1 UNL 1 C #0 SER 501.A CA -0.186 3.946

#1 UNL 1 C #0 ALA 502.A H -0.195 3.075

#1 UNL 1 C #0 ASP 499.A C -0.196 3.776

#1 UNL 1 C #0 THR 540.A CB -0.198 3.958

#1 UNL 1 C #0 ASN 507.A ND2 -0.201 3.706

#1 UNL 1 C #0 ALA 502.A C -0.205 3.785

#1 UNL 1 C #0 SER 501.A O -0.207 3.567

#1 UNL 1 O #0 LYS 511.A HZ1 -0.209 2.669

#1 UNL 1 C #0 ASP 499.A N -0.211 3.716

#1 UNL 1 C #0 VAL 560.A CG2 -0.213 3.973

#1 UNL 1 C #0 ILE 539.A O -0.214 3.574

#1 UNL 1 C #0 GLU 665.A CD -0.224 3.804

#1 UNL 1 C #0 ASN 507.A CB -0.224 3.984

#1 UNL 1 C #0 LYS 500.A N -0.227 3.732

#1 UNL 1 C #0 ALA 502.A N -0.230 3.735

#1 UNL 1 C #0 SER 561.A C -0.240 3.820

#1 UNL 1 C #0 SER 501.A CA -0.243 4.003

#1 UNL 1 C #0 VAL 560.A O -0.245 3.605

#1 UNL 1 C #0 ALA 502.A CA -0.246 4.006

#1 UNL 1 C #0 ASP 499.A CB -0.248 4.008

#1 UNL 1 C #0 SER 561.A CA -0.251 4.011

#1 UNL 1 C #0 ALA 512.A N -0.252 3.757

#1 UNL 1 C #0 VAL 560.A CB -0.257 4.017

#1 UNL 1 C #0 ASP 499.A H -0.257 3.137

#1 UNL 1 C #0 LYS 500.A CB -0.258 4.018

#1 UNL 1 C #0 SER 501.A O -0.261 3.621

#1 UNL 1 C #0 ASP 499.A CA -0.261 4.021

#1 UNL 1 C #0 THR 540.A N -0.268 3.773

#1 UNL 1 C #0 LYS 511.A HZ3 -0.271 3.151

#1 UNL 1 C #0 SER 501.A H -0.272 3.152

#1 UNL 1 C #0 ILE 562.A CG1 -0.290 4.050

#1 UNL 1 C #0 GLY 559.A CA -0.293 4.053

#1 UNL 1 C #0 LYS 511.A NZ -0.295 3.800

#1 UNL 1 C #0 SER 561.A CB -0.298 4.058

#1 UNL 1 C #0 ASP 499.A CA -0.304 4.064

#1 UNL 1 C #0 VAL 560.A C -0.313 3.893

#1 UNL 1 C #0 ASN 507.A ND2 -0.315 3.820

#1 UNL 1 C #0 ASN 497.A CG -0.315 3.895

#1 UNL 1 C #0 ALA 502.A C -0.317 3.897

#1 UNL 1 C #0 VAL 560.A O -0.319 3.679

#1 UNL 1 C #0 SER 561.A CA -0.320 4.080

#1 UNL 1 C #0 LYS 511.A HZ3 -0.326 3.206

#1 UNL 1 C #0 ASN 507.A 2HD2 -0.329 3.209

#1 UNL 1 C #0 GLY 503.A O -0.330 3.690

#1 UNL 1 C #0 ASP 499.A C -0.335 3.915

#1 UNL 1 C #0 ASP 499.A CB -0.338 4.098

#1 UNL 1 C #0 LYS 500.A C -0.341 3.921

#1 UNL 1 C #0 LYS 500.A HZ2 -0.342 3.222

#1 UNL 1 C #0 SER 561.A CB -0.343 4.103

#1 UNL 1 C #0 ASN 507.A 1HD2 -0.344 3.224

#1 UNL 1 C #0 ASN 507.A CG -0.345 3.925

#1 UNL 1 C #0 GLN 541.A N -0.346 3.851

#1 UNL 1 C #0 ASN 507.A ND2 -0.347 3.852

#1 UNL 1 C #0 ALA 502.A H -0.347 3.227

#1 UNL 1 C #0 GLY 503.A N -0.348 3.853

#1 UNL 1 C #0 LYS 511.A HZ2 -0.355 3.235

#1 UNL 1 C #0 SER 501.A H -0.355 3.235

#1 UNL 1 C #0 GLY 559.A N -0.360 3.865

#1 UNL 1 C #0 LYS 500.A CA -0.371 4.131

#1 UNL 1 C #0 VAL 560.A C -0.372 3.952

#1 UNL 1 C #0 GLN 541.A H -0.372 3.252

#1 UNL 1 C #0 ASN 507.A ND2 -0.376 3.881

#1 UNL 1 C #0 ASN 507.A CA -0.378 4.138

#1 UNL 1 C #0 ALA 502.A O -0.379 3.739

#1 UNL 1 C #0 THR 540.A CA -0.391 4.151

#1 UNL 1 C #0 ASN 507.A CG -0.393 3.973

#1 UNL 1 C #0 PHE 504.A H -0.393 3.273

#1 UNL 1 C #0 VAL 560.A N -0.395 3.900

Pharmacophore 4:

362 contacts

atom1 atom2 overlap distance

#1 UNL 1 C #0 GLN 541.A CG 2.694 0.946

#1 UNL 1 C #0 SER 501.A OG 2.680 0.700

#1 UNL 1 C #0 ASN 543.A CA 2.660 0.980

#1 UNL 1 O #0 PHE 504.A CG 2.572 0.588

#1 UNL 1 C #0 GLN 541.A OE1 2.298 1.062

#1 UNL 1 C #0 ASN 543.A CG 2.255 1.055

#1 UNL 1 C #0 MET 542.A O 2.234 0.856

#1 UNL 1 C #0 ASN 543.A CB 2.143 1.347

#1 UNL 1 C #0 ASN 543.A ND2 2.109 1.126

#1 UNL 1 C #0 MET 542.A C 2.105 1.205

#1 UNL 1 C #0 MET 542.A H 2.100 0.660

#1 UNL 1 C #0 ASN 543.A CB 2.096 1.394

#1 UNL 1 C #0 ASN 543.A CG 2.091 1.369

#1 UNL 1 C #0 ASN 543.A ND2 2.068 1.317

#1 UNL 1 C #0 ASN 543.A 2HD2 2.062 0.548

#1 UNL 1 O #0 PHE 504.A CD1 2.045 1.115

#1 UNL 1 C #0 ASN 543.A OD1 1.962 1.278

#1 UNL 1 C #0 ASN 543.A 1HD2 1.960 0.650

#1 UNL 1 C #0 PHE 504.A CD1 1.955 1.625

#1 UNL 1 C #0 MET 542.A C 1.918 1.392

#1 UNL 1 C #0 SER 501.A CB 1.913 1.847

#1 UNL 1 C #0 GLN 541.A CG 1.900 1.590

#1 UNL 1 C #0 ASN 543.A 2HD2 1.890 0.870

#1 UNL 1 C #0 ASN 543.A ND2 1.887 1.348

#1 UNL 1 C #0 MET 542.A O 1.880 1.210

#1 UNL 1 C #0 GLN 541.A CD 1.867 1.713

#1 UNL 1 C #0 MET 542.A N 1.850 1.535

#1 UNL 1 C #0 PHE 504.A HD1 1.811 1.069

#1 UNL 1 C #0 ASN 543.A CB 1.807 1.833

#1 UNL 1 O #0 GLN 541.A CB 1.781 1.559

#1 UNL 1 O #0 MET 668.A CB 1.774 1.566

#1 UNL 1 C #0 GLN 541.A CD 1.768 1.692

#1 UNL 1 C #0 ASN 543.A CB 1.765 1.875

#1 UNL 1 C #0 GLN 541.A CD 1.747 1.563

#1 UNL 1 C #0 ASN 543.A CG 1.746 1.714

#1 UNL 1 O #0 GLN 541.A CA 1.738 1.602

#1 UNL 1 C #0 ASN 543.A CA 1.711 1.779

#1 UNL 1 C #0 ASN 543.A ND2 1.701 1.684

#1 UNL 1 C #0 GLN 541.A CA 1.686 1.804

#1 UNL 1 C #0 MET 542.A N 1.680 1.555

#1 UNL 1 O #0 MET 542.A O 1.677 1.223

#1 UNL 1 C #0 ASN 543.A 1HD2 1.649 1.111

#1 UNL 1 C #0 PHE 504.A CG 1.646 1.934

#1 UNL 1 C #0 MET 542.A H 1.639 0.971

#1 UNL 1 C #0 SER 501.A HG 1.637 1.243

#1 UNL 1 O #0 PHE 504.A CB 1.604 1.736

#1 UNL 1 O #0 MET 668.A CG 1.584 1.756

#1 UNL 1 C #0 ASN 543.A ND2 1.576 1.659

#1 UNL 1 C #0 ASN 543.A ND2 1.568 1.817

#1 UNL 1 C #0 GLN 541.A CB 1.552 1.938

#1 UNL 1 C #0 ASN 543.A N 1.552 1.683

#1 UNL 1 C #0 PHE 504.A CB 1.542 2.218

#1 UNL 1 C #0 ASN 507.A ND2 1.540 1.965

#1 UNL 1 C #0 MET 542.A CA 1.528 1.962

#1 UNL 1 C #0 ASN 543.A N 1.525 1.860

#1 UNL 1 C #0 PHE 504.A CD1 1.523 2.057

#1 UNL 1 C #0 ASN 543.A CA 1.521 1.969

#1 UNL 1 O #0 MET 542.A CB 1.482 1.858

#1 UNL 1 C #0 GLN 541.A CB 1.481 2.159

#1 UNL 1 C #0 PHE 504.A HD1 1.466 1.414

#1 UNL 1 O #0 PHE 504.A CD2 1.454 1.706

#1 UNL 1 O #0 MET 542.A C 1.446 1.674

#1 UNL 1 C #0 PHE 504.A CB 1.416 2.344

#1 UNL 1 C #0 GLN 541.A CG 1.412 2.348

#1 UNL 1 C #0 GLN 541.A NE2 1.383 2.002

#1 UNL 1 C #0 GLN 541.A CB 1.371 2.389

#1 UNL 1 O #0 PHE 504.A HD1 1.367 1.093

#1 UNL 1 C #0 GLN 541.A NE2 1.362 1.873

#1 UNL 1 O #0 GLN 541.A CG 1.358 1.982

#1 UNL 1 C #0 ASN 543.A CG 1.348 2.112

#1 UNL 1 C #0 ASN 507.A CB 1.330 2.430

#1 UNL 1 O #0 ASN 507.A CG 1.315 1.845

#1 UNL 1 C #0 SER 501.A OG 1.307 2.073

#1 UNL 1 O #0 ASN 507.A ND2 1.304 1.781

#1 UNL 1 O #0 GLN 541.A CD 1.301 1.859

#1 UNL 1 C #0 ASN 507.A ND2 1.298 2.207

#1 UNL 1 C #0 MET 542.A O 1.293 1.797

#1 UNL 1 C #0 MET 668.A O 1.288 1.952

#1 UNL 1 C #0 MET 542.A C 1.274 2.036

#1 UNL 1 C #0 MET 542.A CA 1.265 2.225

#1 UNL 1 C #0 GLN 541.A CA 1.262 2.378

#1 UNL 1 O #0 ASN 507.A CB 1.247 2.093

#1 UNL 1 C #0 GLN 541.A CG 1.195 2.295

#1 UNL 1 C #0 GLN 541.A NE2 1.194 2.191

#1 UNL 1 C #0 GLN 541.A C 1.189 2.271

#1 UNL 1 C #0 ASN 543.A OD1 1.188 2.052

#1 UNL 1 O #0 PHE 504.A CD1 1.187 1.973

#1 UNL 1 C #0 ASN 543.A OD1 1.160 1.930

#1 UNL 1 C #0 ASN 543.A CG 1.148 2.162

#1 UNL 1 O #0 MET 542.A CA 1.147 2.193

#1 UNL 1 C #0 SER 501.A CA 1.143 2.617

#1 UNL 1 C #0 ASN 507.A 1HD2 1.140 1.740

#1 UNL 1 C #0 PHE 504.A CB 1.134 2.626

#1 UNL 1 C #0 ASN 543.A C 1.131 2.329

#1 UNL 1 C #0 GLN 541.A OE1 1.114 2.246

#1 UNL 1 C #0 MET 542.A C 1.110 2.350

#1 UNL 1 O #0 GLN 541.A OE1 1.092 1.848

#1 UNL 1 O #0 GLN 541.A CG 1.088 2.252

#1 UNL 1 C #0 SER 501.A HG 1.076 1.804

#1 UNL 1 C #0 ASN 543.A CB 1.070 2.570

#1 UNL 1 C #0 ASN 543.A 1HD2 1.068 1.542

#1 UNL 1 C #0 GLN 541.A C 1.044 2.266

#1 UNL 1 C #0 MET 542.A N 1.030 2.205

#1 UNL 1 C #0 ASN 507.A CB 1.026 2.734

#1 UNL 1 C #0 MET 542.A N 1.007 2.228

#1 UNL 1 O #0 GLN 541.A CB 0.989 2.351

#1 UNL 1 O #0 PHE 504.A CE1   0.971 2.189

#1 UNL 1 C #0 GLN 541.A 2HE2 0.966 1.644

#1 UNL 1 C #0 ASN 543.A CG   0.959 2.351

#1 UNL 1 C #0 ASN 543.A N   0.956 2.279

#1 UNL 1 C #0 GLN 541.A OE1   0.956 2.404

#1 UNL 1 O #0 ASN 543.A CB   0.956 2.384

#1 UNL 1 C #0 GLN 541.A 2HE2 0.945 1.815

#1 UNL 1 C #0 ASN 507.A 2HD2 0.941 1.939

#1 UNL 1 C #0 MET 542.A O   0.937 2.303

#1 UNL 1 C #0 ASN 507.A CG   0.935 2.645

#1 UNL 1 C #0 ASN 507.A CG   0.933 2.647

#1 UNL 1 C #0 MET 542.A CB   0.932 2.558

#1 UNL 1 C #0 GLN 541.A 2HE2 0.932 1.678

#1 UNL 1 C #0 GLN 541.A CA   0.930 2.710

#1 UNL 1 C #0 PHE 504.A CG   0.898 2.682

#1 UNL 1 C #0 ASN 543.A CA   0.897 2.593

#1 UNL 1 C #0 MET 542.A CA   0.877 2.763

#1 UNL 1 O #0 MET 542.A O   0.862 2.078

#1 UNL 1 O #0 GLN 541.A CD   0.853 2.307

#1 UNL 1 C #0 GLN 541.A CD   0.853 2.607

#1 UNL 1 C #0 ASN 543.A 2HD2 0.849 1.761

#1 UNL 1 O #0 GLN 541.A OE1   0.844 2.096

#1 UNL 1 C #0 MET 668.A CB   0.835 2.925

#1 UNL 1 C #0 GLN 541.A NE2   0.832 2.403

#1 UNL 1 C #0 MET 542.A C   0.832 2.478

#1 UNL 1 C #0 PHE 504.A CE1 0.832 2.748

#1 UNL 1 C #0 ASN 507.A 1HD2 0.815 2.065

#1 UNL 1 C #0 ASN 543.A CG 0.802 2.508

#1 UNL 1 C #0 MET 542.A H 0.799 1.811

#1 UNL 1 O #0 ASN 543.A 2HD2 0.798 1.662

#1 UNL 1 C #0 MET 542.A CA 0.792 2.698

#1 UNL 1 C #0 PHE 504.A CG 0.792 2.788

#1 UNL 1 C #0 ASN 507.A 2HD2 0.786 2.094

#1 UNL 1 C #0 ASN 543.A CG 0.783 2.677

#1 UNL 1 C #0 MET 668.A CG 0.774 2.986

#1 UNL 1 C #0 GLN 541.A CA 0.773 2.987

#1 UNL 1 C #0 ASN 543.A N 0.768 2.467

#1 UNL 1 C #0 ASN 543.A 1HD2 0.760 2.000

#1 UNL 1 C #0 PHE 504.A CA 0.747 3.013

#1 UNL 1 O #0 ASN 507.A 2HD2 0.745 1.715

#1 UNL 1 C #0 ASN 507.A ND2 0.743 2.762

#1 UNL 1 C #0 GLN 541.A 2HE2 0.733 2.027

#1 UNL 1 O #0 MET 542.A C 0.730 2.430

#1 UNL 1 C #0 GLN 541.A 2HE2 0.728 1.882

#1 UNL 1 O #0 ASN 543.A N 0.724 2.321

#1 UNL 1 O #0 MET 542.A N 0.723 2.362

#1 UNL 1 C #0 GLN 541.A CG 0.718 2.922

#1 UNL 1 C #0 ASN 507.A 2HD2 0.696 2.184

#1 UNL 1 C #0 ASN 507.A CG 0.674 2.906

#1 UNL 1 C #0 GLN 541.A C 0.671 2.789

#1 UNL 1 C #0 GLN 541.A NE2 0.668 2.567

#1 UNL 1 O #0 ASN 543.A CA 0.668 2.632

#1 UNL 1 C #0 MET 542.A N 0.659 2.726

#1 UNL 1 O #0 PHE 504.A HD1 0.654 1.806

#1 UNL 1 O #0 ASN 543.A ND2 0.651 2.434

#1 UNL 1 C #0 ASN 507.A ND2 0.646 2.859

#1 UNL 1 C #0 GLN 541.A NE2 0.636 2.869

#1 UNL 1 O #0 ASN 543.A 1HD2 0.625 1.835

#1 UNL 1 C #0 GLN 541.A 2HE2 0.614 1.996

#1 UNL 1 O #0 PHE 504.A CE2 0.611 2.549

#1 UNL 1 C #0 ASN 543.A 2HD2 0.607 2.003

#1 UNL 1 C #0 GLN 541.A OE1 0.591 2.499

#1 UNL 1 C #0 SER 501.A CB 0.588 3.172

#1 UNL 1 C #0 GLN 541.A NE2 0.581 2.654

#1 UNL 1 C #0 MET 542.A O 0.569 2.521

#1 UNL 1 C #0 ASN 507.A OD1 0.561 2.799

#1 UNL 1 C #0 GLN 541.A CD 0.549 2.761

#1 UNL 1 C #0 MET 542.A H 0.542 2.068

#1 UNL 1 O #0 PHE 504.A CE1 0.534 2.626

#1 UNL 1 C #0 MET 668.A O 0.533 2.557

#1 UNL 1 C #0 PHE 504.A CD1 0.526 3.054

#1 UNL 1 O #0 MET 542.A H 0.525 1.935

#1 UNL 1 C #0 GLN 541.A OE1 0.512 2.848

#1 UNL 1 C #0 GLN 541.A C 0.510 2.800

#1 UNL 1 O #0 ASN 507.A 2HD2 0.508 1.952

#1 UNL 1 C #0 MET 542.A N 0.500 2.735

#1 UNL 1 C #0 ASN 543.A 1HD2 0.491 2.269

#1 UNL 1 C #0 GLN 541.A CB 0.479 3.161

#1 UNL 1 C #0 PHE 504.A CD2 0.476 3.104

#1 UNL 1 C #0 ASN 543.A CA 0.474 3.016

#1 UNL 1 O #0 ASN 543.A CA 0.470 2.870

#1 UNL 1 C #0 LEU 544.A N 0.466 2.919

#1 UNL 1 O #0 PHE 504.A CA 0.454 2.886

#1 UNL 1 O #0 GLN 541.A N 0.453 2.632

#1 UNL 1 C #0 ASN 543.A OD1 0.447 2.793

#1 UNL 1 C #0 PHE 504.A CA 0.437 3.323

#1 UNL 1 O #0 MET 542.A CA 0.436 2.864

#1 UNL 1 C #0 ASN 543.A N 0.436 2.799

#1 UNL 1 O #0 ASN 543.A ND2 0.434 2.651

#1 UNL 1 C #0 GLN 541.A CD 0.428 3.152

#1 UNL 1 O #0 PHE 504.A CZ 0.423 2.737

#1 UNL 1 C #0 ASN 543.A CB 0.418 3.072

#1 UNL 1 C #0 SER 501.A OG 0.406 2.974

#1 UNL 1 C #0 PHE 504.A HD1 0.396 2.484

#1 UNL 1 C #0 GLN 541.A OE1 0.393 2.847

#1 UNL 1 C #0 ASN 543.A OD1 0.389 2.701

#1 UNL 1 C #0 ASN 543.A CB 0.371 3.119

#1 UNL 1 C #0 GLN 541.A CD 0.362 3.218

#1 UNL 1 O #0 GLN 541.A NE2 0.354 2.731

#1 UNL 1 C #0 MET 542.A CA 0.353 3.137

#1 UNL 1 C #0 ASN 507.A CB 0.350 3.410

#1 UNL 1 C #0 PHE 504.A CE1 0.350 3.230

#1 UNL 1 C #0 SER 501.A C 0.349 3.231

#1 UNL 1 C #0 ASN 543.A OD1 0.343 2.897

#1 UNL 1 C #0 ASN 507.A CG 0.332 3.248

#1 UNL 1 O #0 MET 542.A CG 0.326 3.014

#1 UNL 1 O #0 GLN 541.A CG 0.326 3.014

#1 UNL 1 O #0 MET 668.A O 0.321 2.619

#1 UNL 1 C #0 MET 668.A C 0.315 3.145

#1 UNL 1 C #0 SER 501.A HG 0.308 2.572

#1 UNL 1 O #0 GLN 541.A C 0.297 2.863

#1 UNL 1 C #0 GLN 541.A CB 0.295 3.465

#1 UNL 1 C #0 GLN 541.A CG 0.290 3.470

#1 UNL 1 C #0 ASN 543.A CA 0.280 3.360

#1 UNL 1 C #0 MET 668.A CG 0.279 3.481

#1 UNL 1 C #0 MET 542.A CB 0.271 3.369

#1 UNL 1 O #0 ASN 507.A CB 0.262 3.078

#1 UNL 1 O #0 MET 668.A CA 0.258 3.082

#1 UNL 1 C #0 ASN 543.A CA 0.252 3.388

#1 UNL 1 C #0 MET 542.A C 0.249 3.061

#1 UNL 1 C #0 ASN 543.A 2HD2 0.238 2.522

#1 UNL 1 O #0 MET 542.A CB 0.219 3.081

#1 UNL 1 C #0 ASN 543.A C 0.218 3.092

#1 UNL 1 O #0 ASN 507.A ND2 0.213 2.872

#1 UNL 1 C #0 MET 542.A C 0.213 3.247

#1 UNL 1 C #0 MET 542.A CB 0.211 3.279

#1 UNL 1 C #0 MET 668.A CB 0.209 3.551

#1 UNL 1 C #0 SER 501.A CB 0.208 3.552

#1 UNL 1 C #0 MET 668.A CG 0.207 3.553

#1 UNL 1 C #0 PHE 504.A CB 0.191 3.569

#1 UNL 1 C #0 GLN 541.A OE1 0.189 3.171

#1 UNL 1 C #0 MET 542.A H 0.183 2.577

#1 UNL 1 O #0 ASN 543.A N 0.179 2.906

#1 UNL 1 C #0 ASN 543.A CA 0.179 3.311

#1 UNL 1 C #0 GLY 503.A H 0.174 2.706

#1 UNL 1 O #0 MET 542.A CG 0.168 3.132

#1 UNL 1 C #0 ASN 543.A 1HD2 0.168 2.442

#1 UNL 1 C #0 ASN 543.A 2HD2 0.150 2.610

#1 UNL 1 O #0 ASN 507.A OD1 0.148 2.792

#1 UNL 1 C #0 ASN 543.A ND2 0.145 3.090

#1 UNL 1 C #0 PHE 504.A CA 0.139 3.621

#1 UNL 1 C #0 GLN 541.A CG 0.134 3.356

#1 UNL 1 C #0 ALA 502.A N 0.130 3.375

#1 UNL 1 C #0 ASN 543.A CB 0.118 3.372

#1 UNL 1 O #0 PHE 504.A CB 0.115 3.225

#1 UNL 1 O #0 GLN 541.A OE1 0.112 2.828

#1 UNL 1 C #0 ASN 507.A 2HD2 0.110 2.770

#1 UNL 1 C #0 ASN 543.A OD1 0.109 2.981

#1 UNL 1 C #0 ASN 543.A C 0.107 3.203

#1 UNL 1 C #0 MET 542.A O 0.096 3.144

#1 UNL 1 C #0 PHE 504.A CD1 0.084 3.496

#1 UNL 1 C #0 MET 668.A O 0.084 3.006

#1 UNL 1 C #0 ASN 543.A CB 0.079 3.561

#1 UNL 1 C #0 GLN 541.A C 0.079 3.231

#1 UNL 1 C #0 GLN 541.A C 0.077 3.233

#1 UNL 1 C #0 GLN 541.A 1HE2 0.059 2.701

#1 UNL 1 C #0 GLN 541.A CB 0.054 3.436

#1 UNL 1 O #0 PHE 504.A CG 0.046 3.114

#1 UNL 1 C #0 ASN 543.A H 0.035 2.725

#1 UNL 1 C #0 PHE 504.A HD1 0.025 2.855

#1 UNL 1 O #0 SER 501.A HG 0.024 2.436

#1 UNL 1 C #0 GLN 541.A CA 0.021 3.739

#1 UNL 1 C #0 ASN 507.A ND2 0.019 3.486

#1 UNL 1 C #0 MET 542.A CB 0.012 3.478

#1 UNL 1 O #0 MET 542.A C 0.004 3.156

#1 UNL 1 C #0 GLN 541.A CA 0.002 3.488

#1 UNL 1 O #0 ASN 507.A 1HD2 0.002 2.458

#1 UNL 1 C #0 PHE 504.A CB -0.000 3.760

#1 UNL 1 C #0 GLN 541.A N -0.003 3.238

#1 UNL 1 O #0 CYS 669.A CA -0.004 3.344

#1 UNL 1 C #0 ASN 507.A OD1 -0.007 3.367

#1 UNL 1 C #0 GLN 541.A CB -0.014 3.774

#1 UNL 1 C #0 LEU 544.A H -0.020 2.780

#1 UNL 1 C #0 ASN 543.A N -0.033 3.268

#1 UNL 1 C #0 MET 542.A O -0.038 3.128

#1 UNL 1 C #0 ASN 543.A O -0.043 3.283

#1 UNL 1 C #0 ASN 507.A CB -0.043 3.803

#1 UNL 1 O #0 SER 501.A CB -0.043 3.383

#1 UNL 1 O #0 PHE 504.A HE1 -0.045 2.505

#1 UNL 1 C #0 MET 542.A CA -0.048 3.538

#1 UNL 1 O #0 PHE 504.A HD2 -0.049 2.509

#1 UNL 1 C #0 MET 542.A CA -0.053 3.693

#1 UNL 1 O #0 MET 668.A SD -0.056 3.298

#1 UNL 1 C #0 MET 542.A H -0.061 2.671

#1 UNL 1 C #0 GLN 541.A CG -0.064 3.824

#1 UNL 1 C #0 ASN 543.A H -0.067 2.677

#1 UNL 1 C #0 GLN 541.A 1HE2 -0.068 2.678

#1 UNL 1 O #0 SER 501.A OG -0.083 3.043

#1 UNL 1 C #0 MET 542.A C -0.084 3.544

#1 UNL 1 C #0 ASN 507.A CA -0.087 3.847

#1 UNL 1 O #0 ASN 543.A C -0.091 3.211

#1 UNL 1 C #0 ASN 543.A 1HD2 -0.092 2.972

#1 UNL 1 C #0 GLN 541.A CD -0.094 3.404

#1 UNL 1 C #0 GLN 541.A CG -0.108 3.598

#1 UNL 1 C #0 PHE 504.A CZ -0.118 3.698

#1 UNL 1 C #0 ASN 507.A CB -0.121 3.881

#1 UNL 1 C #0 MET 542.A N -0.131 3.366

#1 UNL 1 O #0 SER 501.A OG -0.139 3.099

#1 UNL 1 O #0 MET 668.A CB -0.145 3.485

#1 UNL 1 C #0 GLN 541.A CD -0.148 3.458

#1 UNL 1 O #0 PHE 504.A CG   -0.151 3.311

#1 UNL 1 C #0 GLY 503.A N   -0.154 3.659

#1 UNL 1 O #0 MET 542.A N   -0.154 3.239

#1 UNL 1 O #0 ASN 543.A CG   -0.168 3.328

#1 UNL 1 C #0 ASN 507.A 2HD2 -0.169 3.049

#1 UNL 1 C #0 PHE 504.A CD2   -0.174 3.754

#1 UNL 1 O #0 ASN 507.A CA   -0.175 3.515

#1 UNL 1 C #0 MET 668.A CB   -0.176 3.936

#1 UNL 1 C #0 ASN 543.A ND2   -0.179 3.684

#1 UNL 1 C #0 ASN 507.A CA   -0.182 3.942

#1 UNL 1 C #0 LEU 544.A H   -0.194 2.954

#1 UNL 1 C #0 MET 542.A CA   -0.200 3.840

#1 UNL 1 C #0 ASN 543.A CB   -0.207 3.697

#1 UNL 1 O #0 GLN 541.A CA   -0.209 3.549

#1 UNL 1 C #0 ASN 543.A OD1   -0.223 3.313

#1 UNL 1 O #0 THR 540.A C   -0.227 3.387

#1 UNL 1 C #0 GLN 541.A CA   -0.230 3.720

#1 UNL 1 C #0 GLN 541.A O   -0.233 3.473

#1 UNL 1 O #0 ASN 543.A 1HD2 -0.235 2.695

#1 UNL 1 C #0 ALA 502.A H   -0.235 3.115

#1 UNL 1 C #0 GLN 541.A OE1   -0.236 3.476

#1 UNL 1 C #0 GLN 541.A 1HE2 -0.240 3.000

#1 UNL 1 C #0 PHE 504.A CE2   -0.246 3.826

#1 UNL 1 O #0 PHE 504.A CB   -0.248 3.588

#1 UNL 1 O #0 GLN 541.A CG   -0.253 3.593

#1 UNL 1 C #0 ASN 543.A C -0.261 3.571

#1 UNL 1 C #0 ASN 543.A CB -0.265 3.755

#1 UNL 1 O #0 ASN 507.A CG -0.274 3.434

#1 UNL 1 C #0 GLN 541.A N -0.274 3.659

#1 UNL 1 C #0 PHE 504.A N -0.275 3.780

#1 UNL 1 C #0 SER 501.A CA -0.286 4.046

#1 UNL 1 C #0 GLN 541.A O -0.291 3.381

#1 UNL 1 C #0 MET 668.A CB -0.293 3.933

#1 UNL 1 C #0 GLN 541.A CG -0.293 4.053

#1 UNL 1 C #0 GLN 541.A 2HE2 -0.297 2.907

#1 UNL 1 C #0 GLN 541.A N -0.308 3.813

#1 UNL 1 O #0 MET 542.A O -0.320 3.260

#1 UNL 1 O #0 GLN 541.A 2HE2 -0.320 2.780

#1 UNL 1 C #0 MET 668.A CA -0.325 3.965

#1 UNL 1 O #0 ILE 539.A CG2 -0.331 3.671

#1 UNL 1 C #0 MET 542.A CG -0.335 3.825

#1 UNL 1 C #0 PHE 504.A CA -0.336 4.096

#1 UNL 1 C #0 ASN 507.A ND2 -0.346 3.851

#1 UNL 1 C #0 GLN 541.A 1HE2 -0.350 3.230

#1 UNL 1 C #0 THR 540.A O -0.351 3.591

#1 UNL 1 C #0 GLN 541.A OE1 -0.352 3.712

#1 UNL 1 C #0 GLN 541.A O -0.356 3.596

#1 UNL 1 O #0 GLN 541.A NE2 -0.361 3.446

#1 UNL 1 O #0 PHE 504.A CB -0.362 3.702

#1 UNL 1 C #0 MET 542.A CG -0.368 3.858

#1 UNL 1 C #0 MET 542.A O -0.369 3.609

#1 UNL 1 O #0 MET 668.A C -0.369 3.529

#1 UNL 1 C #0 PHE 504.A HE1 -0.377 3.257

#1 UNL 1 C #0 LEU 544.A N -0.381 3.766

#1 UNL 1 C #0 PRO 505.A CA -0.381 4.141

#1 UNL 1 C #0 ASN 543.A N -0.385 3.620

Pharmacophore 5:

330 contacts

atom1 atom2 overlap distance

#1 UNL 1 C #0 SER 814.A C 3.139 0.441

#1 UNL 1 C #0 CYS 813.A SG 3.000 0.662

#1 UNL 1 C #0 SER 814.A CB 2.996 0.764

#1 UNL 1 C #0 SER 814.A CA 2.842 0.918

#1 UNL 1 C #0 SER 814.A CB 2.823 0.937

#1 UNL 1 C #0 CYS 813.A C 2.786 0.794

#1 UNL 1 C #0 GLN 815.A N 2.582 0.923

#1 UNL 1 C #0 SER 814.A OG 2.540 0.570

#1 UNL 1 C #0 CYS 813.A O 2.510 0.850

#1 UNL 1 C #0 PHE 812.A O 2.493 0.867

#1 UNL 1 C #0 CYS 813.A SG 2.471 1.191

#1 UNL 1 C #0 LYS 593.A CE 2.411 1.349

#1 UNL 1 C #0 SER 814.A O 2.372 0.988

#1 UNL 1 C #0 CYS 813.A CA 2.368 1.392

#1 UNL 1 C #0 CYS 813.A SG 2.342 1.320

#1 UNL 1 C #0 CYS 813.A CB 2.334 1.426

#1 UNL 1 C #0 SER 814.A C 2.300 1.280

#1 UNL 1 C #0 SER 814.A N 2.269 1.236

#1 UNL 1 C #0 SER 814.A CA 2.199 1.561

#1 UNL 1 O #0 LYS 593.A CE 2.188 1.152

#1 UNL 1 C #0 PHE 812.A C 2.186 1.394

#1 UNL 1 C #0 CYS 813.A CB 2.160 1.600

#1 UNL 1 C #0 CYS 813.A C 2.145 1.435

#1 UNL 1 C #0 SER 814.A OG 2.143 1.237

#1 UNL 1 C #0 CYS 813.A C 2.124 1.456

#1 UNL 1 C #0 CYS 813.A CB 2.111 1.649

#1 UNL 1 C #0 LYS 593.A NZ 2.089 1.416

#1 UNL 1 C #0 CYS 813.A HG 2.076 0.804

#1 UNL 1 C #0 CYS 813.A O 2.058 1.302

#1 UNL 1 C #0 CYS 813.A O 1.985 1.375

#1 UNL 1 C #0 SER 814.A CB 1.921 1.839

#1 UNL 1 C #0 SER 814.A HG 1.881 0.999

#1 UNL 1 C #0 SER 814.A CB 1.880 1.880

#1 UNL 1 C #0 SER 814.A CB 1.857 1.633

#1 UNL 1 C #0 SER 814.A C 1.849 1.731

#1 UNL 1 C #0 CYS 813.A HG 1.841 1.039

#1 UNL 1 C #0 SER 814.A CA 1.828 1.932

#1 UNL 1 C #0 GLN 815.A N 1.806 1.699

#1 UNL 1 C #0 CYS 813.A SG 1.804 1.858

#1 UNL 1 C #0 SER 814.A CA 1.787 1.973

#1 UNL 1 C #0 SER 814.A OG 1.765 1.615

#1 UNL 1 C #0 CYS 813.A SG 1.756 1.906

#1 UNL 1 C #0 GLN 815.A H 1.708 1.172

#1 UNL 1 C #0 SER 814.A HG 1.692 0.918

#1 UNL 1 C #0 SER 814.A CA 1.683 2.077

#1 UNL 1 C #0 CYS 813.A SG 1.662 2.000

#1 UNL 1 C #0 LYS 593.A HZ1 1.632 1.248

#1 UNL 1 C #0 CYS 813.A CA 1.618 2.142

#1 UNL 1 C #0 SER 814.A CB 1.558 2.202

#1 UNL 1 C #0 GLN 815.A CA 1.546 2.214

#1 UNL 1 C #0 SER 814.A CB 1.523 2.237

#1 UNL 1 C #0 CYS 813.A O 1.504 1.856

#1 UNL 1 C #0 LYS 593.A CE 1.466 2.294

#1 UNL 1 C #0 SER 814.A CA 1.452 2.308

#1 UNL 1 C #0 CYS 813.A N 1.451 2.054

#1 UNL 1 C #0 CYS 813.A N 1.444 2.061

#1 UNL 1 C #0 CYS 813.A O 1.426 1.934

#1 UNL 1 C #0 SER 814.A CB 1.417 2.343

#1 UNL 1 C #0 SER 814.A N 1.414 2.091

#1 UNL 1 C #0 SER 814.A N 1.413 2.092

#1 UNL 1 N #0 SER 814.A OG 1.399 1.741

#1 UNL 1 C #0 LYS 593.A CE 1.382 2.378

#1 UNL 1 C #0 CYS 813.A CB 1.377 2.383

#1 UNL 1 C #0 CYS 813.A CB 1.367 2.393

#1 UNL 1 C #0 SER 814.A OG 1.357 2.023

#1 UNL 1 C	#0 SER 814.A CA	1.349	2.411
#1 UNL 1 C	#0 CYS 813.A CA	1.339	2.421
#1 UNL 1 C	#0 SER 814.A CB	1.336	2.424
#1 UNL 1 C	#0 CYS 813.A SG	1.326	2.336
#1 UNL 1 C	#0 PHE 812.A O	1.312	2.048
#1 UNL 1 C	#0 CYS 813.A CA	1.295	2.465
#1 UNL 1 C	#0 CYS 813.A CB	1.295	2.465
#1 UNL 1 C	#0 PHE 812.A C	1.287	2.293
#1 UNL 1 C	#0 SER 814.A OG	1.278	2.102
#1 UNL 1 C	#0 SER 814.A O	1.255	2.105
#1 UNL 1 C	#0 CYS 813.A O	1.246	2.114
#1 UNL 1 C	#0 CYS 813.A C	1.242	2.338
#1 UNL 1 C	#0 SER 814.A N	1.215	2.290
#1 UNL 1 C	#0 CYS 813.A CA	1.133	2.627
#1 UNL 1 C	#0 SER 814.A C	1.127	2.453
#1 UNL 1 O	#0 LYS 593.A CD	1.116	2.224
#1 UNL 1 C	#0 PHE 812.A O	1.103	2.257
#1 UNL 1 C	#0 GLN 815.A N	1.093	2.412
#1 UNL 1 O	#0 CYS 813.A HG	1.088	1.372
#1 UNL 1 C	#0 CYS 813.A C	1.064	2.516
#1 UNL 1 C	#0 CYS 813.A SG	1.062	2.600
#1 UNL 1 C	#0 GLN 815.A CA	1.060	2.700
#1 UNL 1 C	#0 SER 814.A N	1.059	2.446
#1 UNL 1 C	#0 GLN 815.A CG	1.053	2.707
#1 UNL 1 C	#0 PHE 812.A HD2	1.035	1.845

#1	UNL	1	C	#0	PHE 812.A	CD2	1.028	2.552
#1	UNL	1	C	#0	SER 814.A	N	1.023	2.482
#1	UNL	1	C	#0	PHE 812.A	CA	1.020	2.740
#1	UNL	1	C	#0	PHE 812.A	C	1.011	2.569
#1	UNL	1	C	#0	SER 814.A	O	0.996	2.364
#1	UNL	1	C	#0	CYS 813.A	C	0.981	2.599
#1	UNL	1	C	#0	GLN 815.A	CB	0.955	2.805
#1	UNL	1	C	#0	CYS 813.A	O	0.954	2.406
#1	UNL	1	C	#0	LYS 593.A	CG	0.953	2.807
#1	UNL	1	O	#0	LYS 593.A	NZ	0.944	2.141
#1	UNL	1	C	#0	CYS 813.A	CA	0.942	2.818
#1	UNL	1	C	#0	SER 814.A	OG	0.939	2.441
#1	UNL	1	C	#0	CYS 813.A	O	0.937	2.423
#1	UNL	1	C	#0	CYS 813.A	HG	0.927	1.953
#1	UNL	1	C	#0	SER 814.A	CB	0.916	2.844
#1	UNL	1	C	#0	CYS 813.A	C	0.907	2.673
#1	UNL	1	C	#0	SER 814.A	HG	0.894	1.986
#1	UNL	1	C	#0	LYS 593.A	CD	0.872	2.888
#1	UNL	1	C	#0	CYS 813.A	HG	0.866	2.014
#1	UNL	1	C	#0	SER 814.A	N	0.844	2.661
#1	UNL	1	C	#0	CYS 813.A	C	0.819	2.761
#1	UNL	1	C	#0	SER 814.A	OG	0.803	2.577
#1	UNL	1	C	#0	SER 814.A	H	0.799	2.081
#1	UNL	1	C	#0	SER 814.A	CA	0.792	2.698
#1	UNL	1	C	#0	GLN 815.A	H	0.791	2.089

#1 UNL 1 C #0 CYS 813.A HG 0.789 2.091

#1 UNL 1 C #0 SER 814.A O 0.777 2.583

#1 UNL 1 C #0 SER 814.A C 0.775 2.805

#1 UNL 1 C #0 SER 814.A C 0.761 2.819

#1 UNL 1 C #0 GLN 815.A CG 0.759 3.001

#1 UNL 1 N #0 SER 814.A CB 0.755 2.765

#1 UNL 1 C #0 CYS 813.A CB 0.750 3.010

#1 UNL 1 C #0 GLN 815.A CD 0.747 2.833

#1 UNL 1 C #0 CYS 813.A N 0.742 2.763

#1 UNL 1 C #0 LYS 593.A NZ 0.742 2.763

#1 UNL 1 C #0 LYS 593.A CE 0.731 3.029

#1 UNL 1 N #0 SER 814.A HG 0.726 1.914

#1 UNL 1 C #0 SER 814.A CB 0.726 3.034

#1 UNL 1 C #0 PHE 812.A O 0.720 2.640

#1 UNL 1 C #0 CYS 813.A CB 0.716 3.044

#1 UNL 1 O #0 CYS 813.A SG 0.707 2.535

#1 UNL 1 C #0 PHE 812.A HE2 0.702 2.178

#1 UNL 1 C #0 LYS 593.A HZ2 0.698 2.182

#1 UNL 1 C #0 GLN 815.A OE1 0.679 2.681

#1 UNL 1 C #0 LYS 593.A HZ3 0.669 2.211

#1 UNL 1 C #0 SER 814.A CA 0.662 3.098

#1 UNL 1 C #0 PHE 812.A O 0.649 2.711

#1 UNL 1 C #0 CYS 813.A C 0.636 2.944

#1 UNL 1 C #0 CYS 813.A SG 0.598 3.064

#1 UNL 1 C #0 CYS 813.A CA 0.598 3.162

#1 UNL 1 C #0 PHE 812.A C 0.592 2.988

#1 UNL 1 C #0 SER 814.A HG 0.589 2.291

#1 UNL 1 C #0 LEU 758.A CD2 0.587 3.173

#1 UNL 1 C #0 CYS 813.A CB 0.579 3.181

#1 UNL 1 C #0 SER 814.A C 0.578 3.002

#1 UNL 1 C #0 SER 814.A HG 0.562 2.318

#1 UNL 1 C #0 GLN 815.A N 0.555 2.950

#1 UNL 1 C #0 ARG 836.A CZ 0.553 3.027

#1 UNL 1 C #0 CYS 813.A O 0.537 2.823

#1 UNL 1 C #0 CYS 813.A HG 0.532 2.348

#1 UNL 1 O #0 SER 814.A OG 0.525 2.435

#1 UNL 1 C #0 PHE 812.A CB 0.524 3.236

#1 UNL 1 O #0 CYS 813.A O 0.511 2.429

#1 UNL 1 C #0 SER 814.A CA 0.508 3.252

#1 UNL 1 C #0 TRP 598.A HZ3 0.503 2.377

#1 UNL 1 C #0 SER 814.A OG 0.501 2.879

#1 UNL 1 C #0 PHE 812.A CE2 0.493 3.087

#1 UNL 1 C #0 CYS 813.A N 0.483 3.022

#1 UNL 1 O #0 CYS 813.A CB 0.474 2.866

#1 UNL 1 C #0 PHE 812.A HE2 0.473 2.407

#1 UNL 1 C #0 CYS 813.A C 0.473 3.107

#1 UNL 1 C #0 LYS 593.A HZ1 0.470 2.410

#1 UNL 1 O #0 CYS 813.A HG 0.460 2.000

#1 UNL 1 C #0 SER 814.A CB 0.446 3.314

#1 UNL 1 C #0 CYS 813.A C 0.443 3.137

#1 UNL 1 C #0 LYS 593.A CD   0.440 3.320

#1 UNL 1 O #0 LYS 593.A CG   0.429 2.911

#1 UNL 1 O #0 LYS 593.A CE   0.425 2.915

#1 UNL 1 C #0 GLN 815.A H   0.421 2.459

#1 UNL 1 C #0 GLN 815.A H   0.415 2.465

#1 UNL 1 C #0 LYS 593.A CD   0.412 3.348

#1 UNL 1 C #0 CYS 813.A HG   0.402 2.478

#1 UNL 1 C #0 GLN 815.A CD   0.399 3.181

#1 UNL 1 C #0 ARG 836.A NH2   0.373 3.132

#1 UNL 1 C #0 GLN 815.A OE1   0.366 2.994

#1 UNL 1 C #0 CYS 813.A SG   0.360 3.302

#1 UNL 1 C #0 GLN 815.A CG   0.352 3.408

#1 UNL 1 O #0 CYS 813.A SG   0.343 2.899

#1 UNL 1 O #0 SER 814.A OG   0.338 2.622

#1 UNL 1 C #0 CYS 813.A C   0.329 3.251

#1 UNL 1 C #0 SER 814.A CA   0.320 3.440

#1 UNL 1 C #0 CYS 813.A HG   0.306 2.574

#1 UNL 1 C #0 LYS 593.A CG   0.302 3.458

#1 UNL 1 C #0 TRP 598.A HZ3   0.302 2.578

#1 UNL 1 C #0 GLN 815.A OE1   0.297 3.063

#1 UNL 1 C #0 CYS 813.A CB   0.291 3.469

#1 UNL 1 C #0 CYS 813.A CA   0.284 3.476

#1 UNL 1 O #0 TRP 598.A HZ3   0.280 2.180

#1 UNL 1 C #0 GLN 815.A NE2   0.268 3.237

#1 UNL 1 C #0 GLN 815.A C   0.265 3.315

#1 UNL 1 C #0 TRP 598.A CZ3 0.263 3.317

#1 UNL 1 C #0 SER 814.A N 0.262 2.973

#1 UNL 1 C #0 PHE 812.A CG 0.261 3.319

#1 UNL 1 C #0 PHE 812.A O 0.261 3.099

#1 UNL 1 C #0 PHE 812.A CB 0.245 3.515

#1 UNL 1 C #0 PHE 812.A CE2 0.235 3.345

#1 UNL 1 C #0 PHE 812.A CD2 0.215 3.365

#1 UNL 1 C #0 GLN 815.A H 0.214 2.666

#1 UNL 1 C #0 CYS 813.A O 0.213 3.147

#1 UNL 1 C #0 SER 814.A N 0.209 3.296

#1 UNL 1 C #0 LYS 593.A CE 0.209 3.551

#1 UNL 1 C #0 PHE 812.A CE2 0.209 3.371

#1 UNL 1 C #0 CYS 813.A CA 0.202 3.558

#1 UNL 1 C #0 CYS 813.A CA 0.193 3.567

#1 UNL 1 C #0 GLN 815.A CG 0.191 3.569

#1 UNL 1 C #0 GLN 815.A N 0.189 3.316

#1 UNL 1 C #0 PHE 812.A O 0.186 3.174

#1 UNL 1 C #0 PHE 812.A CD2 0.183 3.397

#1 UNL 1 C #0 GLN 815.A CD 0.179 3.401

#1 UNL 1 C #0 CYS 813.A SG 0.172 3.490

#1 UNL 1 C #0 GLN 815.A CG 0.170 3.590

#1 UNL 1 C #0 SER 814.A CA 0.169 3.591

#1 UNL 1 C #0 SER 814.A O 0.169 3.191

#1 UNL 1 C #0 CYS 813.A O 0.166 3.194

#1 UNL 1 O #0 SER 814.A CB 0.166 3.174

#1 UNL 1 C #0 GLN 815.A N 0.157 3.348

#1 UNL 1 C #0 TRP 598.A CZ3 0.155 3.425

#1 UNL 1 C #0 GLN 815.A CB 0.142 3.618

#1 UNL 1 O #0 TRP 598.A CZ3 0.141 3.019

#1 UNL 1 C #0 PHE 812.A CA 0.139 3.621

#1 UNL 1 O #0 SER 814.A CA 0.107 3.233

#1 UNL 1 C #0 SER 814.A CA 0.106 3.654

#1 UNL 1 C #0 PHE 812.A C 0.101 3.479

#1 UNL 1 C #0 SER 814.A H 0.098 2.782

#1 UNL 1 C #0 CYS 813.A N 0.093 3.412

#1 UNL 1 O #0 SER 814.A HG 0.091 2.369

#1 UNL 1 C #0 CYS 813.A CA 0.088 3.672

#1 UNL 1 C #0 CYS 813.A O 0.088 3.272

#1 UNL 1 C #0 GLN 815.A CA 0.084 3.676

#1 UNL 1 C #0 SER 814.A HG 0.081 2.799

#1 UNL 1 C #0 LYS 593.A NZ 0.080 3.425

#1 UNL 1 C #0 CYS 813.A CB 0.079 3.681

#1 UNL 1 C #0 GLN 815.A CB 0.071 3.689

#1 UNL 1 C #0 GLN 815.A CG 0.069 3.691

#1 UNL 1 C #0 PHE 812.A N 0.067 3.438

#1 UNL 1 N #0 SER 814.A N 0.067 3.198

#1 UNL 1 C #0 LYS 593.A CD 0.065 3.695

#1 UNL 1 N #0 CYS 813.A C 0.059 3.281

#1 UNL 1 C #0 ARG 836.A NH1 0.052 3.453

#1 UNL 1 C #0 PHE 812.A HD2 0.050 2.830

#1 UNL 1 C #0 CYS 813.A SG 0.044 3.618

#1 UNL 1 C #0 GLN 815.A N 0.040 3.465

#1 UNL 1 N #0 SER 814.A CA 0.034 3.486

#1 UNL 1 C #0 CYS 813.A O 0.031 3.329

#1 UNL 1 C #0 ARG 836.A NE 0.028 3.477

#1 UNL 1 C #0 SER 814.A HG 0.027 2.853

#1 UNL 1 C #0 PHE 812.A CG 0.021 3.559

#1 UNL 1 C #0 SER 814.A N 0.018 3.487

#1 UNL 1 C #0 GLN 815.A CB 0.011 3.749

#1 UNL 1 C #0 GLN 815.A OE1 0.005 3.355

#1 UNL 1 C #0 GLN 815.A NE2 0.004 3.501

#1 UNL 1 O #0 LYS 593.A HZ1 -0.006 2.466

#1 UNL 1 C #0 GLN 815.A CD -0.010 3.590

#1 UNL 1 C #0 GLN 815.A CG -0.021 3.781

#1 UNL 1 C #0 CYS 813.A C -0.021 3.331

#1 UNL 1 C #0 SER 814.A H -0.030 2.910

#1 UNL 1 C #0 PHE 812.A C -0.037 3.617

#1 UNL 1 C #0 MET 601.A SD -0.039 3.701

#1 UNL 1 C #0 SER 814.A H -0.047 2.927

#1 UNL 1 C #0 PHE 812.A CA -0.048 3.808

#1 UNL 1 O #0 LYS 593.A HZ2 -0.068 2.528

#1 UNL 1 C #0 PHE 812.A HD2 -0.076 2.956

#1 UNL 1 C #0 SER 814.A C -0.077 3.657

#1 UNL 1 C #0 GLN 815.A CB -0.088 3.848

#1 UNL 1 C #0 SER 814.A C -0.107 3.687

#1 UNL 1 C #0 PHE 812.A C -0.114 3.694

#1 UNL 1 C #0 CYS 813.A H -0.122 3.002

#1 UNL 1 C #0 GLN 815.A CB -0.130 3.890

#1 UNL 1 C #0 CYS 813.A H -0.135 3.015

#1 UNL 1 C #0 LYS 593.A CG -0.140 3.900

#1 UNL 1 C #0 ARG 836.A NH2 -0.154 3.659

#1 UNL 1 C #0 GLN 815.A CG -0.158 3.918

#1 UNL 1 C #0 SER 814.A H -0.160 3.040

#1 UNL 1 O #0 TRP 598.A CE3 -0.162 3.322

#1 UNL 1 C #0 CYS 813.A SG -0.162 3.824

#1 UNL 1 C #0 LYS 593.A CE -0.174 3.934

#1 UNL 1 C #0 CYS 813.A O -0.179 3.539

#1 UNL 1 C #0 TRP 598.A CE3 -0.180 3.760

#1 UNL 1 C #0 GLN 815.A CD -0.182 3.762

#1 UNL 1 C #0 SER 814.A O -0.183 3.543

#1 UNL 1 O #0 LEU 758.A CD2 -0.187 3.527

#1 UNL 1 C #0 SER 814.A N -0.189 3.694

#1 UNL 1 O #0 GLN 815.A OE1 -0.190 3.130

#1 UNL 1 C #0 GLN 815.A O -0.190 3.550

#1 UNL 1 C #0 SER 814.A OG -0.192 3.572

#1 UNL 1 C #0 SER 814.A O -0.194 3.554

#1 UNL 1 C #0 CYS 813.A C -0.198 3.778

#1 UNL 1 C #0 CYS 813.A HG -0.209 3.089

#1 UNL 1 C #0 CYS 813.A HG -0.210 3.090

#1 UNL 1 C #0 TRP 598.A HZ3 -0.217 3.097

#1 UNL 1 O #0 ARG 836.A NH1 -0.221 3.306

#1 UNL 1 C #0 SER 814.A OG -0.224 3.604

#1 UNL 1 C #0 SER 814.A C -0.225 3.805

#1 UNL 1 C #0 TRP 598.A CH2 -0.226 3.806

#1 UNL 1 C #0 CYS 813.A HG -0.227 3.107

#1 UNL 1 C #0 GLN 815.A C -0.227 3.807

#1 UNL 1 C #0 GLN 815.A CB -0.232 3.992

#1 UNL 1 C #0 GLN 815.A CA -0.240 4.000

#1 UNL 1 C #0 SER 814.A N -0.246 3.751

#1 UNL 1 C #0 MET 601.A CE -0.249 4.009

#1 UNL 1 C #0 CYS 813.A C -0.249 3.829

#1 UNL 1 C #0 GLN 815.A OE1 -0.250 3.610

#1 UNL 1 C #0 TRP 598.A HZ3 -0.262 3.142

#1 UNL 1 C #0 CYS 813.A SG -0.270 3.932

#1 UNL 1 O #0 ARG 836.A CD -0.273 3.613

#1 UNL 1 C #0 PHE 812.A H -0.277 3.157

#1 UNL 1 C #0 CYS 813.A N -0.280 3.785

#1 UNL 1 C #0 GLN 815.A N -0.297 3.802

#1 UNL 1 C #0 PHE 812.A HD2 -0.298 3.178

#1 UNL 1 C #0 GLN 815.A CG -0.302 4.062

#1 UNL 1 C #0 GLN 815.A 1HE2 -0.318 3.198

#1 UNL 1 C #0 CYS 813.A CB -0.319 4.079

#1 UNL 1 C #0 CYS 813.A CB -0.319 4.079

#1 UNL 1 C #0 CYS 813.A N -0.327 3.832

#1 UNL 1 O #0 SER 814.A CB -0.329 3.669

#1 UNL 1 C #0 CYS 813.A CA -0.330 4.090

#1 UNL 1 C #0 GLN 815.A OE1 -0.337 3.697

#1 UNL 1 O #0 SER 814.A OG -0.342 3.302

#1 UNL 1 C #0 TRP 598.A CZ3 -0.348 3.928

#1 UNL 1 C #0 ARG 836.A CZ -0.350 3.930

#1 UNL 1 C #0 GLN 815.A CB -0.350 4.110

#1 UNL 1 O #0 CYS 813.A C -0.356 3.516

#1 UNL 1 C #0 PHE 812.A CD2 -0.357 3.937

#1 UNL 1 C #0 CYS 813.A O -0.362 3.452

#1 UNL 1 O #0 TRP 598.A HE3 -0.367 2.827

#1 UNL 1 C #0 SER 814.A O -0.374 3.734

#1 UNL 1 O #0 ARG 836.A CZ -0.374 3.534

#1 UNL 1 C #0 LYS 593.A HZ3 -0.385 3.265

#1 UNL 1 C #0 CYS 813.A SG -0.390 4.052

#1 UNL 1 C #0 CYS 813.A C -0.398 3.978

Pharmacophore 6:

118 contacts

atom1 atom2 overlap distance

#1 UNL 1 C #0 GLU 811.A CD 3.174 0.406

#1 UNL 1 N #0 GLU 811.A OE2 2.555 0.565

#1 UNL 1 O #0 TRP 800.A CD1 2.479 0.641

#1 UNL 1 C #0 HIS 810.A CE1 2.308 1.332

#1 UNL 1 C #0 GLU 811.A OE2 2.295 1.065

#1 UNL 1 C #0 GLU 811.A OE1 2.169 1.191

#1	UNL 1	C	#0	GLU 811.A	CD	1.900	1.410
#1	UNL 1	C	#0	GLU 811.A	OE1	1.899	1.191
#1	UNL 1	C	#0	GLU 811.A	CG	1.850	1.910
#1	UNL 1	C	#0	GLU 811.A	OE2	1.828	1.532
#1	UNL 1	O	#0	TRP 800.A	HD1	1.787	0.633
#1	UNL 1	C	#0	HIS 810.A	ND1	1.783	1.722
#1	UNL 1	C	#0	GLU 811.A	OE1	1.732	1.628
#1	UNL 1	N	#0	GLU 811.A	CD	1.707	1.633
#1	UNL 1	C	#0	TRP 800.A	CD1	1.684	1.626
#1	UNL 1	C	#0	GLU 811.A	CD	1.674	1.906
#1	UNL 1	C	#0	TRP 800.A	HD1	1.562	1.048
#1	UNL 1	O	#0	GLU 811.A	CG	1.549	1.751
#1	UNL 1	C	#0	GLU 811.A	OE2	1.411	1.949
#1	UNL 1	O	#0	TRP 800.A	CG	1.396	1.724
#1	UNL 1	C	#0	GLU 811.A	OE2	1.363	1.997
#1	UNL 1	C	#0	GLU 811.A	OE1	1.346	1.744
#1	UNL 1	C	#0	GLU 811.A	CG	1.303	2.187
#1	UNL 1	O	#0	TRP 800.A	NE1	1.289	1.756
#1	UNL 1	C	#0	GLU 811.A	OE1	1.282	2.078
#1	UNL 1	O	#0	GLU 811.A	CB	1.238	2.062
#1	UNL 1	C	#0	GLU 811.A	CD	1.190	2.390
#1	UNL 1	C	#0	TRP 800.A	NE1	1.164	2.071
#1	UNL 1	O	#0	GLU 811.A	CD	1.144	1.976
#1	UNL 1	C	#0	GLU 811.A	CG	1.096	2.664
#1	UNL 1	C	#0	GLU 811.A	OE1	1.023	2.337

#1 UNL 1 C   #0 GLU 811.A CD   0.973 2.607

#1 UNL 1 C   #0 TRP 800.A NE1 0.959 2.276

#1 UNL 1 N   #0 GLU 811.A CG   0.931 2.589

#1 UNL 1 C   #0 HIS 810.A NE2 0.921 2.599

#1 UNL 1 C   #0 TRP 800.A HE1 0.888 1.722

#1 UNL 1 C   #0 HIS 816.A CE1 0.859 2.781

#1 UNL 1 N   #0 TRP 800.A HD1 0.847 1.793

#1 UNL 1 C   #0 GLU 811.A OE1 0.837 2.253

#1 UNL 1 C   #0 GLU 811.A CD   0.817 2.763

#1 UNL 1 C   #0 GLU 811.A CB   0.811 2.949

#1 UNL 1 C   #0 GLU 811.A CB   0.803 2.687

#1 UNL 1 C   #0 GLU 811.A CD   0.761 2.549

#1 UNL 1 C   #0 GLU 811.A OE2 0.746 2.344

#1 UNL 1 C   #0 TRP 800.A HE1 0.739 1.871

#1 UNL 1 C   #0 TRP 800.A CD1 0.711 2.599

#1 UNL 1 O   #0 GLU 811.A OE1 0.685 2.215

#1 UNL 1 N   #0 HIS 810.A CE1 0.667 2.733

#1 UNL 1 O   #0 TRP 800.A CB   0.659 2.641

#1 UNL 1 C   #0 GLU 811.A CD   0.658 2.922

#1 UNL 1 C   #0 HIS 810.A CG   0.633 2.947

#1 UNL 1 N   #0 TRP 800.A CD1 0.630 2.710

#1 UNL 1 O   #0 HIS 810.A ND1 0.626 2.419

#1 UNL 1 C   #0 TRP 800.A HE1 0.577 2.033

#1 UNL 1 C   #0 GLU 811.A CG   0.552 3.208

#1 UNL 1 N   #0 GLU 811.A OE1 0.493 2.627

#1 UNL 1 C #0 GLU 811.A OE2 0.490 2.870

#1 UNL 1 C #0 GLU 811.A CG 0.486 3.274

#1 UNL 1 C #0 HIS 816.A NE2 0.457 3.063

#1 UNL 1 C #0 TRP 800.A NE1 0.404 2.831

#1 UNL 1 C #0 TRP 800.A CG 0.404 2.906

#1 UNL 1 O #0 TRP 800.A CE2 0.393 2.727

#1 UNL 1 O #0 TRP 800.A CD2 0.385 2.735

#1 UNL 1 C #0 HIS 816.A CE1 0.336 3.304

#1 UNL 1 C #0 HIS 810.A CD2 0.317 3.323

#1 UNL 1 O #0 TRP 800.A HE1 0.271 2.149

#1 UNL 1 C #0 TRP 800.A NE1 0.206 3.029

#1 UNL 1 O #0 GLU 811.A OE1 0.194 2.746

#1 UNL 1 N #0 HIS 810.A ND1 0.180 3.085

#1 UNL 1 O #0 GLU 811.A CD 0.175 2.985

#1 UNL 1 C #0 HIS 810.A ND1 0.173 3.062

#1 UNL 1 O #0 GLU 811.A CA 0.170 3.130

#1 UNL 1 C #0 TRP 800.A HD1 0.152 2.458

#1 UNL 1 C #0 GLU 811.A CD 0.144 3.166

#1 UNL 1 O #0 TRP 800.A HE1 0.122 2.298

#1 UNL 1 O #0 GLU 811.A OE2 0.091 2.849

#1 UNL 1 C #0 GLU 811.A CG 0.059 3.701

#1 UNL 1 C #0 GLU 811.A OE2 0.055 3.305

#1 UNL 1 N #0 LYS 798.A O 0.049 3.071

#1 UNL 1 Cl #0 SER 814.A OG 0.035 3.445

#1 UNL 1 C #0 TRP 800.A CD1 0.027 3.283

#1 UNL 1 C #0 GLU 811.A OE1 0.022 3.068

#1 UNL 1 N #0 TRP 800.A NE1 0.015 3.250

#1 UNL 1 O #0 TRP 800.A NE1 0.003 3.042

#1 UNL 1 C #0 HIS 810.A CE1 -0.014 3.654

#1 UNL 1 C #0 GLU 811.A CG -0.028 3.518

#1 UNL 1 C #0 HIS 810.A ND1 -0.036 3.541

#1 UNL 1 O #0 GLU 811.A OE2 -0.037 2.937

#1 UNL 1 C #0 TRP 800.A CE2 -0.040 3.350

#1 UNL 1 O #0 GLU 811.A CG -0.042 3.382

#1 UNL 1 C #0 GLU 811.A OE1 -0.062 3.422

#1 UNL 1 C #0 HIS 810.A CE1 -0.068 3.708

#1 UNL 1 O #0 HIS 810.A C -0.074 3.194

#1 UNL 1 O #0 GLU 811.A N -0.079 3.124

#1 UNL 1 C #0 TRP 800.A HE1 -0.082 2.692

#1 UNL 1 O #0 GLU 811.A CB -0.102 3.442

#1 UNL 1 C #0 TRP 800.A HE1 -0.106 2.986

#1 UNL 1 N #0 HIS 816.A CE1 -0.109 3.509

#1 UNL 1 C #0 GLU 811.A CB -0.118 3.878

#1 UNL 1 C #0 HIS 810.A O -0.135 3.495

#1 UNL 1 C #0 GLU 811.A OE1 -0.153 3.513

#1 UNL 1 C #0 TRP 800.A CE2 -0.168 3.478

#1 UNL 1 C #0 GLU 811.A OE2 -0.174 3.264

#1 UNL 1 O #0 HIS 810.A CE1 -0.191 3.371

#1 UNL 1 O #0 GLU 811.A CD -0.235 3.395

#1 UNL 1 C #0 GLU 811.A CB -0.247 4.007

#1 UNL 1 O #0 HIS 810.A CG -0.250 3.370

#1 UNL 1 C #0 TRP 800.A CD1 -0.250 3.560

#1 UNL 1 C #0 HIS 810.A CE1 -0.252 3.622

#1 UNL 1 C #0 GLU 811.A CB -0.270 3.760

#1 UNL 1 C #0 GLU 811.A OE1 -0.294 3.654

#1 UNL 1 C #0 GLU 811.A CB -0.295 4.055

#1 UNL 1 O #0 GLU 811.A OE1 -0.315 3.255

#1 UNL 1 C #0 TRP 800.A CB -0.319 3.809

#1 UNL 1 O #0 TRP 800.A CA -0.329 3.629

#1 UNL 1 C #0 HIS 810.A HD1 -0.359 3.239

#1 UNL 1 O #0 TRP 800.A CD1 -0.363 3.483

#1 UNL 1 O #0 HIS 810.A CB -0.366 3.666

Pharmacophore 7:

354 contacts

atom1 atom2 overlap distance

#1 UNL 1 C #0 ARG 553.A CZ 2.534 1.046

#1 UNL 1 C #0 ARG 553.A NH2 2.449 0.786

#1 UNL 1 C #0 ARG 553.A NH1 2.299 1.206

#1 UNL 1 C #0 ARG 553.A NH2 2.268 1.237

#1 UNL 1 O #0 TYR 455.A CD2 2.250 0.870

#1 UNL 1 C #0 ARG 553.A CG 2.197 1.563

#1 UNL 1 C #0 ARG 553.A 2HH2 2.129 0.481

#1 UNL 1 C #0 ARG 553.A CZ 2.094 1.486

#1 UNL 1 O #0 ARG 555.A CA 2.051 1.249

#1 UNL 1 C #0 ARG 555.A N 2.044 1.191

#1 UNL 1 C #0 ARG 555.A CA 1.988 1.502

#1 UNL 1 C #0 ARG 553.A NH1 1.986 1.519

#1 UNL 1 N #0 TYR 455.A CB 1.983 1.537

#1 UNL 1 O #0 TYR 455.A CG 1.952 1.168

#1 UNL 1 C #0 ARG 553.A NH1 1.942 1.563

#1 UNL 1 C #0 ARG 553.A 1HH2 1.929 0.951

#1 UNL 1 C #0 ARG 553.A CB 1.837 1.923

#1 UNL 1 C #0 ARG 553.A CD 1.837 1.923

#1 UNL 1 C #0 ARG 553.A NH2 1.835 1.400

#1 UNL 1 O #0 ARG 555.A CA 1.825 1.515

#1 UNL 1 C #0 ARG 553.A NH1 1.824 1.681

#1 UNL 1 C #0 ARG 555.A CA 1.786 1.704

#1 UNL 1 O #0 ASP 452.A OD1 1.771 1.129

#1 UNL 1 C #0 ARG 555.A CA 1.764 1.726

#1 UNL 1 C #0 ARG 555.A CB 1.734 1.756

#1 UNL 1 C #0 ARG 553.A NH2 1.730 1.775

#1 UNL 1 C #0 ARG 553.A 2HH1 1.723 1.157

#1 UNL 1 C #0 ARG 555.A N 1.687 1.548

#1 UNL 1 C #0 TYR 455.A CG 1.671 1.639

#1 UNL 1 O #0 ARG 555.A N 1.657 1.428

#1 UNL 1 C #0 ARG 555.A H 1.557 1.053

#1 UNL 1 C #0 ARG 553.A NH1 1.540 1.695

#1 UNL 1 N #0 ARG 624.A NE 1.539 1.726

#1 UNL 1 N #0 ARG 624.A CD 1.530 1.990

#1 UNL 1 O #0 TYR 455.A CE2 1.529 1.591

#1 UNL 1 C #0 ARG 553.A NE 1.525 1.980

#1 UNL 1 C #0 ARG 553.A 2HH2 1.522 1.088

#1 UNL 1 C #0 ARG 553.A NH1 1.518 1.717

#1 UNL 1 C #0 ARG 553.A 1HH1 1.509 1.371

#1 UNL 1 O #0 ARG 555.A C 1.506 1.614

#1 UNL 1 C #0 ARG 553.A 1HH2 1.503 1.107

#1 UNL 1 C #0 ARG 553.A CZ 1.478 1.832

#1 UNL 1 C #0 ARG 553.A CZ 1.464 2.116

#1 UNL 1 O #0 ARG 555.A CB 1.458 1.882

#1 UNL 1 C #0 ARG 553.A 1HH1 1.458 1.152

#1 UNL 1 C #0 TYR 455.A CD2 1.439 1.871

#1 UNL 1 N #0 TYR 455.A CG 1.374 1.966

#1 UNL 1 C #0 ARG 555.A CB 1.351 2.139

#1 UNL 1 C #0 ARG 553.A 2HH1 1.347 1.533

#1 UNL 1 C #0 ARG 553.A NH2 1.337 2.168

#1 UNL 1 C #0 ARG 553.A NH2 1.314 1.921

#1 UNL 1 C #0 ALA 554.A C 1.309 2.001

#1 UNL 1 O #0 ARG 555.A H 1.283 1.177

#1 UNL 1 C #0 ALA 554.A O 1.255 1.835

#1 UNL 1 O #0 ARG 555.A C 1.249 1.911

#1 UNL 1 O #0 TYR 455.A CD1 1.247 1.873

#1 UNL 1 C #0 ARG 553.A 1HH1 1.243 1.367

#1 UNL 1 O #0 THR 556.A N 1.241 1.804

#1 UNL 1 C #0 ARG 555.A N 1.237 1.998

#1 UNL 1 C #0 TYR 455.A CB 1.215 2.275

#1 UNL 1 C #0 ARG 553.A 2HH1 1.195 1.685

#1 UNL 1 O #0 ARG 553.A 1HH2 1.195 1.265

#1 UNL 1 C #0 ARG 553.A CZ 1.192 2.388

#1 UNL 1 C #0 ALA 554.A C 1.185 2.125

#1 UNL 1 C #0 ARG 553.A 1HH1 1.132 1.748

#1 UNL 1 N #0 ARG 624.A CZ 1.130 2.210

#1 UNL 1 C #0 ARG 553.A 1HH1 1.111 1.769

#1 UNL 1 C #0 ARG 624.A 2HH2 1.109 1.501

#1 UNL 1 C #0 ARG 553.A 1HH1 1.101 1.779

#1 UNL 1 C #0 ARG 553.A 2HH2 1.067 1.543

#1 UNL 1 O #0 ARG 624.A 2HH2 1.062 1.358

#1 UNL 1 O #0 ARG 555.A O 1.053 1.887

#1 UNL 1 O #0 ARG 553.A NH2 1.046 1.999

#1 UNL 1 C #0 ARG 553.A O 1.010 2.230

#1 UNL 1 C #0 ARG 553.A 2HH1 1.007 1.873

#1 UNL 1 C #0 ARG 555.A H 1.006 1.754

#1 UNL 1 C #0 ARG 555.A N 0.998 2.387

#1 UNL 1 C #0 ARG 553.A NE 0.993 2.512

#1 UNL 1 C #0 ARG 555.A CB 0.989 2.651

#1 UNL 1 C #0 ALA 554.A C 0.986 2.324

#1 UNL 1 O #0 ARG 553.A NH2 0.972 2.113

#1 UNL 1 C #0 ARG 624.A NE 0.965 2.270

#1 UNL 1 C #0 ARG 624.A HE 0.964 1.646

#1 UNL 1 C #0 TYR 455.A CD1 0.962 2.348

#1 UNL 1 C #0 ARG 553.A 2HH1 0.954 1.926

#1 UNL 1 C #0 ARG 553.A CZ 0.947 2.363

#1 UNL 1 O #0 TYR 455.A CZ 0.943 2.177

#1 UNL 1 C #0 ARG 624.A NE 0.932 2.303

#1 UNL 1 O #0 ARG 624.A NH2 0.930 2.115

#1 UNL 1 O #0 ASP 452.A CG 0.928 2.192

#1 UNL 1 C #0 ARG 553.A O 0.927 2.313

#1 UNL 1 C #0 ARG 553.A CD 0.923 2.837

#1 UNL 1 C #0 ALA 554.A O 0.911 2.179

#1 UNL 1 O #0 ARG 555.A N 0.903 2.142

#1 UNL 1 C #0 ARG 555.A CB 0.899 2.591

#1 UNL 1 C #0 ASP 452.A OD1 0.884 2.206

#1 UNL 1 C #0 ARG 553.A CZ 0.878 2.432

#1 UNL 1 C #0 ARG 553.A NH1 0.855 2.650

#1 UNL 1 O #0 TYR 455.A CE1 0.836 2.284

#1 UNL 1 C #0 ARG 624.A NH2 0.829 2.406

#1 UNL 1 O #0 TYR 455.A CB 0.825 2.475

#1 UNL 1 C #0 ARG 553.A 2HH1 0.804 1.806

#1 UNL 1 C #0 ARG 553.A CZ 0.800 2.510

#1 UNL 1 C #0 ARG 553.A 2HH2 0.800 1.810

#1 UNL 1 C #0 ARG 624.A CZ 0.793 2.517

#1 UNL 1 C #0 ARG 553.A CA 0.788 2.972

#1 UNL 1 O #0 TYR 455.A HD2 0.768 1.652

#1 UNL 1 C #0 ARG 553.A NH1 0.748 2.757

#1 UNL 1 C #0 ARG 553.A NH1 0.730 2.775

#1 UNL 1 O #0 ASP 452.A OD1 0.724 2.216

#1 UNL 1 C #0 ARG 553.A 1HH2 0.711 2.169

#1 UNL 1 O #0 ASP 452.A CG 0.705 2.455

#1 UNL 1 C #0 ALA 554.A O 0.689 2.401

#1 UNL 1 C #0 ARG 553.A NE 0.688 2.817

#1 UNL 1 O #0 THR 556.A H 0.682 1.738

#1 UNL 1 C #0 ARG 553.A CG 0.681 3.079

#1 UNL 1 C #0 ARG 555.A C 0.659 2.651

#1 UNL 1 C #0 ARG 553.A NH2 0.657 2.578

#1 UNL 1 C #0 ARG 553.A 2HH2 0.656 2.224

#1 UNL 1 O #0 ARG 555.A CB 0.653 2.647

#1 UNL 1 O #0 ASP 452.A OD2 0.648 2.292

#1 UNL 1 C #0 ARG 553.A CD 0.645 3.115

#1 UNL 1 N #0 TYR 455.A CD1 0.618 2.722

#1 UNL 1 C #0 ARG 553.A CZ 0.618 2.962

#1 UNL 1 C #0 ARG 553.A NE 0.618 2.887

#1 UNL 1 O #0 ALA 554.A C 0.604 2.516

#1 UNL 1 N #0 TYR 455.A CD2 0.604 2.736

#1 UNL 1 C #0 ARG 624.A HE 0.602 2.008

#1 UNL 1 C #0 ARG 553.A 1HH2 0.588 2.022

#1 UNL 1 C #0 ALA 554.A O 0.584 2.506

#1 UNL 1 C #0 TYR 455.A CE2 0.570 2.740

#1 UNL 1 O #0 ALA 554.A O 0.568 2.332

#1 UNL 1 C #0 ARG 555.A CA 0.565 3.075

#1 UNL 1 C #0 ARG 553.A C 0.564 3.016

#1 UNL 1 N #0 TYR 455.A CA 0.562 2.958

#1 UNL 1 N #0 ARG 624.A NH1 0.555 2.710

#1 UNL 1 C #0 ARG 553.A NH1 0.525 2.710

#1 UNL 1 C #0 ARG 555.A CA 0.523 2.967

#1 UNL 1 C #0 ARG 553.A CB 0.505 3.255

#1 UNL 1 C #0 ARG 553.A NE 0.503 2.732

#1 UNL 1 C #0 ARG 553.A C 0.502 2.958

#1 UNL 1 C #0 ARG 553.A NE 0.497 2.738

#1 UNL 1 C #0 ALA 554.A C 0.480 2.830

#1 UNL 1 N #0 ARG 624.A HE 0.471 2.169

#1 UNL 1 C #0 TYR 455.A HD2 0.463 2.147

#1 UNL 1 C #0 ARG 555.A H 0.461 2.149

#1 UNL 1 C #0 ARG 553.A CD 0.459 3.301

#1 UNL 1 C #0 ARG 553.A NH2 0.458 2.777

#1 UNL 1 C #0 ARG 555.A N 0.451 2.784

#1 UNL 1 C #0 ARG 624.A CZ 0.446 2.864

#1 UNL 1 O #0 ALA 554.A C 0.437 2.723

#1 UNL 1 C #0 ARG 624.A NH2 0.435 2.800

#1 UNL 1 C #0 ARG 553.A CZ 0.422 2.888

#1 UNL 1 C #0 ARG 553.A NE 0.415 3.090

#1 UNL 1 C #0 ARG 553.A 2HH2 0.414 2.466

#1 UNL 1 C #0 ARG 555.A C 0.412 2.898

#1 UNL 1 C #0 ARG 624.A NH2 0.412 2.823

#1 UNL 1 C #0 ARG 624.A 2HH2 0.410 2.470

#1 UNL 1 C #0 ARG 624.A CD 0.398 3.092

#1 UNL 1 O #0 ARG 553.A 2HH2 0.395 2.025

#1 UNL 1 C #0 ARG 553.A NE 0.393 2.842

#1 UNL 1 O #0 ARG 553.A NH1 0.391 2.694

#1 UNL 1 C #0 ARG 624.A NE 0.390 2.845

#1 UNL 1 O #0 ARG 553.A CZ 0.386 2.734

#1 UNL 1 O #0 ARG 553.A CZ 0.386 2.774

#1 UNL 1 C #0 ARG 555.A CA 0.378 3.112

#1 UNL 1 C #0 ASP 452.A OD1 0.378 2.982

#1 UNL 1 O #0 ARG 553.A NH1 0.373 2.712

#1 UNL 1 C #0 ARG 624.A HE 0.373 2.237

#1 UNL 1 C #0 ARG 553.A 1HH2 0.371 2.239

#1 UNL 1 C #0 ARG 553.A CZ 0.362 3.218

#1 UNL 1 O #0 ARG 553.A 1HH2 0.334 2.086

#1 UNL 1 C #0 ALA 554.A CA 0.330 3.160

#1 UNL 1 O #0 ARG 553.A 1HH1 0.327 2.133

#1 UNL 1 C #0 ALA 554.A CA 0.326 3.164

#1 UNL 1 C #0 ARG 553.A 2HH2 0.319 2.561

#1 UNL 1 C #0 ARG 553.A 2HH1 0.317 2.563

#1 UNL 1 C #0 ARG 555.A CB 0.317 3.173

#1 UNL 1 O #0 ALA 554.A O 0.313 2.627

#1 UNL 1 C #0 TYR 455.A CD2 0.296 3.014

#1 UNL 1 C #0 ARG 624.A 2HH2 0.288 2.322

#1 UNL 1 C #0 ASP 452.A OD1 0.285 2.805

#1 UNL 1 C #0 ALA 554.A C 0.280 3.030

#1 UNL 1 C #0 ARG 553.A NH2 0.274 3.231

#1 UNL 1 C #0 ARG 553.A CD 0.272 3.488

#1 UNL 1 C #0 ARG 624.A HE 0.265 2.345

#1 UNL 1 C #0 THR 556.A N 0.241 2.994

#1 UNL 1 C #0 ARG 624.A NE 0.240 2.995

#1 UNL 1 C #0 TYR 455.A CE1 0.237 3.073

#1 UNL 1 C #0 TYR 455.A CG 0.229 3.081

#1 UNL 1 C #0 ALA 554.A N 0.228 3.277

#1 UNL 1 C #0 ARG 553.A 2HH1 0.222 2.388

#1 UNL 1 C #0 ARG 553.A 1HH2 0.221 2.659

#1 UNL 1 C #0 ARG 553.A CD 0.217 3.543

#1 UNL 1 C #0 ARG 624.A CZ 0.215 3.095

#1 UNL 1 C #0 ARG 553.A O 0.214 3.026

#1 UNL 1 C #0 ARG 555.A CG 0.214 3.276

#1 UNL 1 C #0 ALA 554.A C 0.187 3.273

#1 UNL 1 C #0 ARG 624.A CD 0.180 3.580

#1 UNL 1 C #0 ARG 624.A CD 0.172 3.318

#1 UNL 1 C #0 ARG 553.A O 0.169 2.921

#1 UNL 1 C #0 LYS 621.A CA 0.169 3.591

#1 UNL 1 C #0 ARG 553.A HE 0.167 2.713

#1 UNL 1 N #0 ARG 624.A NH2 0.166 3.099

#1 UNL 1 C #0 ARG 555.A CG 0.156 3.334

#1 UNL 1 O #0 ARG 553.A CD 0.154 3.186

#1 UNL 1 O #0 ARG 555.A O 0.150 2.750

#1 UNL 1 C #0 ARG 555.A CG 0.147 3.343

#1 UNL 1 O #0 ARG 555.A CG 0.144 3.196

#1 UNL 1 N #0 ARG 553.A NH2   0.132 3.133

#1 UNL 1 O #0 ARG 553.A 2HH1 0.126 2.334

#1 UNL 1 O #0 TYR 455.A CD2   0.124 2.996

#1 UNL 1 C #0 ARG 553.A C   0.124 3.186

#1 UNL 1 C #0 ARG 553.A HE   0.114 2.496

#1 UNL 1 C #0 ARG 555.A CB   0.112 3.528

#1 UNL 1 C #0 ARG 553.A HE   0.108 2.772

#1 UNL 1 N #0 ARG 624.A CG   0.106 3.414

#1 UNL 1 O #0 THR 556.A CA   0.101 3.199

#1 UNL 1 C #0 ARG 555.A C   0.088 3.222

#1 UNL 1 C #0 ARG 624.A CD   0.087 3.403

#1 UNL 1 C #0 ARG 624.A NH2   0.087 3.418

#1 UNL 1 O #0 ARG 555.A CG   0.084 3.216

#1 UNL 1 C #0 ARG 555.A N   0.083 3.302

#1 UNL 1 C #0 ALA 554.A CA   0.075 3.565

#1 UNL 1 O #0 ARG 624.A CD   0.073 3.267

#1 UNL 1 C #0 TYR 455.A CZ   0.070 3.240

#1 UNL 1 C #0 ARG 553.A C   0.070 3.390

#1 UNL 1 C #0 ARG 553.A NH1   0.065 3.170

#1 UNL 1 C #0 ARG 553.A HE   0.065 2.545

#1 UNL 1 C #0 ARG 553.A 1HH2 0.048 2.562

#1 UNL 1 C #0 ALA 554.A O   0.047 3.313

#1 UNL 1 O #0 ARG 553.A NH1   0.045 3.040

#1 UNL 1 O #0 ASP 452.A CB   0.043 3.257

#1 UNL 1 C #0 ALA 554.A O   0.021 3.069

#1 UNL 1 C #0 ASP 452.A CG 0.011 3.299

#1 UNL 1 N #0 ARG 553.A CZ 0.008 3.332

#1 UNL 1 O #0 TYR 455.A CE2 0.004 3.116

#1 UNL 1 N #0 ARG 624.A 2HH1 0.002 2.638

#1 UNL 1 C #0 ALA 554.A O -0.012 3.372

#1 UNL 1 O #0 ARG 624.A CZ -0.024 3.144

#1 UNL 1 O #0 ARG 624.A 1HH2 -0.024 2.444

#1 UNL 1 C #0 ALA 554.A O -0.025 3.385

#1 UNL 1 O #0 ARG 553.A 1HH1 -0.036 2.496

#1 UNL 1 C #0 LYS 621.A CG -0.048 3.808

#1 UNL 1 O #0 TYR 455.A HE2 -0.052 2.472

#1 UNL 1 C #0 ARG 553.A CZ -0.055 3.635

#1 UNL 1 C #0 ARG 555.A N -0.059 3.444

#1 UNL 1 C #0 ARG 555.A CA -0.067 3.827

#1 UNL 1 C #0 ARG 624.A HE -0.071 2.951

#1 UNL 1 C #0 ALA 554.A C -0.073 3.653

#1 UNL 1 O #0 ASP 452.A OD2 -0.076 2.976

#1 UNL 1 O #0 ARG 553.A CZ -0.080 3.240

#1 UNL 1 C #0 ARG 553.A CG -0.084 3.844

#1 UNL 1 C #0 ARG 555.A CB -0.090 3.730

#1 UNL 1 O #0 THR 556.A CG2 -0.090 3.430

#1 UNL 1 O #0 ARG 553.A 2HH2 -0.096 2.516

#1 UNL 1 C #0 ALA 554.A CA -0.098 3.588

#1 UNL 1 C #0 ARG 624.A HE -0.104 2.984

#1 UNL 1 C #0 ARG 624.A NH1 -0.114 3.349

#1 UNL 1 C #0 SER 549.A OG   -0.115 3.375

#1 UNL 1 C #0 ARG 555.A O   -0.116 3.206

#1 UNL 1 C #0 THR 556.A H   -0.119 2.729

#1 UNL 1 C #0 ALA 554.A O   -0.119 3.209

#1 UNL 1 O #0 THR 556.A N   -0.125 3.210

#1 UNL 1 C #0 ARG 555.A N   -0.126 3.361

#1 UNL 1 O #0 ASP 452.A CA   -0.127 3.427

#1 UNL 1 C #0 ARG 553.A NE   -0.127 3.632

#1 UNL 1 C #0 ARG 553.A CG   -0.130 3.890

#1 UNL 1 C #0 ALA 554.A CA   -0.138 3.628

#1 UNL 1 C #0 TYR 455.A CD2   -0.138 3.448

#1 UNL 1 C #0 ARG 553.A C   -0.139 3.719

#1 UNL 1 O #0 ARG 624.A 2HH2 -0.144 2.604

#1 UNL 1 C #0 ARG 624.A 2HH2 -0.149 2.759

#1 UNL 1 C #0 ARG 555.A H   -0.152 2.912

#1 UNL 1 C #0 ARG 553.A CD   -0.154 3.914

#1 UNL 1 O #0 ARG 553.A NH2   -0.155 3.240

#1 UNL 1 N #0 TYR 455.A HD2   -0.159 2.799

#1 UNL 1 C #0 TYR 455.A CE2   -0.164 3.474

#1 UNL 1 N #0 TYR 455.A HD1   -0.164 2.804

#1 UNL 1 C #0 TYR 455.A CB   -0.168 3.658

#1 UNL 1 O #0 ARG 624.A CZ   -0.169 3.289

#1 UNL 1 C #0 ALA 554.A C   -0.169 3.629

#1 UNL 1 N #0 ARG 553.A 2HH2 -0.170 2.810

#1 UNL 1 C #0 ARG 553.A CA   -0.175 3.815

#1 UNL 1 O #0 ARG 624.A HE -0.179 2.639

#1 UNL 1 C #0 ALA 554.A C -0.187 3.767

#1 UNL 1 N #0 TYR 455.A C -0.188 3.528

#1 UNL 1 C #0 SER 549.A CB -0.195 3.835

#1 UNL 1 C #0 ASP 452.A CG -0.199 3.509

#1 UNL 1 O #0 ARG 624.A NE -0.210 3.295

#1 UNL 1 O #0 ARG 553.A NH2 -0.210 3.295

#1 UNL 1 C #0 ARG 553.A CB -0.212 3.852

#1 UNL 1 C #0 ARG 553.A 1HH1 -0.215 2.825

#1 UNL 1 C #0 TYR 455.A CD1 -0.217 3.527

#1 UNL 1 C #0 ARG 624.A CG -0.218 3.978

#1 UNL 1 C #0 ARG 553.A 1HH1 -0.219 2.829

#1 UNL 1 O #0 ARG 624.A NH2 -0.221 3.306

#1 UNL 1 C #0 TYR 455.A CA -0.221 3.711

#1 UNL 1 C #0 ARG 553.A O -0.229 3.589

#1 UNL 1 O #0 ARG 553.A 2HH1 -0.241 2.701

#1 UNL 1 C #0 ARG 553.A 1HH1 -0.243 3.123

#1 UNL 1 O #0 TYR 455.A HD2 -0.244 2.664

#1 UNL 1 O #0 ARG 553.A CZ -0.252 3.412

#1 UNL 1 C #0 ARG 553.A HE -0.256 2.866

#1 UNL 1 O #0 ARG 553.A 1HH2 -0.257 2.717

#1 UNL 1 O #0 ARG 624.A NH2 -0.261 3.306

#1 UNL 1 O #0 ALA 554.A CA -0.267 3.607

#1 UNL 1 C #0 TYR 455.A HD1 -0.268 2.878

#1 UNL 1 O #0 ARG 624.A HE -0.270 2.730

#1 UNL 1 C #0 ALA 554.A CA   -0.280 3.920

#1 UNL 1 C #0 ARG 553.A 1HH1 -0.281 3.161

#1 UNL 1 C #0 ALA 554.A CA   -0.281 3.921

#1 UNL 1 C #0 ALA 554.A N   -0.283 3.518

#1 UNL 1 C #0 ARG 553.A NE   -0.286 3.791

#1 UNL 1 C #0 ARG 624.A HE   -0.287 3.167

#1 UNL 1 O #0 ARG 624.A NE   -0.289 3.374

#1 UNL 1 O #0 ARG 553.A 2HH2 -0.293 2.753

#1 UNL 1 C #0 ARG 553.A CB   -0.301 3.791

#1 UNL 1 O #0 ARG 553.A CB   -0.307 3.647

#1 UNL 1 O #0 ARG 553.A CD   -0.307 3.647

#1 UNL 1 C #0 ARG 624.A NE   -0.308 3.813

#1 UNL 1 C #0 ARG 553.A NE   -0.310 3.545

#1 UNL 1 C #0 ARG 624.A NE   -0.310 3.815

#1 UNL 1 C #0 ARG 553.A 2HH1 -0.312 2.922

#1 UNL 1 C #0 LYS 621.A CB   -0.314 4.074

#1 UNL 1 N #0 ARG 553.A NH1   -0.317 3.582

#1 UNL 1 C #0 ARG 555.A CA   -0.319 4.079

#1 UNL 1 C #0 ARG 555.A H   -0.324 2.934

#1 UNL 1 C #0 ALA 554.A C   -0.332 3.642

#1 UNL 1 C #0 ARG 553.A CA   -0.334 4.094

#1 UNL 1 C #0 ARG 553.A NH2   -0.335 3.570

#1 UNL 1 O #0 ARG 553.A NE   -0.337 3.422

#1 UNL 1 C #0 ALA 554.A N   -0.341 3.726

#1 UNL 1 O #0 ARG 624.A NE   -0.345 3.390

#1 UNL 1 C #0 ARG 555.A CA -0.345 3.835

#1 UNL 1 O #0 TYR 455.A HD1 -0.349 2.769

#1 UNL 1 C #0 SER 549.A OG -0.351 3.611

#1 UNL 1 C #0 ALA 554.A C -0.357 3.817

#1 UNL 1 C #0 TYR 455.A CE2 -0.359 3.669

#1 UNL 1 C #0 ARG 555.A CB -0.360 3.850

#1 UNL 1 C #0 ARG 553.A NH1 -0.363 3.598

#1 UNL 1 C #0 ARG 553.A CG -0.364 3.854

#1 UNL 1 C #0 ASP 452.A CG -0.366 3.946

#1 UNL 1 C #0 ARG 555.A CA -0.367 4.007

#1 UNL 1 C #0 ALA 554.A C -0.367 3.947

#1 UNL 1 C #0 ARG 553.A NH1 -0.374 3.609

#1 UNL 1 C #0 ARG 555.A CA -0.375 4.015

#1 UNL 1 O #0 THR 556.A CB -0.375 3.715

#1 UNL 1 C #0 ASP 452.A OD1 -0.377 3.467

#1 UNL 1 C #0 ALA 554.A CA -0.377 4.137

#1 UNL 1 O #0 TYR 455.A CA -0.377 3.677

#1 UNL 1 C #0 SER 549.A CB -0.377 4.017

#1 UNL 1 O #0 ARG 553.A NH2 -0.389 3.434

#1 UNL 1 O #0 ARG 624.A 2HH2 -0.399 2.859

Pharmacophore 8:

332 contacts

atom1 atom2 overlap distance

#1 UNL 1 C #0 ARG 555.A CD 3.152 0.608

#1 UNL 1 C #0 ARG 555.A CZ 2.853 0.727

#1 UNL 1 C #0 ARG 555.A CZ 2.746 0.834

#1 UNL 1 C #0 ARG 555.A NE 2.475 1.030

#1 UNL 1 C #0 ARG 555.A NE 2.463 1.042

#1 UNL 1 C #0 ARG 555.A CD 2.434 1.326

#1 UNL 1 C #0 LYS 545.A HZ1 2.338 0.542

#1 UNL 1 C #0 ARG 555.A NH2 2.252 1.253

#1 UNL 1 C #0 LYS 545.A NZ 2.173 1.332

#1 UNL 1 C #0 LYS 545.A NZ 2.164 1.341

#1 UNL 1 C #0 ARG 555.A CD 2.110 1.650

#1 UNL 1 C #0 ARG 555.A NH1 2.095 1.410

#1 UNL 1 C #0 ARG 555.A CG 2.046 1.714

#1 UNL 1 C #0 ARG 555.A NH1 2.019 1.486

#1 UNL 1 C #0 ARG 555.A NE 1.971 1.534

#1 UNL 1 C #0 LYS 545.A CD 1.922 1.838

#1 UNL 1 C #0 ARG 555.A 2HH2 1.919 0.961

#1 UNL 1 C #0 ARG 555.A 2HH1 1.902 0.978

#1 UNL 1 C #0 ARG 555.A NH1 1.852 1.653

#1 UNL 1 C #0 LYS 545.A CD 1.851 1.909

#1 UNL 1 C #0 LYS 545.A HZ1 1.829 1.051

#1 UNL 1 C #0 ARG 555.A NH2 1.800 1.705

#1 UNL 1 C #0 LYS 545.A CE 1.800 1.960

#1 UNL 1 C #0 LYS 545.A HZ3 1.798 1.082

#1 UNL 1 C #0 ARG 555.A NH1 1.785 1.720

#1 UNL 1 C #0 ARG 555.A NH2 1.779 1.726

#1 UNL 1 C #0 LYS 545.A CE 1.761 1.999

#1 UNL 1 C #0 ARG 555.A CG 1.735 2.025

#1 UNL 1 C #0 ARG 555.A NE 1.721 1.784

#1 UNL 1 C #0 ARG 555.A CZ 1.710 1.870

#1 UNL 1 C #0 ARG 555.A CZ 1.676 1.904

#1 UNL 1 C #0 ARG 553.A CD 1.665 2.095

#1 UNL 1 C #0 LYS 545.A NZ 1.662 1.843

#1 UNL 1 C #0 ALA 547.A CB 1.633 2.127

#1 UNL 1 C #0 ARG 555.A CD 1.601 2.159

#1 UNL 1 C #0 ARG 555.A NH1 1.597 1.908

#1 UNL 1 C #0 LYS 545.A HZ3 1.575 1.305

#1 UNL 1 O #0 ARG 555.A N 1.574 1.471

#1 UNL 1 C #0 LYS 545.A HZ2 1.561 1.319

#1 UNL 1 C #0 ARG 555.A NE 1.560 1.945

#1 UNL 1 C #0 ARG 553.A CB 1.552 2.208

#1 UNL 1 C #0 ARG 555.A CZ 1.552 2.028

#1 UNL 1 C #0 LYS 545.A NZ 1.540 1.965

#1 UNL 1 C #0 ARG 555.A CZ 1.514 2.066

#1 UNL 1 C #0 LYS 545.A NZ 1.508 1.997

#1 UNL 1 C #0 ARG 555.A HE 1.491 1.389

#1 UNL 1 C #0 LYS 545.A HZ3 1.484 1.396

#1 UNL 1 C #0 ARG 555.A HE 1.483 1.277

#1 UNL 1 C #0 ARG 555.A NH2 1.483 2.022

#1 UNL 1 C #0 ARG 555.A HE 1.479 1.131

#1 UNL 1 C #0 ARG 555.A 2HH1 1.470 1.410

#1 UNL 1 C #0 LYS 545.A CE 1.459 2.301

#1 UNL 1 C #0 SER 549.A OG 1.455 1.925

#1 UNL 1 C #0 ARG 555.A CD 1.449 2.311

#1 UNL 1 C #0 ARG 553.A CB 1.412 2.348

#1 UNL 1 C #0 ALA 554.A C 1.393 2.187

#1 UNL 1 C #0 LYS 545.A CD 1.387 2.373

#1 UNL 1 C #0 ARG 555.A NE 1.386 1.999

#1 UNL 1 C #0 ARG 555.A CG 1.375 2.265

#1 UNL 1 C #0 ARG 553.A CG 1.358 2.402

#1 UNL 1 C #0 LYS 545.A NZ 1.344 2.161

#1 UNL 1 C #0 ARG 555.A N 1.318 2.187

#1 UNL 1 C #0 LYS 545.A CE 1.310 2.450

#1 UNL 1 C #0 SER 549.A OG 1.271 2.109

#1 UNL 1 C #0 ARG 555.A 2HH2 1.238 1.642

#1 UNL 1 C #0 ARG 555.A NH1 1.231 2.274

#1 UNL 1 C #0 LYS 545.A NZ 1.229 2.276

#1 UNL 1 C #0 LYS 545.A HZ2 1.212 1.668

#1 UNL 1 C #0 ARG 555.A HE 1.204 1.676

#1 UNL 1 O #0 ALA 554.A O 1.198 1.702

#1 UNL 1 C #0 ARG 555.A NH1 1.194 2.311

#1 UNL 1 C #0 LYS 545.A CG 1.176 2.584

#1 UNL 1 C #0 ARG 555.A 1HH2 1.165 1.715

#1 UNL 1 C #0 ARG 555.A HE 1.164 1.716

#1 UNL 1 C #0 ARG 555.A CD 1.161 2.479

#1 UNL 1 C #0 ARG 555.A CD 1.157 2.603

#1 UNL 1 O #0 ALA 554.A C 1.142 1.978

#1 UNL 1 C #0 ARG 555.A CA 1.124 2.636

#1 UNL 1 C #0 ARG 555.A NE 1.117 2.118

#1 UNL 1 C #0 LYS 545.A HZ3 1.116 1.764

#1 UNL 1 C #0 ARG 555.A 2HH1 1.113 1.767

#1 UNL 1 C #0 LYS 545.A HZ2 1.098 1.782

#1 UNL 1 C #0 ARG 555.A 1HH1 1.081 1.799

#1 UNL 1 C #0 ARG 555.A NH2 1.063 2.442

#1 UNL 1 C #0 SER 549.A OG 1.050 2.330

#1 UNL 1 C #0 LYS 545.A CE 1.047 2.713

#1 UNL 1 C #0 ARG 555.A 1HH1 1.045 1.835

#1 UNL 1 O #0 ALA 554.A C 1.031 2.089

#1 UNL 1 C #0 ARG 555.A HE 1.024 1.856

#1 UNL 1 C #0 ARG 555.A NE 1.002 2.503

#1 UNL 1 C #0 ARG 555.A NE 0.997 2.508

#1 UNL 1 C #0 LYS 545.A HZ2 0.994 1.886

#1 UNL 1 C #0 ARG 555.A CB 0.987 2.773

#1 UNL 1 O #0 ARG 555.A CA 0.962 2.338

#1 UNL 1 C #0 ARG 553.A CG 0.935 2.825

#1 UNL 1 C #0 ARG 555.A CZ 0.919 2.661

#1 UNL 1 C #0 LYS 545.A HZ3 0.919 1.961

#1 UNL 1 C #0 ARG 555.A CZ 0.917 2.663

#1 UNL 1 C #0 ARG 555.A CZ 0.901 2.679

#1 UNL 1 C #0 SER 549.A CB 0.887 2.873

#1 UNL 1 C #0 LYS 545.A NZ 0.881 2.624

#1 UNL 1 C #0 LYS 545.A HZ2 0.881 1.999

#1 UNL 1 C #0 ALA 554.A O 0.874 2.486

#1 UNL 1 C #0 LYS 545.A CE 0.867 2.893

#1 UNL 1 C #0 ARG 553.A CA 0.864 2.896

#1 UNL 1 O #0 LYS 545.A HZ2 0.857 1.603

#1 UNL 1 C #0 ARG 555.A CB 0.856 2.904

#1 UNL 1 C #0 ARG 555.A CD 0.853 2.907

#1 UNL 1 O #0 ARG 555.A H 0.850 1.570

#1 UNL 1 C #0 LYS 545.A HZ3 0.837 2.043

#1 UNL 1 C #0 ARG 555.A CZ 0.833 2.747

#1 UNL 1 C #0 ARG 555.A 2HH1 0.816 2.064

#1 UNL 1 C #0 ARG 555.A 2HH2 0.816 2.064

#1 UNL 1 C #0 LYS 545.A HZ1 0.812 2.068

#1 UNL 1 C #0 ARG 555.A CB 0.805 2.835

#1 UNL 1 C #0 ARG 555.A CB 0.803 2.957

#1 UNL 1 C #0 ILE 548.A O 0.802 2.558

#1 UNL 1 C #0 ALA 547.A CB 0.798 2.962

#1 UNL 1 C #0 ARG 555.A CG 0.769 2.991

#1 UNL 1 C #0 LYS 545.A HZ3 0.768 2.112

#1 UNL 1 C #0 ARG 555.A 1HH1 0.761 2.119

#1 UNL 1 C #0 ARG 553.A C 0.756 2.824

#1 UNL 1 O #0 ARG 555.A CB 0.742 2.558

#1 UNL 1 C #0 ARG 555.A CD 0.740 3.020

#1 UNL 1 C #0 ARG 555.A NH2 0.737 2.768

#1 UNL 1 C #0 ARG 555.A NE 0.732 2.773

#1 UNL 1 C #0 LYS 545.A HZ1 0.718 2.162

#1 UNL 1 C #0 ARG 555.A CG 0.718 3.042

#1 UNL 1 C #0 ARG 555.A 2HH2 0.713 2.167

#1 UNL 1 C #0 SER 549.A HG 0.708 2.172

#1 UNL 1 O #0 LYS 545.A NZ 0.704 2.381

#1 UNL 1 C #0 ARG 555.A 2HH1 0.703 2.177

#1 UNL 1 C #0 ALA 554.A CA 0.702 3.058

#1 UNL 1 C #0 ARG 555.A CD 0.701 3.059

#1 UNL 1 C #0 ARG 555.A 2HH1 0.699 2.181

#1 UNL 1 C #0 ARG 553.A 2HH1 0.697 2.183

#1 UNL 1 C #0 SER 549.A HG 0.690 2.190

#1 UNL 1 C #0 ARG 555.A CB 0.676 3.084

#1 UNL 1 O #0 ALA 554.A CA 0.675 2.625

#1 UNL 1 C #0 LYS 545.A NZ 0.671 2.834

#1 UNL 1 C #0 LYS 545.A HZ1 0.671 2.209

#1 UNL 1 C #0 ARG 555.A 2HH1 0.662 2.218

#1 UNL 1 C #0 SER 549.A OG 0.660 2.720

#1 UNL 1 C #0 ARG 555.A NE 0.636 2.869

#1 UNL 1 C #0 ARG 555.A 1HH2 0.612 2.268

#1 UNL 1 C #0 ARG 553.A CD 0.600 3.160

#1 UNL 1 C #0 SER 549.A OG 0.597 2.783

#1 UNL 1 C #0 ARG 555.A 1HH1 0.593 2.287

#1 UNL 1 C #0 ARG 555.A CD 0.589 3.171

#1 UNL 1 C #0 SER 549.A CB 0.580 3.180

#1 UNL 1 C #0 ARG 555.A CD 0.567 3.193

#1 UNL 1 C #0 LYS 545.A CD   0.561 3.199

#1 UNL 1 C #0 ARG 555.A 2HH1 0.557 2.323

#1 UNL 1 C #0 LYS 545.A HZ3   0.549 2.331

#1 UNL 1 O #0 ARG 555.A CA   0.547 2.753

#1 UNL 1 C #0 LYS 545.A CE   0.543 3.217

#1 UNL 1 O #0 ARG 553.A NH1   0.539 2.506

#1 UNL 1 C #0 ARG 553.A CD   0.532 3.228

#1 UNL 1 C #0 LYS 545.A CE   0.523 3.237

#1 UNL 1 C #0 ARG 553.A O   0.522 2.838

#1 UNL 1 C #0 ARG 555.A CD   0.517 3.243

#1 UNL 1 C #0 ARG 553.A C   0.509 3.071

#1 UNL 1 O #0 ARG 555.A N   0.506 2.539

#1 UNL 1 C #0 SER 549.A CB   0.500 3.260

#1 UNL 1 C #0 ARG 555.A NE   0.499 3.006

#1 UNL 1 C #0 ARG 553.A CD   0.493 3.267

#1 UNL 1 C #0 ARG 553.A O   0.488 2.872

#1 UNL 1 C #0 ARG 553.A O   0.467 2.893

#1 UNL 1 C #0 ARG 555.A NH1   0.464 3.041

#1 UNL 1 C #0 LYS 545.A CG   0.448 3.312

#1 UNL 1 C #0 LYS 545.A HZ3   0.443 2.437

#1 UNL 1 C #0 ARG 555.A CB   0.442 3.048

#1 UNL 1 C #0 ALA 547.A CB   0.439 3.321

#1 UNL 1 C #0 ARG 555.A CG   0.438 3.322

#1 UNL 1 O #0 ARG 553.A 2HH1 0.417 2.003

#1 UNL 1 C #0 ARG 555.A CG   0.410 3.080

#1 UNL 1 C #0 ARG 555.A 1HH2 0.402 2.478

#1 UNL 1 C #0 ARG 553.A NH1 0.396 3.109

#1 UNL 1 C #0 ARG 555.A CD 0.389 3.101

#1 UNL 1 C #0 ARG 555.A 1HH1 0.384 2.496

#1 UNL 1 C #0 ARG 555.A NE 0.380 3.125

#1 UNL 1 C #0 ARG 553.A NH1 0.377 3.128

#1 UNL 1 C #0 ARG 555.A NH2 0.374 3.131

#1 UNL 1 C #0 ARG 555.A HE 0.362 2.518

#1 UNL 1 C #0 ARG 555.A CB 0.357 3.403

#1 UNL 1 C #0 LYS 545.A CD 0.355 3.405

#1 UNL 1 C #0 ARG 555.A 2HH1 0.348 2.532

#1 UNL 1 C #0 ARG 555.A CZ 0.343 2.967

#1 UNL 1 C #0 ALA 554.A N 0.333 3.172

#1 UNL 1 C #0 ARG 555.A NH1 0.333 3.172

#1 UNL 1 C #0 ARG 555.A CZ 0.327 3.133

#1 UNL 1 C #0 ARG 555.A HE 0.315 2.565

#1 UNL 1 C #0 SER 549.A OG 0.311 3.069

#1 UNL 1 C #0 ARG 553.A O 0.310 3.050

#1 UNL 1 C #0 ARG 555.A CZ 0.305 3.275

#1 UNL 1 C #0 ALA 547.A CA 0.279 3.481

#1 UNL 1 O #0 ARG 553.A O 0.272 2.628

#1 UNL 1 C #0 ARG 553.A NE 0.272 3.233

#1 UNL 1 C #0 ILE 548.A C 0.259 3.321

#1 UNL 1 C #0 SER 549.A HG 0.246 2.634

#1 UNL 1 C #0 LYS 545.A CD 0.244 3.516

#1	UNL	1	C	#0	LYS	551.A	CD	0.238	3.522
#1	UNL	1	C	#0	LYS	545.A	HZ2	0.234	2.646
#1	UNL	1	C	#0	ARG	555.A	HE	0.230	2.650
#1	UNL	1	C	#0	LYS	545.A	CD	0.229	3.531
#1	UNL	1	C	#0	LYS	545.A	CB	0.219	3.541
#1	UNL	1	C	#0	ALA	547.A	CB	0.211	3.549
#1	UNL	1	C	#0	LYS	545.A	HZ2	0.205	2.675
#1	UNL	1	C	#0	ARG	555.A	NH1	0.204	3.301
#1	UNL	1	C	#0	ARG	553.A	2HH1	0.197	2.683
#1	UNL	1	C	#0	ARG	555.A	HE	0.193	2.687
#1	UNL	1	C	#0	ARG	555.A	NH2	0.188	3.047
#1	UNL	1	C	#0	ARG	555.A	H	0.182	2.698
#1	UNL	1	C	#0	ARG	555.A	CG	0.173	3.587
#1	UNL	1	C	#0	ARG	553.A	C	0.164	3.416
#1	UNL	1	C	#0	LYS	545.A	NZ	0.157	3.348
#1	UNL	1	C	#0	ARG	555.A	2HH2	0.155	2.455
#1	UNL	1	C	#0	ARG	555.A	CB	0.152	3.608
#1	UNL	1	C	#0	ARG	555.A	NH2	0.151	3.354
#1	UNL	1	C	#0	LYS	545.A	CG	0.147	3.613
#1	UNL	1	C	#0	SER	549.A	CB	0.144	3.616
#1	UNL	1	C	#0	ARG	555.A	1HH2	0.144	2.736
#1	UNL	1	C	#0	ARG	555.A	NH1	0.136	3.369
#1	UNL	1	C	#0	ARG	553.A	CA	0.131	3.629
#1	UNL	1	C	#0	ARG	555.A	CG	0.130	3.630
#1	UNL	1	C	#0	ALA	547.A	C	0.125	3.455

#1	UNL	1	C	#0	SER	549.A	HG	0.121	2.759
#1	UNL	1	C	#0	ARG	553.A	CB	0.102	3.658
#1	UNL	1	C	#0	LYS	545.A	HZ3	0.097	2.783
#1	UNL	1	C	#0	ARG	553.A	N	0.090	3.415
#1	UNL	1	O	#0	ALA	554.A	CA	0.083	3.217
#1	UNL	1	C	#0	ARG	555.A	CD	0.067	3.693
#1	UNL	1	O	#0	ALA	554.A	O	0.065	2.835
#1	UNL	1	C	#0	ARG	555.A	CB	0.065	3.695
#1	UNL	1	C	#0	SER	549.A	HG	0.064	2.816
#1	UNL	1	O	#0	LYS	545.A	HZ1	0.059	2.401
#1	UNL	1	C	#0	ARG	553.A	2HH1	0.059	2.821
#1	UNL	1	O	#0	LYS	545.A	HZ2	0.044	2.416
#1	UNL	1	C	#0	SER	549.A	CA	0.032	3.728
#1	UNL	1	C	#0	ARG	555.A	NE	0.023	3.482
#1	UNL	1	C	#0	ARG	555.A	CB	0.020	3.740
#1	UNL	1	C	#0	ARG	555.A	2HH1	0.017	2.863
#1	UNL	1	C	#0	LYS	545.A	CD	0.013	3.747
#1	UNL	1	C	#0	LYS	545.A	HZ3	0.003	2.877
#1	UNL	1	O	#0	ARG	553.A	C	-0.002	3.122
#1	UNL	1	C	#0	ARG	553.A	CB	-0.003	3.763
#1	UNL	1	C	#0	ARG	555.A	CA	-0.016	3.656
#1	UNL	1	C	#0	ARG	553.A	CZ	-0.028	3.608
#1	UNL	1	C	#0	ARG	555.A	CB	-0.029	3.789
#1	UNL	1	C	#0	ARG	553.A	CZ	-0.031	3.611
#1	UNL	1	C	#0	ARG	555.A	NE	-0.038	3.543

#1 UNL 1 C #0 ARG 555.A 1HH2 -0.038 2.918

#1 UNL 1 C #0 LYS 545.A HZ1 -0.046 2.926

#1 UNL 1 C #0 ALA 547.A CA -0.048 3.808

#1 UNL 1 O #0 ARG 553.A CG -0.054 3.354

#1 UNL 1 C #0 ARG 553.A CD -0.065 3.825

#1 UNL 1 C #0 ARG 555.A 1HH2 -0.070 2.950

#1 UNL 1 C #0 ARG 555.A CZ -0.075 3.655

#1 UNL 1 C #0 ARG 553.A 2HH1 -0.075 2.955

#1 UNL 1 O #0 LYS 545.A CE -0.081 3.421

#1 UNL 1 C #0 ILE 548.A N -0.081 3.586

#1 UNL 1 C #0 ARG 553.A 2HH1 -0.081 2.961

#1 UNL 1 C #0 LYS 545.A CD -0.084 3.844

#1 UNL 1 C #0 ARG 555.A NH2 -0.085 3.590

#1 UNL 1 C #0 ARG 553.A CG -0.089 3.849

#1 UNL 1 C #0 ALA 554.A C -0.102 3.682

#1 UNL 1 C #0 LYS 545.A HZ1 -0.104 2.984

#1 UNL 1 C #0 ALA 554.A N -0.110 3.615

#1 UNL 1 C #0 ARG 555.A N -0.111 3.616

#1 UNL 1 C #0 ARG 553.A C -0.117 3.697

#1 UNL 1 O #0 ALA 554.A N -0.124 3.169

#1 UNL 1 C #0 ARG 553.A CG -0.125 3.885

#1 UNL 1 C #0 ARG 555.A CA -0.126 3.886

#1 UNL 1 C #0 ARG 553.A NE -0.126 3.631

#1 UNL 1 C #0 ARG 555.A NE -0.131 3.636

#1 UNL 1 C #0 ARG 555.A CG -0.135 3.895

#1 UNL 1 C #0 ARG 553.A NH1 -0.135 3.640

#1 UNL 1 C #0 LYS 545.A HZ1 -0.139 3.019

#1 UNL 1 C #0 ARG 553.A NH1 -0.144 3.649

#1 UNL 1 C #0 ARG 553.A 2HH1 -0.151 3.031

#1 UNL 1 C #0 LYS 545.A CB -0.151 3.911

#1 UNL 1 C #0 ARG 555.A 2HH2 -0.152 3.032

#1 UNL 1 C #0 ARG 555.A CB -0.179 3.939

#1 UNL 1 C #0 ARG 555.A 2HH2 -0.186 3.066

#1 UNL 1 C #0 ARG 553.A CB -0.195 3.955

#1 UNL 1 C #0 ARG 555.A CG -0.199 3.959

#1 UNL 1 C #0 ARG 555.A NH1 -0.207 3.712

#1 UNL 1 C #0 ARG 555.A NH2 -0.212 3.597

#1 UNL 1 O #0 LYS 545.A NZ -0.213 3.298

#1 UNL 1 O #0 ARG 555.A CB -0.214 3.514

#1 UNL 1 O #0 LYS 545.A HZ1 -0.217 2.677

#1 UNL 1 O #0 ALA 554.A N -0.222 3.267

#1 UNL 1 C #0 LYS 545.A CE -0.222 3.982

#1 UNL 1 C #0 ALA 547.A CB -0.224 3.984

#1 UNL 1 C #0 ARG 553.A CG -0.225 3.985

#1 UNL 1 C #0 ALA 547.A CB -0.227 3.987

#1 UNL 1 C #0 LYS 545.A NZ -0.236 3.741

#1 UNL 1 C #0 ARG 555.A NH1 -0.242 3.747

#1 UNL 1 C #0 LYS 551.A CB -0.243 4.003

#1 UNL 1 C #0 ARG 555.A CA -0.244 4.004

#1 UNL 1 C #0 ALA 547.A N -0.244 3.749

#1 UNL 1 C #0 ARG 555.A 1HH1 -0.249 3.129

#1 UNL 1 O #0 ARG 553.A 1HH1 -0.255 2.675

#1 UNL 1 C #0 ARG 555.A HE   -0.261 3.141

#1 UNL 1 C #0 ARG 553.A H   -0.266 3.146

#1 UNL 1 C #0 SER 549.A CB   -0.273 4.033

#1 UNL 1 C #0 ARG 553.A NH1   -0.274 3.779

#1 UNL 1 C #0 ARG 555.A NH2   -0.277 3.782

#1 UNL 1 C #0 ALA 547.A CB   -0.280 4.040

#1 UNL 1 C #0 ARG 553.A 2HH1 -0.283 3.163

#1 UNL 1 C #0 LYS 545.A HZ2   -0.289 3.169

#1 UNL 1 C #0 ARG 553.A CA   -0.298 4.058

#1 UNL 1 C #0 ARG 553.A C   -0.307 3.887

#1 UNL 1 C #0 ARG 555.A NH1   -0.310 3.815

#1 UNL 1 C #0 ILE 548.A CA   -0.339 4.099

#1 UNL 1 C #0 ARG 553.A CB   -0.340 4.100

#1 UNL 1 C #0 ARG 555.A CG   -0.343 4.103

#1 UNL 1 C #0 ILE 548.A O   -0.351 3.711

#1 UNL 1 C #0 ARG 553.A CA   -0.354 4.114

#1 UNL 1 C #0 ARG 555.A CA   -0.355 4.115

#1 UNL 1 C #0 ARG 555.A CB   -0.355 4.115

#1 UNL 1 C #0 LYS 545.A CE   -0.359 4.119

#1 UNL 1 C #0 ARG 555.A 2HH1 -0.365 3.245

#1 UNL 1 C #0 ARG 555.A CD   -0.366 4.126

#1 UNL 1 C #0 ARG 555.A CG   -0.368 4.128

#1 UNL 1 O #0 LYS 545.A NZ   -0.371 3.456

#1 UNL 1 C #0 ARG 555.A 2HH1 -0.372 3.252

#1 UNL 1 C #0 LYS 545.A HZ1 -0.378 3.258

#1 UNL 1 O #0 LYS 545.A CD -0.382 3.722

#1 UNL 1 C #0 ARG 555.A CA -0.391 4.151

#1 UNL 1 O #0 ARG 553.A CZ -0.392 3.512

#1 UNL 1 O #0 ARG 553.A C -0.397 3.517

Pharmacophore 9:

182 contacts

atom1 atom2 overlap distance

#1 UNL 1 C #0 LYS 551.A NZ 2.714 0.791

#1 UNL 1 C #0 LYS 551.A HZ2 2.321 0.559

#1 UNL 1 C #0 PHE 793.A O 2.123 1.117

#1 UNL 1 C #0 LYS 551.A CE 2.084 1.676

#1 UNL 1 C #0 ASP 164.A OD1 1.944 1.296

#1 UNL 1 C #0 LYS 798.A HZ2 1.942 0.668

#1 UNL 1 C #0 LYS 798.A NZ 1.880 1.505

#1 UNL 1 C #0 LYS 798.A HZ3 1.878 0.882

#1 UNL 1 N #0 LYS 551.A NZ 1.861 1.404

#1 UNL 1 C #0 LYS 798.A HZ2 1.783 0.977

#1 UNL 1 C #0 LYS 798.A NZ 1.781 1.454

#1 UNL 1 C #0 LYS 798.A HZ3 1.752 0.858

#1 UNL 1 C #0 LYS 798.A HZ1 1.733 0.877

#1 UNL 1 C #0 ASP 164.A CG 1.712 1.748

#1	UNL	1	C	#0	LYS	798.A	NZ	1.700	1.685
#1	UNL	1	C	#0	LYS	798.A	NZ	1.687	1.548
#1	UNL	1	C	#0	LYS	798.A	CD	1.678	1.962
#1	UNL	1	C	#0	LYS	798.A	NZ	1.662	1.723
#1	UNL	1	C	#0	LYS	798.A	NZ	1.612	1.623
#1	UNL	1	C	#0	LYS	551.A	HZ3	1.587	1.293
#1	UNL	1	C	#0	PHE	793.A	O	1.550	1.690
#1	UNL	1	C	#0	LYS	551.A	CD	1.544	2.216
#1	UNL	1	C	#0	PHE	793.A	C	1.489	1.971
#1	UNL	1	C	#0	LYS	798.A	CE	1.438	2.202
#1	UNL	1	C	#0	LYS	798.A	CE	1.387	2.253
#1	UNL	1	C	#0	ASP	164.A	OD1	1.376	1.864
#1	UNL	1	C	#0	ASP	164.A	OD2	1.372	1.868
#1	UNL	1	N	#0	LYS	551.A	HZ3	1.320	1.320
#1	UNL	1	C	#0	PHE	793.A	CB	1.300	2.340
#1	UNL	1	N	#0	LYS	551.A	HZ2	1.291	1.349
#1	UNL	1	C	#0	LYS	798.A	HZ1	1.264	1.496
#1	UNL	1	C	#0	LYS	798.A	CD	1.263	2.377
#1	UNL	1	C	#0	LYS	798.A	HZ2	1.239	1.521
#1	UNL	1	C	#0	PHE	793.A	O	1.201	2.039
#1	UNL	1	C	#0	LYS	551.A	HZ1	1.179	1.701
#1	UNL	1	C	#0	LYS	798.A	HZ3	1.166	1.594
#1	UNL	1	C	#0	ASP	164.A	CG	1.067	2.393
#1	UNL	1	C	#0	LYS	798.A	CE	1.049	2.441
#1	UNL	1	C	#0	VAL	166.A	CB	1.045	2.595

#1 UNL 1 C #0 LYS 551.A HZ3 1.040 1.570

#1 UNL 1 N #0 LYS 551.A HZ1 1.031 1.609

#1 UNL 1 C #0 VAL 166.A CB 0.995 2.645

#1 UNL 1 C #0 LYS 798.A HZ3 0.958 1.652

#1 UNL 1 C #0 LYS 798.A CE 0.941 2.549

#1 UNL 1 C #0 LYS 798.A HZ1 0.931 1.679

#1 UNL 1 C #0 LYS 798.A CE 0.930 2.710

#1 UNL 1 C #0 LYS 551.A NZ 0.911 2.324

#1 UNL 1 C #0 ASP 164.A OD2 0.886 2.354

#1 UNL 1 C #0 GLU 167.A H 0.879 1.881

#1 UNL 1 N #0 LYS 798.A HZ1 0.857 1.783

#1 UNL 1 C #0 PHE 793.A CA 0.829 2.811

#1 UNL 1 C #0 GLU 167.A N 0.799 2.586

#1 UNL 1 C #0 PHE 793.A C 0.797 2.663

#1 UNL 1 C #0 PHE 793.A CA 0.784 2.856

#1 UNL 1 S #0 LYS 551.A HZ3 0.782 1.988

#1 UNL 1 C #0 LYS 551.A NZ 0.779 2.456

#1 UNL 1 C #0 PHE 793.A CB 0.758 2.882

#1 UNL 1 C #0 VAL 166.A CA 0.756 2.884

#1 UNL 1 C #0 LYS 798.A HZ2 0.748 1.862

#1 UNL 1 C #0 LYS 798.A CD 0.740 2.750

#1 UNL 1 C #0 LYS 798.A CE 0.711 2.779

#1 UNL 1 C #0 ASP 164.A OD2 0.704 2.386

#1 UNL 1 C #0 PHE 793.A CD2 0.665 2.795

#1 UNL 1 N #0 LYS 551.A CE 0.660 2.860

#1 UNL 1 C #0 VAL 166.A CG1 0.651 2.989

#1 UNL 1 C #0 PHE 793.A HD2 0.639 2.121

#1 UNL 1 C #0 LYS 798.A HZ2 0.618 2.142

#1 UNL 1 C #0 LYS 798.A HZ3 0.616 2.144

#1 UNL 1 C #0 PHE 793.A C   0.610 2.850

#1 UNL 1 N #0 LYS 798.A NZ   0.604 2.661

#1 UNL 1 C #0 VAL 166.A H   0.603 2.157

#1 UNL 1 C #0 ASP 164.A CG   0.572 2.738

#1 UNL 1 C #0 PHE 793.A HD2 0.558 2.202

#1 UNL 1 C #0 VAL 166.A N   0.550 2.835

#1 UNL 1 C #0 LYS 798.A HZ2 0.546 2.064

#1 UNL 1 C #0 LYS 551.A HZ2 0.529 2.081

#1 UNL 1 C #0 LYS 798.A HZ3 0.507 2.103

#1 UNL 1 C #0 PHE 793.A CD2 0.490 2.970

#1 UNL 1 C #0 ASP 164.A OD1 0.472 2.618

#1 UNL 1 C #0 LYS 798.A HZ3 0.439 2.171

#1 UNL 1 C #0 MET 794.A N   0.428 2.957

#1 UNL 1 C #0 LYS 798.A NZ   0.418 2.817

#1 UNL 1 C #0 ASP 164.A CB   0.413 3.227

#1 UNL 1 S #0 LYS 551.A NZ   0.410 2.985

#1 UNL 1 C #0 VAL 166.A H   0.405 2.355

#1 UNL 1 C #0 LYS 551.A HZ1 0.399 2.211

#1 UNL 1 C #0 VAL 166.A C   0.383 3.077

#1 UNL 1 C #0 PHE 793.A CG   0.377 3.083

#1 UNL 1 C #0 PHE 793.A O   0.373 2.717

#1	UNL	1	C	#0	LYS	798.A	HZ1	0.316	2.294
#1	UNL	1	O	#0	LYS	551.A	HZ2	0.309	2.111
#1	UNL	1	C	#0	LYS	798.A	HZ2	0.307	2.303
#1	UNL	1	O	#0	LYS	551.A	NZ	0.298	2.747
#1	UNL	1	C	#0	LYS	798.A	HZ1	0.286	2.474
#1	UNL	1	C	#0	PHE	793.A	CG	0.251	3.209
#1	UNL	1	C	#0	VAL	166.A	CA	0.229	3.411
#1	UNL	1	C	#0	MET	794.A	CA	0.224	3.416
#1	UNL	1	C	#0	PHE	793.A	N	0.224	3.161
#1	UNL	1	C	#0	LYS	798.A	NZ	0.222	3.013
#1	UNL	1	C	#0	ASP	164.A	CG	0.168	3.292
#1	UNL	1	C	#0	PRO	620.A	CG	0.164	3.326
#1	UNL	1	C	#0	LYS	798.A	CG	0.157	3.483
#1	UNL	1	C	#0	ASP	164.A	OD2	0.139	3.101
#1	UNL	1	C	#0	PHE	793.A	O	0.128	2.962
#1	UNL	1	C	#0	LYS	551.A	HZ1	0.123	2.487
#1	UNL	1	O	#0	PRO	620.A	CG	0.122	3.178
#1	UNL	1	C	#0	MET	794.A	CA	0.119	3.521
#1	UNL	1	C	#0	ASP	164.A	OD2	0.116	3.124
#1	UNL	1	C	#0	LYS	798.A	CD	0.105	3.385
#1	UNL	1	C	#0	PHE	793.A	H	0.095	2.665
#1	UNL	1	C	#0	GLU	167.A	N	0.092	3.293
#1	UNL	1	C	#0	LYS	551.A	CG	0.089	3.671
#1	UNL	1	C	#0	VAL	166.A	N	0.080	3.305
#1	UNL	1	C	#0	LYS	798.A	HZ1	0.061	2.699

#1 UNL 1 C #0 VAL 166.A CG1 0.056 3.584

#1 UNL 1 C #0 LYS 798.A CD 0.052 3.438

#1 UNL 1 O #0 LYS 798.A CD 0.046 3.254

#1 UNL 1 C #0 VAL 166.A CB 0.043 3.597

#1 UNL 1 C #0 SER 795.A N 0.039 3.346

#1 UNL 1 C #0 GLU 167.A H 0.033 2.727

#1 UNL 1 C #0 LYS 798.A CD 0.032 3.608

#1 UNL 1 C #0 LYS 798.A CE 0.028 3.462

#1 UNL 1 C #0 ASP 164.A OD1 0.016 3.224

#1 UNL 1 C #0 PRO 620.A CG -0.019 3.659

#1 UNL 1 C #0 LYS 551.A HZ2 -0.020 2.630

#1 UNL 1 C #0 VAL 166.A CB -0.029 3.669

#1 UNL 1 N #0 PRO 620.A CB -0.030 3.550

#1 UNL 1 C #0 VAL 166.A CG2 -0.030 3.670

#1 UNL 1 C #0 PHE 793.A CB -0.030 3.520

#1 UNL 1 S #0 LYS 551.A CE -0.035 3.685

#1 UNL 1 C #0 GLU 167.A CG -0.037 3.677

#1 UNL 1 C #0 VAL 166.A CG2 -0.044 3.684

#1 UNL 1 C #0 LYS 798.A CE -0.046 3.536

#1 UNL 1 C #0 SER 795.A H -0.065 2.825

#1 UNL 1 C #0 LYS 798.A HZ1 -0.068 2.678

#1 UNL 1 C #0 ASP 164.A OD2 -0.069 3.159

#1 UNL 1 C #0 VAL 166.A C -0.075 3.535

#1 UNL 1 N #0 LYS 551.A CD -0.079 3.599

#1 UNL 1 N #0 PRO 620.A CG -0.080 3.600

#1 UNL 1 O #0 SER 795.A H -0.083 2.503

#1 UNL 1 C #0 ASP 164.A OD1 -0.088 3.328

#1 UNL 1 C #0 LYS 551.A CE -0.099 3.589

#1 UNL 1 C #0 LYS 551.A HZ3 -0.102 2.712

#1 UNL 1 C #0 GLU 167.A CA -0.112 3.752

#1 UNL 1 C #0 ASP 164.A CG -0.118 3.578

#1 UNL 1 O #0 LYS 551.A HZ1 -0.122 2.542

#1 UNL 1 C #0 PHE 793.A N -0.131 3.516

#1 UNL 1 C #0 PHE 793.A O -0.136 3.226

#1 UNL 1 C #0 LYS 798.A CG -0.140 3.780

#1 UNL 1 C #0 LYS 798.A HZ1 -0.140 2.750

#1 UNL 1 C #0 GLU 167.A H -0.141 2.901

#1 UNL 1 C #0 GLU 167.A CB -0.172 3.812

#1 UNL 1 C #0 PHE 165.A H -0.178 2.938

#1 UNL 1 C #0 PRO 620.A CB -0.182 3.672

#1 UNL 1 C #0 SER 795.A N -0.183 3.568

#1 UNL 1 N #0 LYS 798.A HZ2 -0.197 2.837

#1 UNL 1 C #0 MET 794.A N -0.202 3.587

#1 UNL 1 C #0 ASP 164.A OD2 -0.215 3.455

#1 UNL 1 S #0 LYS 551.A CD -0.216 3.866

#1 UNL 1 C #0 ASP 164.A CA -0.218 3.858

#1 UNL 1 C #0 GLU 167.A CG -0.220 3.860

#1 UNL 1 C #0 ASP 164.A CB -0.223 3.863

#1 UNL 1 C #0 VAL 166.A CA -0.224 3.864

#1 UNL 1 O #0 SER 795.A OG -0.228 3.148

#1 UNL 1 N #0 LYS 798.A HZ3 -0.234 2.874

#1 UNL 1 C #0 MET 794.A C -0.256 3.716

#1 UNL 1 C #0 GLU 167.A N -0.258 3.643

#1 UNL 1 C #0 VAL 166.A CG1 -0.259 3.899

#1 UNL 1 N #0 LYS 551.A HZ3 -0.271 2.911

#1 UNL 1 N #0 LYS 551.A NZ -0.274 3.539

#1 UNL 1 N #0 LYS 798.A CE -0.274 3.794

#1 UNL 1 C #0 PHE 165.A N -0.282 3.667

#1 UNL 1 C #0 PHE 793.A CB -0.312 3.952

#1 UNL 1 C #0 PRO 620.A CG -0.315 3.805

#1 UNL 1 C #0 LYS 798.A NZ -0.332 3.567

#1 UNL 1 C #0 PRO 620.A CB -0.354 3.844

#1 UNL 1 C #0 LYS 551.A CE -0.358 3.848

#1 UNL 1 N #0 LYS 798.A NZ -0.359 3.624

#1 UNL 1 C #0 LYS 551.A NZ -0.368 3.603

#1 UNL 1 C #0 PHE 793.A CA -0.375 4.015

#1 UNL 1 C #0 MET 794.A C -0.380 3.840

#1 UNL 1 N #0 LYS 798.A HZ2 -0.398 3.038
