# Supplementary material for: Digging for the discovery of SARS-CoV-2 nsp12 inhibitors: a pharmacophore-based and molecular dynamics simulation study
Source: Future Virol. 2022 Aug 8:10.2217/fvl-2022-0054. doi: 10.2217/fvl-2022-0054 (PMC9370102; doi:10.2217/fvl-2022-0054)
Supplement: Supplementary file 3 [file supplementary-table-3.pdf]

Table S3. The affinity of identified hits to the respective pharmacophore

	Pharmacophore N0.1	
**ID**	**Identifier**	**Affinity (Kcal.mol^{-1})**
1	ZINC05515600	-4.7
	**Pharmacophore N0.2**	
**ID**	**Identifier**	**Affinity (Kcal.mol^{-1})**
1	ZINC65548703	-5.8
2	ZINC13482269	-7.2
3	ZINC49600254	-7.9
4	ZINC63303082	-8.5
5	ZINC38787536	-7.7
6	ZINC59370296	-7.1
7	ZINC59370300	-7.3
8	ZINC39997072	-8.1
9	ZINC65458835	-8.4
10	ZINC39992058	-8.4
11	ZINC39995255	-7.6
12	ZINC63527334	-7.5
13	ZINC09970427	-7.2
	**Pharmacophore N0.3**	
**ID**	**Identifier**	**Affinity (Kcal.mol^{-1})**
1	ZINC40436697	-7.2
2	ZINC93530025	-7.1
3	ZINC39644846	-4.6
4	ZINC04096710	-8.9
	**Pharmacophore N0.4**	
**ID**	**Identifier**	**Affinity (Kcal.mol^{-1})**
1	ZINC52981502	-7.7
2	ZINC03947430	-10.4
3	ZINC49771970	-8.7
4	ZINC22583252	-5.7
5	ZINC02038733	-8.4
6	ZINC15449299	-7.7
7	ZINC72209393	-8.2
8	ZINC03977803	-11
9	ZINC04098512	-9.8
10	ZINC35271732	-6.7
11	ZINC31170249	-8.2
12	ZINC13121743	-8.7
13	ZINC01747302	-7.7
14	ZINC00525469	-7.7
15	ZINC35465518	-7.6

16	ZINC03999911	-7.3
17	ZINC35271800	-7.2
18	ZINC14642422	-7.7
19	ZINC00236330	-8
20	ZINC24716660	-7.4
21	ZINC00490783	-7.6
22	ZINC03999863	-7.6
23	ZINC00490784	-8.9
24	ZINC00490785	-8.2
25	ZINC70691602	-8.3
26	ZINC72332860	-7.9
27	ZINC67912441	-7.8
28	ZINC67903431	-8.4
29	ZINC04801867	-9.5
30	ZINC39205164	-8.4
31	ZINC64450386	-8.3
32	ZINC03089883	-8.5
33	ZINC63251885	-8.2
34	ZINC64103317	-9
35	ZINC63637361	-9
36	ZINC64684089	-8
37	ZINC64684086	-7.6
38	ZINC63547534	-6.4
39	ZINC70691603	-7.4
40	ZINC04543715	-10.3
41	ZINC59676742	-8
42	ZINC85866695	-10.5
43	ZINC85867003	-8.3
44	ZINC85902197	-8.1
45	ZINC85893874	-8.2
46	ZINC76821021	-7.6
47	ZINC95486188	-10.4
48	ZINC85866838	-9.5
49	ZINC85866826	-9.4
50	UDC2_42651	-7.6
51	UDC2_26790	-7.9
52	UDC2_23249	-7.9
53	UDC2_9106	-6.7
54	UDC2_3792	-6.5
55	UDC2_19464	-7.8
56	UDC2_24084	-7.2
57	UDC2_21472	-7.8

Pharmacophore N0.5

ID	Identifier	Affinity (Kcal.mol^{-1})
1	ZINC12358611	-4.8
2	ZINC05638760	-8.8
3	ZINC16978810	-4.8
4	ZINC05356892	-5.2
5	ZINC05353810	-5.1
6	ZINC00492812	-5.2
7	ZINC17993173	-5.2
8	ZINC02510042	-5.2
9	ZINC04729628	-5.8
10	ZINC06733581	-6
11	ZINC38574060	-5.9
12	ZINC38574059	-5.2
13	ZINC04812411	-5.9
14	ZINC17224098	-5.7
15	ZINC55120853	-5.7
16	ZINC17248941	-6.1
17	ZINC17248954	-5.5
18	ZINC19143824	-6.9
19	ZINC16134045	-6.5
20	ZINC13687774	-6.5
21	ZINC13098322	-6.2
22	ZINC13098321	-6.3
23	ZINC04750868	-5.8
24	ZINC13764386	-7.5
25	ZINC75253788	-6
26	ZINC55525134	-7.1
27	ZINC38823808	-6
28	ZINC38823806	-6.9
29	ZINC05615705	-6.8
30	ZINC13572606	-6.9
31	ZINC05289674	-7
32	ZINC59452414	-6.8
33	ZINC00188127	-7.8
34	ZINC37624385	-6.5
35	ZINC18275888	-6.5
36	ZINC13153890	-6.6
37	ZINC13154579	-6.8
38	ZINC83252773	-7.3
39	ZINC05370644	-7.1
40	ZINC44709723	-7.6
41	ZINC59452412	-6.9
42	ZINC16958413	-6.6

43	ZINC04535563	-5.8
44	ZINC15919609	-8
45	ZINC40556177	-6.6
46	ZINC04596615	-7.4
47	ZINC16383477	-7.9
48	ZINC13098291	-7.5
49	ZINC13115801	-7.1
50	ZINC05910153	-8
51	ZINC19143828	-7.6
52	ZINC71772705	-7.5
53	ZINC33247064	-7.2
54	ZINC19143685	-8.5
55	ZINC05363800	-7.2
56	ZINC05532510	-7.8
57	ZINC13154582	-6.8
58	ZINC13154583	-7.7
59	ZINC05363789	-7.9
60	ZINC05123866	-8.1
61	ZINC07813295	-7.8
62	ZINC07813294	-7.6
63	ZINC07813280	-7.8
64	ZINC07813292	-7.6
65	ZINC07813293	-7.6
66	ZINC07813281	-8.1
67	ZINC07813284	-7.3
68	ZINC13147358	-7.6
69	ZINC07787400	-7.9
70	ZINC07813288	-8.3
71	ZINC07787375	-8.1
72	ZINC09958542	-7.8
73	ZINC05831991	-7.6
74	ZINC06780544	-9.7
75	ZINC16040271	-8.2
76	ZINC36371724	-7.9
77	ZINC13450622	-9.3
78	ZINC13450627	-10.1
79	ZINC09970285	-7.5
80	ZINC09970287	-7.4
81	ZINC09915709	-7.2
82	ZINC09915711	-7.1
83	ZINC09915710	-7.1

Pharmacophore N0.6

ID	Identifier	Affinity (Kcal.mol^{-1})

1	UDC1_45454	-7.5
2	UDC1_7513	-8
3	ZINC03875484	-9.3
4	ZINC11592735	-9.3
5	ZINC11592734	-8.6
6	ZINC03833821	-8.6
7	ZINC13540519	-7.8
8	ZINC03875560	-9.1
9	ZINC03831086	-9.7
10	ZINC03831088	-7.4
11	ZINC03831087	-8.4
12	ZINC03831089	-10
13	ZINC03876158	-10
14	ZINC04097304	-9.3
15	ZINC03882036	-9.1
16	ZINC03875332	-9.1
17	ZINC03876136	-8.6
18	ZINC03831370	-9.7
19	ZINC11592879	-9.6
20	ZINC03831369	-10.2
21	ZINC11592878	-9.1
22	ZINC03831367	-9.7
23	ZINC04097285	-9.1
24	ZINC03831096	-9.8
25	ZINC11592901	-8.9
26	ZINC11592903	-7.5
27	ZINC03830859	-9.5
28	ZINC11592904	-9.8
29	ZINC08552018	-7.7
30	ZINC11616267	-9.9
31	ZINC14879985	-9
32	ZINC11592791	-8.9
33	ZINC11592789	-8.9
34	ZINC04574572	-8.3
35	ZINC04574571	-9.3
36	ZINC11592790	-9.1
37	ZINC16052277	-9.6
38	ZINC08034234	-7.7
39	ZINC11592649	-8.3
40	ZINC04574440	-10
41	ZINC11592647	-8.8
42	ZINC11592648	-8.7
43	ZINC04340309	-9.4

44	ZINC03872834	-7.7
45	ZINC03872835	-7.4
46	ZINC04217537	-8.9
47	ZINC12493539	-9.4
48	ZINC03913922	-9
49	ZINC04096745	-8.6
50	ZINC04727169	-8
51	ZINC06858022	-9.1
52	ZINC40164245	-8.9
53	ZINC03977926	-9.2
54	ZINC03881945	-8.7
55	ZINC03977974	-7.8
56	ZINC04215431	-9.3
57	ZINC03977922	-9.5
58	ZINC03977923	-7.8
59	ZINC03983938	-9.6
60	ZINC03876099	-8.9
61	ZINC03977988	-8.8
62	ZINC03977991	-9.4
63	ZINC03977990	-7.2
64	ZINC03977989	-7.4
65	ZINC04213360	-8.1
66	ZINC04214095	-9
67	ZINC15449251	-7.8
68	ZINC35024607	-8.7
69	ZINC28713782	-7.9
70	ZINC15449372	-9.6
71	ZINC03978007	-8.1
72	ZINC15449374	-9.5
73	ZINC04097469	-7.8
74	ZINC19796022	-10.5
75	ZINC77257615	-7.2
76	ZINC39559999	-7.8
77	ZINC13325741	-9.4
78	ZINC38677144	-7.3
79	ZINC05645203	-8.2
80	ZINC31168077	-7.4
81	ZINC35458667	-7.1
82	ZINC05645205	-7.9
83	ZINC13326859	-8.6
84	ZINC13326865	-8.8
85	ZINC31169548	-7.2
86	ZINC03847420	-7

87	ZINC31169552	-6.3
88	ZINC15147978	-8.5
89	ZINC31160564	-7.6
90	ZINC22019057	-6.6
91	ZINC35271248	-8
92	ZINC35271245	-7.8
93	ZINC31165946	-8.2
94	ZINC35457977	-8.2
95	ZINC06718996	-8.5
96	ZINC31158247	-8.8
97	ZINC06718997	-8.6
98	ZINC05767450	-7.4
99	ZINC04084786	-8.7
100	ZINC35457984	-7.9
101	ZINC13326793	-8.8
102	ZINC05762294	-8.5
103	ZINC06718998	-8
104	ZINC13485659	-8.1
105	ZINC31162327	-8.1
106	ZINC31155859	-7.3
107	ZINC35454918	-7.8
108	ZINC35454921	-8.2
109	ZINC35454927	-8.4
110	ZINC31155843	-6
111	ZINC35454923	-7.8
112	ZINC77257433	-6
113	ZINC77257429	-6.1
114	ZINC77257431	-6.8
115	ZINC77257427	-7
116	ZINC15147992	-8.1
117	ZINC35270655	-8.1
118	ZINC35270658	-7.6
119	ZINC35270657	-7.8
120	ZINC03847031	-6.2
121	ZINC03847034	-6.7
122	ZINC35465379	-7.8
123	ZINC35465387	-8
124	ZINC35465385	-8
125	ZINC03983804	-7.1
126	ZINC13452213	-8.2
127	ZINC13337768	-8.7
128	ZINC14782914	-8.6
129	ZINC14782912	-7.9

130	ZINC13337766	-8.5
131	ZINC31164614	-8.3
132	ZINC49180937	-7.8
133	ZINC14781546	-7.5
134	ZINC14781550	-6.7
135	ZINC35271404	-7.5
136	ZINC06032154	-8.3
137	ZINC06067376	-8.3
138	ZINC14812957	-9
139	ZINC31161461	-9.3
140	ZINC14812959	-9.1
141	ZINC06032149	-8.5
142	ZINC06067369	-8.3
143	ZINC31161457	-8.9
144	ZINC31163421	-7.8
145	ZINC35458605	-8.1
146	ZINC35458611	-7.6
147	ZINC35458614	-6.5
148	ZINC35458609	-7.5
149	ZINC28971873	-8.7
150	ZINC04098201	-7.5
151	ZINC13330570	-7.3
152	ZINC13330568	-8.4
153	ZINC13330566	-8.6
154	ZINC13330564	-9.1
155	ZINC68603209	-7.2
156	ZINC68603205	-7.9
157	ZINC08681811	-8.1
158	ZINC31170249	-8.2
159	ZINC35455195	-8.7
160	ZINC35455184	-7.5
161	ZINC35271724	-8.5
162	ZINC31170255	-8.2
163	ZINC35455187	-8.8
164	ZINC31166537	-8
165	ZINC35464133	-6.7
166	ZINC35464136	-7.7
167	ZINC08552497	-9.5
168	ZINC08552496	-9.1
169	ZINC08552498	-9.5
170	ZINC31155041	-7.8
171	ZINC77257451	-7.8
172	ZINC14810057	-8.9

173	ZINC70665917	-9.3
174	ZINC70665918	-9.2
175	ZINC70665920	-9.2
176	ZINC13330186	-8.8
177	ZINC13330182	-8.5
178	ZINC31165260	-7.2
179	ZINC35270751	-7.2
180	ZINC31165264	-8.1
181	ZINC31165268	-7.5
182	ZINC31165256	-8.7
183	ZINC31154845	-9.9
184	ZINC16943064	-8.7
185	ZINC31163869	-8.3
186	ZINC28648780	-7.8
187	ZINC31158339	-7
188	ZINC04023072	-10.2
189	ZINC35466180	-6.3
190	ZINC35466182	-7.5
191	ZINC08295786	-7.5
192	ZINC05530113	-9.3
193	ZINC04081857	-9.1
194	ZINC04081856	-10.1
195	ZINC04081855	-9
196	ZINC04081854	-7.6
197	ZINC13540545	-8.9
198	ZINC77257596	-9
199	ZINC04081236	-9.9
200	ZINC04081240	-10
201	ZINC31158501	-8.9
202	ZINC85924058	-4.8
203	ZINC04334613	-5.9
204	ZINC86455752	-6
205	ZINC86872575	-5.6
206	ZINC87129603	-5.5
207	ZINC85901988	-5.9
208	ZINC86569766	-6.2
209	ZINC78932948	-6.3
210	ZINC89919259	-6.7
211	ZINC87140526	-5.5
212	ZINC87111361	-6.6
213	ZINC80576572	-6
214	ZINC72194767	-7.2
215	ZINC72194874	-7.2

216	ZINC85915430	-6.7
217	ZINC85679912	-5.9
218	ZINC85918430	-6.9
219	ZINC85899104	-7.3
220	ZINC85735189	-6
221	ZINC93340499	-6.2
222	ZINC87111358	-6.1
223	ZINC85899168	-5.7
224	ZINC87111354	-6.4
225	ZINC78381175	-6.6
226	ZINC85914550	-6.6
227	ZINC85900476	-6.4
228	ZINC85922762	-6.4
229	ZINC94616552	-7.5
230	ZINC93672401	-6.5
231	ZINC94529389	-6.4
232	ZINC37267152	-7.2
233	ZINC92753006	-7.3
234	ZINC95427200	-7.4
235	ZINC91910499	-7
236	ZINC72195173	-7.1
237	ZINC88725875	-6.5
238	ZINC34082874	-7.5
239	ZINC91406075	-6.6
240	ZINC85932291	-6.7
241	ZINC80576771	-6.3
242	ZINC80576547	-5.9
243	ZINC95423319	-7.5
244	ZINC80576708	-6.9
245	ZINC93672646	-7.3
246	ZINC93672105	-7.4
247	ZINC93672215	-7.5
248	ZINC91944974	-7.7
249	ZINC95028638	-7.3
250	ZINC91921557	-7.7
251	ZINC94617010	-7.4
252	ZINC74925960	-6.4
253	ZINC91921313	-7.6
254	ZINC03913372	-6.9
255	ZINC91732988	-6.8
256	ZINC93672515	-7.9
257	ZINC04334788	-8.2
258	ZINC91907160	-7.3

259	ZINC91905454	-7.3
260	ZINC93340394	-7.5
261	ZINC93671988	-6.6
262	ZINC78668975	-7.4
263	ZINC93672626	-7
264	ZINC93672439	-6.9
265	ZINC91912989	-7.8
266	ZINC92377530.	-7.1
267	ZINC91661367	-8
268	ZINC73440057	-7.1
269	ZINC91727233	-5.7
270	ZINC72195672	-6.3
271	ZINC74375714	-7.1
272	ZINC74140443	-6.4
273	ZINC73434984	-6.8
274	ZINC94617134	-8.1
275	ZINC78298525	-6.7
276	ZINC74106163	-7.4
277	ZINC74106158	-7.1
278	ZINC88728051	-7.6
279	ZINC93671824	-7.4
280	ZINC85253912	-6.1
281	ZINC85902475	-6.1
282	ZINC86186145	-6.6
283	ZINC75093648	-7
284	ZINC73503287	-7.3
285	ZINC91197177	-6.9
286	ZINC93340778	-6.1
287	ZINC92132860	-6.8
288	ZINC94618060	-7.7
289	ZINC94617176	-6.9
290	ZINC85329447	-8
291	ZINC73439056	-7.1
292	ZINC74524181	-6.8
293	ZINC91923579	-7.6
294	ZINC05304843	-7.4
295	ZINC93670213	-8.9
296	ZINC94617390	-7.6
297	ZINC74735372	-6.6
298	ZINC73630492	-6.8
299	ZINC77227489	-6.9
300	ZINC73777314	-8
301	ZINC94617066	-7.8

302	ZINC95427203	-8
303	ZINC79110038	-6.7
304	ZINC94293666	-6.5
305	ZINC85521482	-6.8
306	ZINC73814306	-6.9
307	ZINC12296959	-8.3
308	ZINC74823393	-7.1
309	ZINC92105307	-7.1
310	ZINC74432169	-7.4
311	ZINC12359339	-7.3
312	ZINC78381300	-6.2
313	ZINC73972514	-7.1
314	ZINC91611091	-7.5
315	ZINC74836654	-6.4
316	ZINC74836656	-6
317	ZINC89933375	-6.9
318	ZINC00485579	-7.1
319	ZINC93076971	-7.3
320	ZINC73914242	-7.8
321	ZINC84389085	-7.4
322	ZINC92396889	-7.1
323	ZINC74277870	-8.4
324	ZINC74320207	-7.3
325	ZINC80337266	-6.4
326	ZINC73941045	-7.9
327	ZINC93672579	-8
328	ZINC93672577	-8
329	ZINC74095664	-7.7
330	ZINC88409735	-7.5
331	ZINC93319372	-7.4
332	ZINC74165029	-8.9
333	ZINC90822961	-6.9
334	ZINC91906475	-7.3
335	ZINC73748233	-7.8
336	ZINC91923715	-7.9
337	ZINC73442900	-7
338	ZINC73820207	-6.7
339	ZINC79109988	-7.5
340	ZINC89321824	-8
341	ZINC91565072	-7.3
342	ZINC95442324	-8
343	ZINC77700941	-7.2
344	ZINC91348130	-8.1

345	ZINC79135379	-6.8
346	ZINC93341294	-7.4
347	ZINC20413721	-9.1
348	ZINC91963633	-8.1
349	ZINC74737892	-6.7
350	ZINC00134905	-8.6
351	ZINC84673384	-6.4
352	ZINC77678814	-7
353	ZINC84904702	-7.5
354	ZINC73569288	-8
355	ZINC73568568	-7.3
356	ZINC73535016	-7.5
357	ZINC77690877	-7.2
358	ZINC93204817	-7.7
359	ZINC81081958	-7.2
360	ZINC74846147	-7.3
361	ZINC80440273	-7.2
362	ZINC73866200	-7.9
363	ZINC74136648	-7.6
364	ZINC74886761	-7.5
365	ZINC74886760	-6.1
366	ZINC91348108	-7.7
367	ZINC74066109	-6.3
368	ZINC74770871	-6.7
369	ZINC84101429	-8.1
370	ZINC73540642	-7.9
371	ZINC81511025	-7.1
372	ZINC73779733	-8.1
373	ZINC92012904	-7.5
374	ZINC88735188	-7.4
375	ZINC91376904	-8.6
376	ZINC73630880	-7.2
377	ZINC74427333	-7
378	ZINC79001276	-7.6
379	ZINC73502809	-7.7
380	ZINC73833242	-7.3
381	ZINC77713320	-7.6
382	ZINC74129299	-7.1
383	ZINC73529072	-7.3
384	ZINC91258130	-7.6
385	ZINC91258133	-7.6
386	ZINC89764993	-7.6
387	ZINC75495029	-8.5

388	ZINC74921912	-7.7
389	ZINC73564357	-7.4
390	ZINC91902364	-7.3
391	ZINC89335567	-8.2
392	ZINC78254143	-9
393	ZINC74779743	-7.3
394	ZINC73755385	-7.6
395	ZINC94617063	-8.6
396	ZINC71769979	-9.6
397	ZINC73821771	-7.5
398	ZINC05211592	-8.7
399	ZINC21992801	-7.7
400	ZINC05519627	-9.2
401	ZINC89487157	-7.6
402	ZINC12522307	-7.9
403	ZINC73598152	-7
404	ZINC93341269	-6.8
405	ZINC74323796	-7.3
406	ZINC12824522	-8
407	ZINC91190239	-7.8
408	ZINC73633709	-8.2
409	ZINC80201447	-7.6
410	ZINC07787400	-7.8
411	ZINC73632353	-7.3
412	ZINC74175904	-8.1
413	ZINC91195090	-7.7
414	ZINC74132569	-7.7
415	ZINC73539803	-7.7
416	ZINC73809875	-7.6
417	ZINC74133167	-7.8
418	ZINC73626503	-7.1
419	ZINC73444072	-7.5
420	ZINC73749436	-7.7
421	ZINC73669116	-7.3
422	ZINC74060481	-7.6
423	ZINC93204814	-8.2
424	ZINC92358331	-8.1
425	ZINC80560607	-8.1
426	ZINC73723413	-7.6
427	ZINC73772680	-7.7
428	ZINC91968133	-10
429	ZINC74110166	-6.6
430	ZINC88787481	-8.3

431	ZINC73786183	-6.5
432	ZINC74140166	-7.8
433	ZINC34930583	-6.7
434	ZINC75495243	-7.6
435	ZINC84673396	-8.2
436	ZINC73873474	-8.4
437	ZINC73823688	-8
438	ZINC74314685	-7.3
439	ZINC73721389	-7.8
440	ZINC09302031	-8.8
441	ZINC33658206	-8.3
442	ZINC92399134	-7.8
443	ZINC73498140	-8.2
444	ZINC92849722	-7.6
445	ZINC30908515	-8.5
446	ZINC49785214	-9.8
447	ZINC03977967	-9.1
448	ZINC04082072	-9.6
449	ZINC38534680	-8.3
450	ZINC59381728	-10
451	ZINC64491003	-8.2
452	ZINC71769450	-9.8
453	ZINC15449351	-8.8
454	ZINC08789270	-8.7
455	ZINC38190801	-8.6
456	ZINC00713401	-7.3
457	ZINC35644965	-9.5
458	ZINC88466397	-8.5
459	ZINC35307838	-10.4
460	ZINC15218776	-9.3
461	ZINC08789618	-8.2
462	ZINC36371087	-8.2
463	ZINC43548000	-8.6
464	ZINC43553610	-8.5
465	ZINC03021786	-7.9
466	ZINC72326096	-9.2
467	ZINC43547792	-8.3
468	ZINC20906923	-9
469	ZINC20907369	-8.3
470	ZINC04222537	-9.4
471	ZINC04501355	-3.4
472	ZINC00730495	-3.7
473	ZINC00730493	-3.7

474	ZINC14589115	-4
475	ZINC05881295	-5.6
476	ZINC05298988	-4.8
477	ZINC05298936	-4.1
478	ZINC72194906	-6.6
479	ZINC13535829	-7.2
480	ZINC13555634	-7.4
481	ZINC08615759	-8.7
482	ZINC39342209	-7.5
483	ZINC05138848	-8.6
484	ZINC16213428	-8
485	ZINC44963161	-9.3
486	ZINC34272186	-10.1
487	ZINC34272188	-8.6
488	ZINC44963165	-7.7
489	ZINC44963163	-8
490	ZINC44963167	-7.7
491	ZINC34272187	-9.9
492	ZINC39886384	-7.2

Pharmacophore No.7

ID	Identifier	Affinity (Kcal.mol^{-1})
1	ZINC03830184	-7.6
2	ZINC11592789	-9.6
3	ZINC11592622	-10.5
4	ZINC03978484	-8.8
5	ZINC04019716	-8.3
6	ZINC04019704	-8
7	ZINC04149268	-8.1
8	ZINC77257427	-6.7
9	ZINC77257429	-7.1
10	ZINC31163421	-6.8
11	ZINC35271805	-6.9
12	ZINC13511167	-9.5
13	ZINC67912714	-9.2
14	ZINC31168190	-8.3
15	ZINC31168193	-8.5
16	ZINC08952338	-8.1
17	ZINC03978483	-8.2
18	ZINC67912718	-9.3
19	ZINC67912719	-8.6
20	ZINC11592657	-9.2
21	ZINC11592655	-8.5
22	ZINC35415700	-7.7

23	ZINC35415699	-7.4
24	ZINC31159205	-8.5
25	ZINC13827607	-9.9
26	ZINC12653547	-9.2
27	ZINC14615857	-10
28	ZINC38143725	-8.7
29	ZINC70691977	-8.7
30	ZINC70691980	-8.5
31	ZINC31163954	-9.1
32	ZINC31163958	-9
33	ZINC70691790	-9.4
34	ZINC12358680	-6.2
35	ZINC13154579	-6.7
36	ZINC09915821	-6
37	ZINC09970477	-6.8
38	ZINC67172014	-7.4
39	ZINC13098291	-7.8
40	ZINC13115801	-6.9
41	ZINC14454942	-8.9
42	ZINC94337019	-6.8
43	ZINC94337122	-6.2
44	ZINC35270887	-9.2
45	ZINC84317502	-7.2
46	ZINC94337063	-6.9
47	ZINC13154582	-7.6
48	ZINC09970377	-7
49	ZINC09970378	-7
50	ZINC07813295	-7.5
51	ZINC07813294	-7.7
52	ZINC00977258	-5.5
53	ZINC07813293	-7.7
54	ZINC72298340	-7.5
55	ZINC07813282	-8.1
56	ZINC84374510	-7.9
57	ZINC85468506	-8
58	ZINC65356761	-8.7
59	ZINC81253006	-7.9
60	ZINC16292190	-7.3
61	ZINC81801341	-7.1
62	ZINC81827195	-6.9
63	ZINC76687963	-9.1
64	ZINC13147358	-7.7
65	ZINC81252210	-8.3

66	ZINC81799234	-6.3
67	ZINC07787399	-7.7
68	ZINC71789727	-9.4
69	ZINC36056791	-8.8
70	ZINC63655485	-8.5
71	ZINC86528223	-7.9
72	ZINC81252142	-7.9
73	ZINC86527750	-8
74	ZINC07813289	-8.4
75	ZINC09915888	-7.6
76	ZINC58225254	-8.2
77	ZINC81823675	-7
78	ZINC67821680	-9.3
79	ZINC04529816	-6.1
80	ZINC05442860	-8.4
81	ZINC09915722	-7.1
82	ZINC09970240	-7.1
83	ZINC09970242	-6.4
84	ZINC09958546	-8.5
85	ZINC16927991	-7.8
86	ZINC06204415	-7.3
87	ZINC09852534	-7.5
88	ZINC09970228	-7
89	ZINC09970230	-7.5
90	ZINC10256809	-7.9
91	ZINC09852546	-7.6
92	ZINC09852548	-7.3
93	ZINC02281639	-7.5
94	ZINC09852597	-8.2
95	ZINC71789674	-7.8
96	ZINC05247536	-9
97	ZINC05247504	-8.9
98	ZINC10256819	-7.6
99	ZINC10275732	-8.2
100	ZINC38479784	-8
101	ZINC09852556	-7.2
102	ZINC09852557	-7.3
103	ZINC09852616	-7.5
104	ZINC83326415	-8.5
105	ZINC09915783	-6.7
106	ZINC05247505	-8.2
107	ZINC05247537	-8.2
108	ZINC05247506	-8.4

109	ZINC05247538	-8.9
110	ZINC39589378	-8.3
111	ZINC64657138	-7.4
112	ZINC09852647	-7.7
113	ZINC09852552	-7.6
114	ZINC10275754	-7.6
115	ZINC04974277	-9.2
116	ZINC04533484	-8
117	ZINC09852571	-7.1
118	ZINC09852569.	-7.3
119	ZINC95100271	-7.3
120	ZINC95486254	-8.1
121	ZINC39916010	-8
122	ZINC35024487	-7.9
123	ZINC36372249	-9.3
124	ZINC36372250	-8.2
125	ZINC09915714	-7.5
126	ZINC09915712	-7.6
127	ZINC09970279	-7.6
128	ZINC09852622	-8
129	ZINC09915755	-9.2
130	ZINC43735718	-9.2
131	ZINC44431354	-7.7
132	ZINC86860273	-8.9

Pharmacophore No.8

ID	Identifier	Affinity (Kcal.mol^{-1})
1	ZINC72320308	-7.3
2	ZINC67902735	-8.1
3	ZINC38143754	-9.1
4	ZINC39560159	-9.7
5	ZINC67903270	-8.7
6	ZINC67903269	-9.1
7	ZINC35570685	-10.2
8	ZINC38944039	-9.9
9	ZINC43510461	-10.1
10	ZINC67903241	-9.1

Pharmacophore No.9

ID	Identifier	Affinity (Kcal.mol^{-1})
1	UDC1_38998	-8.1
2	UDC2_19291	-6.8
3	UDC1_31501	-7.8
4	UDC1_32040	-7.7
5	UDC2_6926	-7.7

6	UDC1_28368	-8.2
7	UDC1_24158	-9.2
8	UDC1_8343	-7.3
9	ZINC03831616	-8.5
10	ZINC03831615	-8.3
11	ZINC03831617	-9.4
12	ZINC00001454	-6.2
13	ZINC03881412	-8.2
14	ZINC03831614	-9.7
15	ZINC33838895	-8.1
16	ZINC00287295	-7.1
17	ZINC31165410	-7.1
18	ZINC14811592	-8.6
19	ZINC20112767	-7
20	ZINC03936605	-8.3
21	ZINC15274349	-8
22	ZINC15274352	-8.6
23	ZINC15274351	-8.4
24	ZINC15274350	-8
25	ZINC03898789	-8.6
26	ZINC06030541	-8.3
27	ZINC06037280	-8.8
28	ZINC00526567	-7.4
29	ZINC12442417	-7.7
30	ZINC04026238	-7.6
31	ZINC14415571	-8.8
32	ZINC35458488	-8.6
33	ZINC14415575	-8.3
34	ZINC13309325	-8.3
35	ZINC35458493	-7.7
36	ZINC14415573	-8.1
37	ZINC13309321	-8.4
38	ZINC14415576	-8
39	ZINC13309323	-8.4
40	ZINC13309327	-8.3
41	ZINC04029766	-6.9
42	ZINC02575235	-5.9
43	ZINC00080786	-6
44	ZINC00338209	-5.5
45	ZINC00391406	-6.1
46	ZINC00394662	-6.1
47	ZINC00160491	-6.3
48	ZINC01703962	-6.4

49	ZINC00239015	-6.6
50	ZINC00156963	-6.7
51	ZINC34585829	-6.5
52	ZINC14775716	-6.2
53	ZINC02029134	-5.9
54	ZINC00088053	-6.5
55	ZINC01583371	-6.4
56	ZINC02243840	-7.5
57	ZINC15115361	-6.7
58	ZINC00900229	-7
59	ZINC00900231	-6.9
60	ZINC13484870	-6.5
61	ZINC14817882	-6.6
62	ZINC03200828	-5.8
63	ZINC03852584	-6.5
64	ZINC15204911	-6
65	ZINC15117999	-6.9
66	ZINC13521031	-6.8
67	ZINC72319967	-6.9
68	ZINC72319966	-6.9
69	ZINC15205786	-6.7
70	ZINC32634059	-8.1
71	ZINC34319583	-7.2
72	ZINC34319582	-8.3
73	ZINC28875643	-8.3
74	ZINC31158459	-9.2
75	ZINC14647533	-8.2
76	ZINC31162933	-9.6
77	ZINC31162937	-8.5
78	ZINC04655389	-7.8
79	ZINC05258651	-7.9
80	ZINC04025170	-8.6
81	ZINC04655390	-7.9
82	ZINC04655391	-7.3
83	ZINC03957079	-8.4
84	ZINC05258647	-7.5
85	ZINC04025169	-7.9
86	ZINC04098719	-9.9
87	ZINC49601467	-9.8
88	ZINC06066602	-8
89	ZINC18116328	-8.8
90	ZINC04084754	-8.4
91	ZINC04081891	-8.4

92	ZINC14647535	-8.4
93	ZINC02145068	-7.8
94	ZINC08792550	-8.3
95	ZINC08764316	-9
96	ZINC04039248	-7.3
97	ZINC13515668	-8.8
98	ZINC31165006	-8.1
99	ZINC13515667	-7.9
100	ZINC13521629	-7.8
101	ZINC03850734	-10.1
102	ZINC03850723	-8
103	ZINC35457955	-7.4
104	ZINC35457940	-7.7
105	ZINC35457948	-7.9
106	ZINC35457961	-7.8
107	ZINC35457951	-7.9
108	ZINC31162689	-7.6
109	ZINC35457944	-9.8
110	ZINC35457959	-8
111	ZINC35457471	-7.8
112	ZINC31161800	-7.9
113	ZINC35457481	-9.1
114	ZINC31161808	-7.5
115	ZINC35457478	-7.6
116	ZINC31161804	-7.9
117	ZINC31167242	-7.8
118	ZINC31167238	-7.8
119	ZINC31167246	-7.8
120	ZINC31167250	-7.8
121	ZINC14723274	-8.7
122	ZINC32124410	-6.6
123	ZINC32124021	-6.7
124	ZINC35457926	-7.8
125	ZINC35457933	-7.8
126	ZINC04029850	-7.5
127	ZINC31165002	-9.1
128	ZINC04083545	-8.3
129	ZINC33832362	-9.4
130	ZINC01563289	-6.4
131	ZINC36678684	-5.7
132	ZINC86798339	-6.1
133	ZINC39055607	-7.1
134	ZINC15783401	-6.9

135	ZINC87125268	-6.1
136	ZINC94785365	-7.8
137	ZINC29756600	-7.2
138	ZINC85144091	-6.4
139	ZINC23058143	-7.4
140	ZINC39342491	-4.5
141	ZINC93689193	-6.8
142	ZINC09096609	-6.8
143	ZINC94614606	-7.5
144	ZINC92178570	-8.2
145	ZINC94785367	-7.8
146	ZINC91692171	-6.8
147	ZINC63666199	-6.1
148	ZINC93688689	-8
149	ZINC05950708	-5.9
150	ZINC00566766	-8.3
151	ZINC16639938	-7.9
152	ZINC41845956	-7.8
153	ZINC94022318	-6
154	ZINC94785758	-6.7
155	ZINC93898651	-7.2
156	ZINC92541533	-7.3
157	ZINC06645850	-7.8
158	ZINC94812547	-7
159	ZINC41513519	-7.4
160	ZINC29752842	-8
161	ZINC37432769	-7.5
162	ZINC77612354	-6.8
163	ZINC17321654	-7.3
164	ZINC92471265	-7.4
165	ZINC39867673	-8.1
166	ZINC67894260	-8
167	ZINC07141248	-7
168	ZINC11666903	-8.2
169	ZINC93332767	-6.4
170	ZINC26728085	-6.8
171	ZINC39574670	-9.2
172	ZINC93287525	-7.4
173	ZINC40040217	-7.2
174	ZINC72125406	-7.6
175	ZINC92262376	-6.4
176	ZINC05370229	-7.7
177	ZINC92126496	-6.9

178	ZINC77613999	-7.4
179	ZINC89841690	-6.7
180	ZINC89832977	-7.4
181	ZINC89834380	-7.4
182	ZINC89771353	-7.3
183	ZINC17122858	-8.9
184	ZINC90199751	-7.1
185	ZINC38701582	-7.9
186	ZINC93480651	-8.3
187	ZINC63531600	-7.3
188	ZINC41512668	-8.3
189	ZINC39581569	-8.5
190	ZINC75155872	-7.4
191	ZINC08908735	-7.4
192	ZINC17055919	-8
193	ZINC29793559	-7.7
194	ZINC91861716	-6.2
195	ZINC04479052	-7.8
196	ZINC74020640	-8
197	ZINC39580000	-8.3
198	ZINC00094318	-8.9
199	ZINC08937686	-7.9
200	ZINC80792919	-8.5
201	ZINC10444997	-8
202	ZINC80879714	-7.9
203	ZINC38549409	-7.7
204	ZINC06819984	-7.7
205	ZINC20636460	-7.3
206	ZINC91754182	-8.6
207	ZINC39440473	-8.1
208	ZINC32309612	-8
209	ZINC72404927	-9.3
210	ZINC29964245	-8.1
211	ZINC93589651	-8.4
212	ZINC03903498	-6.9
213	ZINC22467501	-9.1
214	ZINC92278716	-7
215	ZINC89828879	-7.1
216	ZINC63493721	-7.6
217	ZINC90125625	-7.9
218	ZINC07260777	-7.6
219	ZINC93885337	-8.3
220	ZINC81275045	-7.3

221	ZINC66400907	-8.3
222	ZINC31818059	-8.1
223	ZINC93737936	-7.7
224	ZINC93482243	-7.3
225	ZINC92406975	-7.2
226	ZINC89781537	-6.8
227	ZINC93866708	-8.2
228	ZINC12770509	-8.3
229	ZINC89827034	-6.9
230	ZINC68156491	-7.9
231	ZINC89820807	-8.5
232	ZINC19358084	-8.9
233	ZINC61431517	-8.6
234	ZINC79586613	-7.7
235	ZINC16640651	-8.7
236	ZINC23750873	-8.1
237	ZINC48380479	-8.2
238	ZINC82982788	-8.1
239	ZINC80016413	-7.3
240	ZINC90406893	-7.3
241	ZINC89807003	-7.6
242	ZINC33254942	-8.4
243	ZINC67331297	-9
244	ZINC87410547	-9.3
245	ZINC89602055	-7.1
246	ZINC89752830	-7
247	ZINC20604327	-8.2
248	ZINC79482963	-8.1
249	ZINC74126230	-9.2
250	ZINC32473682	-8.3
251	ZINC93808544	-7.9
252	ZINC30692130	-9.7
253	ZINC32847976	-8.3
254	ZINC63856943	-9.2
255	ZINC93321714	-7.5
256	ZINC52951631	-9.9
257	ZINC79582350	-8.6
258	ZINC27637448	-8.3
259	ZINC89574468	-8.3
260	ZINC76881151	-9.1
261	ZINC64991953	-8.3
262	ZINC15629437	-9.6
263	ZINC15806151	-7.8

264	ZINC65420226	-7.3
265	ZINC38759880	-7.8
266	ZINC65297457	-8.3
267	ZINC72446111	-8.9
268	ZINC89860949	-8.5
269	ZINC65190585	-8.9
270	ZINC79749422	-7.5
271	ZINC01194052	-8.2
272	ZINC72440811	-8.2
273	ZINC89582412	-7.6
274	ZINC89769988	-8
275	ZINC83025725	-7.8
276	ZINC91683847	-7.8
277	ZINC39867882	-9.5
278	ZINC54120801	-10.3
279	ZINC71769864	-8.3
280	ZINC12770211	-8.3
281	ZINC89829402	-7.5
282	ZINC92312682	-8.3
283	ZINC77491524	-7.4
284	ZINC09834534	-9.7
285	ZINC92144520	-8
286	ZINC02444184	-7.9
287	ZINC12777827	-9.8
288	ZINC19898238	-7.7
289	ZINC12211619	-9.1
290	ZINC88461500	-7.7
291	ZINC92454089	-7.2
292	ZINC26047212	-8.4
293	ZINC58282638	-7.3
294	ZINC85438650	-8.3
295	ZINC67541423	-7.8
296	ZINC93868557	-7.2
297	ZINC71762471	-7.2
298	ZINC22355482	-8.7
299	ZINC89825835	-8.5
300	ZINC20115249	-7.6
301	ZINC73685080	-8.3
302	ZINC12776304	-7.3
303	ZINC72407300	-8.7
304	ZINC80764005	-8.7
305	ZINC67804677	-8.2
306	ZINC77219675	-7.9

307	ZINC79255573	-9
308	ZINC89768920	-7.4
309	ZINC89706048	-7.9
310	ZINC04901012	-8.3
311	ZINC16607218	-7.6
312	ZINC05898342	-7.2
313	ZINC17908682	-8.6
314	ZINC92810292	-8.4
315	ZINC91959983	-7.5
316	ZINC14712256	-8.2
317	ZINC93908137	-8.9
318	ZINC89593368	-7.6
319	ZINC18015724	-7.4
320	ZINC90798129	-8.4
321	ZINC93927443	-8.1
322	ZINC08718166	-7.5
323	ZINC00815888	-8.7
324	ZINC11486459	-8.5
325	ZINC36724841	-7.4
326	ZINC92104805	-8.4
327	ZINC40568958	-8.5
328	ZINC12776093	-9.3
329	ZINC95374990	-8.3
330	ZINC15062496	-8.8
331	ZINC63699169	-9.3
332	ZINC93987922	-9
333	ZINC08429007	-8.6
334	ZINC32899754	-6.8
335	ZINC19147102	-8
336	ZINC06667843	-8
337	ZINC48112481	-9.1
338	ZINC72003692	-7
339	ZINC15010688	-8.3
340	ZINC12852883	-8.4
341	ZINC92129298	-7.6
342	ZINC58326517	-7.7
343	ZINC12974523	-6.7
344	ZINC92034998	-9.3
345	ZINC10447167	-8.1
346	ZINC08007361	-8
347	ZINC92735992	-8.6
348	ZINC40785575	-8
349	ZINC23228310	-7.7

350	ZINC12039762	-8.4
351	ZINC11456442	-7.9
352	ZINC06180550	-7.7
353	ZINC21723626	-8.8
354	ZINC12889785	-9.3
355	ZINC06757463	-9.3
356	ZINC24587693	-8
357	ZINC32238917	-8.3
358	ZINC41005154	-7.9
359	ZINC92143031	-7.7
360	ZINC04142332	-6.2
361	ZINC93683993	-7.3
362	ZINC93868076	-8.3
363	ZINC33327063	-8.4
364	ZINC39867918	-9.1
365	ZINC12980938	-8.5
366	ZINC13482282	-9.9
367	ZINC15765566	-9.2
368	ZINC58443734	-8.2
369	ZINC12385053	-8.4
370	ZINC31324538	-8.9
371	ZINC12385071	-7.8
372	ZINC04717922	-8.5
373	ZINC12784726	-7.7
374	ZINC64953784	-8
375	ZINC40568968	-9.4
376	ZINC72010462	-9
377	ZINC12768618	-9.1
378	ZINC11910019	-8.3
379	ZINC72180724	-8.4
380	ZINC72440824	-8.5
381	ZINC32078187	-8.6
382	ZINC12385045	-8
383	ZINC72018209	-7.4
384	ZINC07560801	-9.8
385	ZINC71989883	-8.3
386	ZINC15819461	-8.8
387	ZINC01428687	-7.4
388	ZINC49835816	-7.9
389	ZINC20327208	-8.1
390	ZINC20327223	-8.5
391	ZINC64020246	-7.7
392	ZINC85665007	-7.7

393	ZINC41015782	-8.4
394	ZINC12229066	-8.1
395	ZINC12411303	-7.8
396	ZINC32933709	-8.6
397	ZINC64954298	-7.9
398	ZINC06090185	-7.3
399	ZINC01440053	-7.7
400	ZINC32771109	-8.8
401	ZINC33248356	-9.5
402	ZINC01582000	-7.5
403	ZINC14991264	-9
404	ZINC64998220	-8.4
405	ZINC62427689	-8.1
406	ZINC14183713	-7.9
407	ZINC32845976	-8.7
408	ZINC10995316	-9
409	ZINC33194533	-9.7
410	ZINC64954483	-8.3
411	ZINC40569045	-9.1
412	ZINC14943494	-7.6
413	ZINC09762400	-8.1
414	ZINC71828457	-9.2
415	ZINC33291889	-7.7
416	ZINC71892025	-8.5
417	ZINC20609419	-7.6
418	ZINC12385038	-7.4
419	ZINC12385036	-8.1
420	ZINC20406824	-7.4
421	ZINC64991930	-8.6
422	ZINC40569044	-8.1
423	ZINC15206016	-8.3
424	ZINC64954089	-8.9
425	ZINC64998215	-8.4
426	ZINC05575600	-5.6
427	ZINC03327259	-6
428	ZINC06375665	-5.8
429	ZINC06557181	-6.4
430	ZINC17122971	-8.2
431	ZINC05906246	-7.4
432	ZINC17128946	-7.3
433	ZINC17301324	-8
434	ZINC17172661	-6.9
435	ZINC17128949	-8.1

436	ZINC17321655	-7.7
437	ZINC17305055	-7.8
438	ZINC17356115	-8.7
439	ZINC16504195	-8.8
440	ZINC55544653	-7.4
441	ZINC11594992	-8.8
442	ZINC17910422	-9
443	ZINC11373127	-8.9
444	ZINC10492481	-8.3
445	ZINC17323644	-8.9
446	ZINC12039878	-7.9
447	ZINC18153615	-9.4
448	ZINC18153613	-8.5
449	ZINC12039882	-7.3
450	ZINC17908351	-8.3
451	ZINC17908681	-9.3
452	ZINC16639410	-8.3
453	ZINC10490356	-8.3
454	ZINC17296242	-9.5
455	ZINC27546564	-8.8
456	ZINC10373076	-9.3
457	ZINC39156736	-8.6
458	ZINC39156738	-8.8
459	ZINC10423642	-8.1
460	ZINC10423643	-8.1
461	ZINC09920276	-8.9
462	ZINC09312872	-8.1
463	ZINC09312871	-10
464	ZINC09013487	-7.6
465	ZINC16213428	-8
466	ZINC09332150	-7.5
467	ZINC08779077	-7.3
468	ZINC12815873	-8.4
469	ZINC18151260	-8.3
470	ZINC10130495	-9.7
471	ZINC18157214	-8
472	ZINC16640788	-9
473	ZINC01588760	-8.2
474	ZINC09353077	-9.2
475	ZINC09361088	-8.6
476	ZINC09109416	-8.4
477	ZINC09013879	-8

Pharmacophore No.10

ID	Identifier	Affinity (Kcal.mol^{-1})
1	ZINC03872623	-7.7
2	ZINC67912716	-8.6
3	ZINC29477570	-8
4	ZINC35270883	-8
5	ZINC03872622	-7.6
